# Effects of 5′,8′-Cyclo-2′-Deoxypurines on the Base Excision Repair of Clustered DNA Lesions in Nuclear Extracts of the XPC Cell Line

**DOI:** 10.3390/cells10113254

**Published:** 2021-11-20

**Authors:** Julia Kaźmierczak-Barańska, Karolina Boguszewska, Michał Szewczuk, Bolesław T. Karwowski

**Affiliations:** DNA Damage Laboratory of Food Science Department, Faculty of Pharmacy, Medical University of Lodz, 90-151 Lodz, Poland; julia.kazmierczak-baranska@umed.lodz.pl (J.K.-B.); karolina.boguszewska@umed.lodz.pl (K.B.); michal.szewczuk@umed.lodz.pl (M.S.)

**Keywords:** cdA, BER, BJ, XPC, DNA repair, DNA damage, clustered DNA lesions

## Abstract

Clustered DNA lesions (CDL) containing 5′,8-cyclo-2′-deoxypurines (cdPus) are an example of extensive abnormalities occurring in the DNA helix and may impede cellular repair processes. The changes in the efficiency of nuclear base excision repair (BER) were investigated using (a) two cell lines, one of the normal skin fibroblasts as a reference (BJ) and the second from *Xeroderma pigmentosum* patients’ skin (XPC), and (b) synthetic oligonucleotides with single- and double-stranded CDL (containing 5′,8-cyclo-2′-deoxyadenosine (cdA) and the abasic (AP) site at various distances between lesions). The nuclear BER has been observed and the effect of both cdA isomers (5′*R* and 5′*S*) presence in the DNA was tested. CdPus affected the repair of the second lesion within the CDL. The BER system more efficiently processed damage in the vicinity of the ScdA isomer and changes located in the 3′-end direction for dsCDL and in the 5′-end direction for ssCDL. The presented study is the very first investigation of the repair processes of the CDL containing cdPu considering cells derived from a *Xeroderma pigmentosum* patient.

## 1. Introduction

Eukaryotic cells have developed a system that ensures genome integrity, called the DNA damage response (DDR) pathway. Given that each human cell can develop approximately 10^2^–10^5^ lesions per day, multiple repair pathways are beneficial, e.g., mismatch repair (MMR), base excision repair (BER), nucleotide excision repair (NER), non-homologous repair end joining (NHEJ), or homologous recombination (HR) [1,2,3]. During DDR, cell cycle progression is blocked, and DNA damage sensors activate DNA repair mechanisms specific to the type of detected lesion. Single-stranded DNA breaks or oxidized nucleobases are mainly repaired by BER, large DNA adducts and cross-links are mainly repaired by NER, and minor nucleotide damage such as alkylation is repaired by MMR [4,5].

The harmful factors damaging cellular components include ionizing radiation. The lethal dose of 4 Gy of ionizing radiation can generate about 5000 single-strand breaks (SSBs), 160 DNA double-strand breaks (DSBs), 600 cross-links, or ~5000 DNA base damage [6]. Additionally, a low dose of 1 Gy of ionizing radiation can generate the above DNA damage in the form of isolated lesions as well as clustered DNA lesions (CDL) [7]. A single event of DNA irradiation may result in an additional inter- or intra-nucleotide linkage. This leads to the formation of bulky 5′,8-cyclo-2′-deoxypurines (cdPus) structures in DNA, where the base and sugar moiety of the purine nucleoside in DNA are simultaneously damaged. Therefore, cdPus have been classified as tandem changes which include two adjacent DNA changes located on the same strand [8,9,10]. The production yields of radiation-induced purine 5′,8-cyclo-2′-deoxyribonucleosides in human monocyte DNA were determined by HPLC-MS/MS of 20 and 4 changes per 10^9^ normal nucleosides per Gy for 5′,8-cyclo-2′-deoxyguanosine (cdG) and 5′,8-cyclo-2′-deoxyadenosine (cdA), respectively [11]. Other results indicate that 0.01 of 5′*R* and 0.1 of 5′*S* isomer of cdA occurs per every 10^6^ DNA nucleosides [12]. The estimation difficulty results from the fact that quantitative measurements of the cyclopurine lesions depend on the research technique or used cells and in irradiated samples results may differ even in studies conducted in the same laboratory, as results depend on the radiation dose, type, and age of the cells [13,14]. Tandem lesions are a special type of CDL [15]. Clustered lesions consist of two or more damaged nucleotides within 1–2 turns of the DNA duplex; they can include single-stranded as well as double-stranded lesions [16]. CDL can inhibit DNA repair and/or lead to an increase in the frequency of DSB formation [17,18]. The main pathway used by mammalian cells to remove bulky lesions in DNA is NER. It is implemented in two pathways—as global genome repair (GGR) and as transcriptional repair (TCR, which refers to damaged DNA that is actively transcribed). The GGR-NER includes numerous proteins, e.g., XPC/HR23B or XPE/DDR protein complexes (XP, *Xeroderma pigmentosum*; C, E—complementation groups C, E; UV excision repair protein RAD23) [19]. As a result of NER action, a DNA fragment of 27–32 nucleotides containing the damage is excised and reconstructed [19].

NER deficiencies in the human population are associated with mutations in the genes encoding the enzymes of this repair system. They are associated with an increased risk of skin cancer or with various developmental and degenerative changes. These mutations lead to diseases such as *Xeroderma Pigmentosum* (XP), Cockayne Syndrome (CS), or trichothiodystrophy (TTD) [20,21]. The health problems of XP patients vary in severity and the time of their appearance. XP is clinically manifested by sensitivity to UV light, abnormally thin skin, and an increased risk of skin cancer, especially in areas exposed to light. Among other symptoms, cataracts and neurological changes are also described [22,23]. The presence of mutations in the genes of various proteins in the NER pathway determines the existence of seven complementation groups, from XPA to XPG. The XPC group, from which cell extracts were obtained and used in the presented study, is characterized by the improper functioning of the GGR-NER. The XPC is the main protein responsible for GGR-NER initiation—it recognizes DNA damage that significantly distorts the DNA helix [21]. XPC is also associated with DNA alterations not repaired by NER [24]. The results presented by D’Errico et al. indicate that XPC can serve as a general DNA damage sensor and stimulate DNA repair also via BER. It may act as a cofactor in the repair of oxidative damage by base excision, stimulating the activity of its specific 8-oxo-guanine DNA glycosylase (OGG1) [25,26]. XPC may also contribute to the repair of cdPus or 8-hydroxyguanine and 8-hydroxyadenine.

The main biochemical pathway responsible for genome integrity is BER [27,28]. A unique feature of this path is the ability to remove a single base from the DNA strand [29]. The repair of most oxidative lesions (e.g., depurination, cytosine deamination, and guanine oxidation) is initiated by DNA glycosylases. They recognize the damaged base and cleave the *N*-glycosidic bond between the sugar and the damaged base, leaving an abasic site (AP site) or SSB, in the case of bifunctional glycosylases that also possess AP-lyase activity [30]. The AP site is recognized by the AP endonuclease which cleaves it from the 5′-side. Thereafter, repair can be accomplished by one of two pathways: a short-patch BER (SP-BER), where polymerase β (Polβ) inserts one missing nucleotide and the strand is fused by a ligase, or by a long-patch BER (LP-BER), where the free 3′OH-end of the cut DNA strand is extended by the replication apparatus (Polβ, Proliferating Cell Nuclear Antigen/Replication factor C subunit 1 (PCNA/RFC), Polδ or Polε) by up to 12 nucleotides, and the 5′-end is moved to the side [31,32,33].

Research by Brooks et al. has shown that massive cdPus damage is not repaired by BER as no cdPu-specific glycosylases are known [34]. The presence of any cdPu in the DNA helix inhibits its replication and transcription, but also significantly impedes DNA repair processes [35]. In addition, the occurrence of cyclic purines leads to chromatin aberration and the bypassing of such fragments during replication, which results in deletions and genetic instability [36].

It has been recently reported that the successful repair of minor lesions (e.g., AP site) within a CDL containing cdPus depends on the distance and the relative position between lesions [21]. Moreover, differences in BER activity were observed between 5′*S* and 5′*R* diastereomers and between purines (cdA and cdG). Repair of the dsCDL was tested in the nuclear extracts (NE) of xrs5 cells, as a well-established model for such experiments [1,10,37,38,39,40].

The presented study analyzed whether the presence of tandem lesions (RcdA or ScdA) and their relative distance from a single lesion (AP site) on the opposite or the same DNA strand affect the repairability of the latter. The main steps of the repair pathway (strand incision, elongation, and rejoining) were tested in NE of XPC cell line (human fibroblasts from XP patient, complementation group C) that carries the NER defect. As a reference system, the BJ cell line (human skin fibroblasts) was used, which represents normal cells with properly functioning DNA repair systems.

The presence of cdPus in DNA can lead to inhibition of gene expression or transcriptional mutagenesis. It is important to study the cdPus’ function to better understand its biological significance, its effects on DNA structure, and the key aspects of cell survival, such as the repair of damaged genetic material. It is also important to test whether the obtained observations are confirmed regardless of the cellular model involved in the research.

## 2. Materials and Methods

### 2.1. The Substrate Oligonucleotides

The oligonucleotides used as a substrate in the presented study were prepared on a Geneworld synthesizer (K&A Laborgeraete GbR, Schaafheim, Germany) in the Bioorganic Chemistry Department of the Polish Academy of Science (Lodz, Poland). Nucleotide phosphoroamidites needed for synthesis were purchased from the ChemGenes Corporation (Wilmington, MA, USA) while the phosphoroamidite cdPus’ derivatives were synthesized according to the previously described protocol [41]. The crude oligos were HPLC-purified (C-18 column, Phenomenex, Synergi 4 μm Fusion-RP 80 Å, 250 × 4.6 mm) and the concentration was measured (Varian Cary 1.3E spectrophotometer (Varian, Brunn am Gebirge, Austria)). Mass spectrometry analysis was performed in the full-scan negative-ion mode (mass range of 50–2000 *m*/*z*, Waters Synapt G2-Si HDMS, quadrupole time of flight hybrid mass spectrometer (Waters, Manchester, UK)). The results are presented in the Supplementary Materials, i.e., the found and calculated masses are reported in Table S1 and the mass spectra in Figure S2. The substrate oligos were stable in the conditions of the experiment as shown in previous studies: melting temperatures were above 70 °C [42] and matrix strands (native and containing cdPu) were not susceptible to the action of repair enzymes present in NE [2,10,40].

Table 1 shows the list of ds-oligonucleotides used in the experiments and their sequences. Each oligo contained 2′-deoxyuridine (dU) residue which has a specific number assigned. The number characterizes the number of base pairs between cdA and dU. For double-stranded lesions ([ds]), when dU was located in the 3′ direction in relation to cdA located on the opposite strand, it is described with a negative number; when dU was located in the 5′ direction in relation to cdA located on the opposite strand, it is described with a positive number. For single-stranded lesions ([ss]), when dU was located in the 5′ direction in relation to cdA located on the same strand, it is described with a negative number; when dU was located in the 3′ direction in relation to cdA located on the same strand, it is described with a positive number.

### 2.2. 5′-^32^P-End-Labeling of ss-Oligonucleotides

The 5′-^32^P-end-labeling of ss-oligos (40-mer, 230 pmol) was performed with T4 polynucleotide kinase (5U, New England BioLabs, Ipswich, MA, USA) and 2 μCi [γ-^32^P]ATP (3000 Ci/mmol, 10 mCi/mL, Hartmann Analytic GmbH, Braunschweig, Germany) in 20 μL of reaction buffer (70 mM Tris-HCl, 10 mM MgCl_2_, 5 mM DTT, pH 7.6 at 25 °C) at 37 °C for 30 min. The samples were denaturated at 95 °C for 5 min. The labeling of ss-oligos was verified, and purity was checked on 15% native polyacrylamide gel (Figure S1A).

### 2.3. Hybridization of ss-Oligonucleotides

The hybridization of radiolabeled ss-oligos was performed in pure H_2_O with a 1.5-fold excess of the suitable complementary strand (purified and non-radiolabeled, Table 1) at 90 °C for 10 min and was followed by slow cooling to room temperature. The ds-oligos were purified by precipitation (see Section 2.8). The hybridization was verified, and the purity was checked on the 15% native polyacrylamide gel (Figure S1A).

### 2.4. Preparation of ds-Oligonucleotides with AP Sites

The radiolabeled, dry ds-oligos with dU in their sequence were incubated with uracil-DNA glycosylase (UDG) (5U, New England BioLabs Ipswich, MA, USA), which recognizes and removes dU from DNA. Reactions were performed in 20 μL of the reaction buffer (20 mM Tris-HCl, 1 mM EDTA, 1 mM DTT, pH 8.0 at 25 °C) at 37 °C for 30 min. The ds-oligos containing AP sites were purified by precipitation (see Section 2.8). Additionally, the AP sites formation was verified. The ds-oligos with AP sites were incubated with human apurinic/apyrimidinic endonuclease (APE1) (5U, New England BioLabs Ipswich, MA, USA) in 10 μL of the reaction buffer (50 mM potassium acetate, 20 mM Tris-acetate, 10 mM magnesium acetate, 1 mM DTT, pH 7.9 at 25 °C) at 37 °C for 30 min to generate SSBs visible as shorter DNA fragments with a length of 15–25 nucleotides. The purity and formation of SSBs were checked on 15% denaturing polyacrylamide gel (Figure S1B).

### 2.5. Cell Cultures

The study was performed using two cell lines—BJ as a reference cell line and XPC as a tested cell line. The BJ, human fibroblasts cell line (CRL-2522, ATCC, Vancouver, CA, USA) was cultured in MEM with Earle’s salts and non-essential amino acids supplemented (Gibco) with 10% FBS (Biowest, MO, USA). Originally cultured from neonatal foreskin tissue, the BJ cell line is a recognized model of replicative senescence [43]. It results from the Hayflick limit or exhaustion of the division limit and affects most types of somatic cells in vitro and in vivo. It is caused by the shortening of the telomeres. As the primary cell line, it was adopted in this study as the reference line with properly functioning cellular DNA repair processes.

Cells of the XP patient belonging to the XPC group (GM17420 skin fibroblasts) were purchased from the Coriell Institute for Medical Research (Camden, NJ, USA). Clinical, cellular, and molecular data for GM17420 is available upon request from the Coriell Institute. XPC fibroblasts were cultured in MEM with Earle’s salts and non-essential amino acids (Gibco) and supplemented with 15% FBS (Biowest, MO, USA). Compared to normal fibroblasts, XP cells are hypersensitive to UV radiation and/or chemical factors, which results in a significantly increased frequency of chromosome breaks [23]. Moreover, the damage lasts longer, which indicates impaired repair processes. Measurements of the efficiency of cell repair systems showed that XPC cells had 10–20% of the repair synthesis shown by normal cells [44].

### 2.6. Preparation of Nuclear Extracts

Cells from each cell line were harvested in the exponential phase. After pelleting, they were treated with the NE-PER™ Nuclear and Cytoplasmic Extraction Reagents kit (ThermoFisher Scientific, Waltham, MA, USA) according to the protocol provided by the manufacturer. The concentration of nuclear extracts (NE) was determined using the Pierce™ 660 nm Protein Assay colorimetric assay (ThermoFisher Scientific, Waltham, MA, USA). Concentrations were found as follows: BJ—between 5.4–8.5 mg/mL; XPC—between 4.2–6.4 mg/mL. Aliquots of cellular extracts were stored at −80 °C for a maximum period of 6 months.

### 2.7. Repair Assays

The radiolabeled ds-oligos (200 cps) were incubated with 10 μg of NE in the repair buffer (70 mM Tris-HCl (pH 7.5), 40 mM phosphocreatine, 10 mM DTT, 5 mM MgCl_2_, 4 mM ATP, 1.6 μg/mL CK, and 0.1 mM of each dATP, dCTP, dGTP, and dTTP) at 37 °C for 0, 1, 5, 15, 30, 60, 90, and 120 min. The amount of NE used in repair assays was optimized from titration studies (data not shown). The reactions were stopped and examined on 15% denaturing polyacrylamide gel (see Section 2.8).

Each set of reactions was repeated three times for its consistency and reliability. Data were quantified using Quantity One (Bio-Rad, Hercules, CA, USA) where the AP site repair in relation to reaction time was determined. The intensity of the bands was analyzed and disclosed as a % value of the overall intensity of bands within individual lanes on the autoradiogram. The slight differences in the repair activity between batches of NE were taken into account; hence, all results were compared with the control containing AP site as single damage.

### 2.8. General Procedures

Purification of oligonucleotides: 250 μL of ice-cold ethanol was added to samples which were incubated at −80 °C for 30 min and subsequently centrifuged at 4 °C for 30 min (13,000 rpm). Afterward, the samples were dried under reduced pressure at room temperature and dissolved in pure H_2_O.

Native PAGE: reactions were stopped by placing the samples on ice and adding the native loading buffer (40% sucrose, 0.025% bromophenol blue, and 0.025% xylene cyanole). Samples were subjected to electrophoresis on a 15% native polyacrylamide gel in 1× TBE (89 mM Tris-HCl, 89 mM boric acid, 2 mM EDTA).

Denaturing PAGE: reactions were stopped by placing the samples on ice and adding the denaturing loading buffer (95% formamide, 2 mM EDTA, 0.025% bromophenol blue, and 0.025% xylene cyanole). Samples subjected to electrophoresis on a 15% denaturing polyacrylamide gel with 8M urea in 1× TBE (89 mM Tris-HCl, 89 mM boric acid, 2 mM EDTA).

The PAGE electrophoresis was performed at a constant power of 45 W for 120 min. The obtained results were visualized by autoradiography.

## 3. Results

The study examined whether the diastereomeric form of cdA and the distance between lesions located within single- or double-stranded clustered DNA damage (ssCDL or dsCDL) had an impact on the repair processes. Moreover, this work tried to answer the often-raised question about the influence of the clustered DNA lesions on the DNA repair in *Xeroderma Pigmentosum* patients. The two cell lines were used—BJ (human skin fibroblasts) and XPC (skin fibroblasts from *Xeroderma Pigmentosum* patient, complementation group C, see Section 2.5). The experimental model was, as previously described, synthetic double-stranded oligonucleotides containing dU (AP site precursor) and 5′,8-cyclo-2′-deoxyadenosine (cdA) (5′*S* and 5′*R* isomer) on the same (ssCDL) or the opposite strand (dsCDL) (Table 1) [10,40,42]. Each step of the substrate ds-oligonucleotide preparation was verified, and the results are presented in Figure S1. As shown previously, both diastereomers of cdA are stable in experimental conditions, and during reactions with NE and chosen glycosylases [1,2,10,40,42].

NE obtained from BJ and XPC cell lines were used to determine the repair efficiency of AP sites located within ssCDL and dsCDL. The goal was to examine the main stages of BER (AP site incision, strand elongation, and strand rejoining) taking place in the nucleus of XPC cells (compared to BJ as a reference cell line).

**Figure 1:**
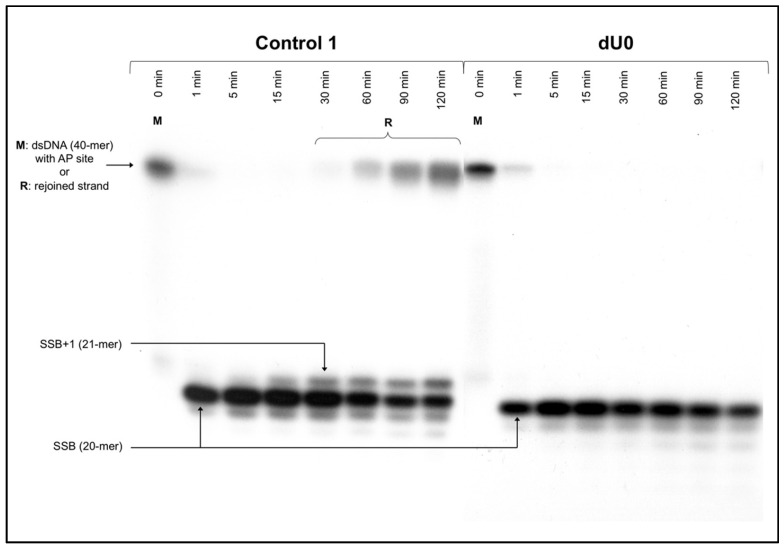
The representative autoradiogram presenting stages of nuclear BER in XPC cell line (the strand incision, elongation, and repair) of control dsDNA: Control 1—dsDNA with a single AP site on one strand (positive control); dU0—dsDNA with AP site and ScdA or RcdA opposite to each other on two strands (negative control); M—length marker (40-mer dsDNA with a single AP site); R—rejoined strand (40-mer dsDNA resulting from repair activity of nuclear extract); SSB—single-strand break (AP site cleavage resulting from the endonucleolytic activity of nuclear extract); SSB+1—cleaved strand with 1 nucleotide unit incorporated (resulting from polymerase activity of nuclear extract). Each experiment was performed three times for consistency. Autoradiograms of all experimental repeats are available in the Supplementary Materials.

Control 1 (containing a single AP site, Table 1) was used to confirm the activity of proteins in the NE (Figure 1). In the case of the reference cell line (BJ), the endonucleolytic activity (strand incision) was observed after 1 min, and polymerase activity was detected after 5 min (Table S5). AP site rejoining (strand reconstitution) was observed after 30 min, increasing with time up to 82.98 ± 7.68% after 120 min (Table S4). The XPC cells showed endonucleolytic activity after 1 min, polymerase activity after 15 min, and strand rejoining after 30 min, reaching 32.96 ± 7.65% after 120 min (Table S2).

The presented study analyzed whether the presence of tandem lesions (RcdA or ScdA) and their relative distance of 1–5 nucleobases in 3′-end and 5′-end direction from a single lesion (AP site) on the opposite or the same strand affected the cell’s ability to repair the latter. The repair was tested in NE obtained from XPC and BJ cells. Moreover, it was investigated whether the XPC defect will affect the repair processes carried out by BER and if it would be an additional factor (next to cdPus) limiting the repair of small lesions such as AP sites.

### 3.1. The Influence of Single-Stranded CDL on the Nuclear BER Pathway in XPC Cells

The impact of the cdA appearing in the genome on the DNA repair pathways is widely studied [1,2,8,10,40,45,46,47]. Single-stranded CDL (ssCDL) containing ScdA or RcdA have been shown to impair the activity of specific proteins involved in the BER process, e.g., glycosylases and polymerases [10,37,38,39,42,48]. This study explored the repair via BER (strand incision, elongation, and rejoining) in the NER-deficient cell line derived from the XP patient.

#### 3.1.1. Strand Incision

**Figure 2:**
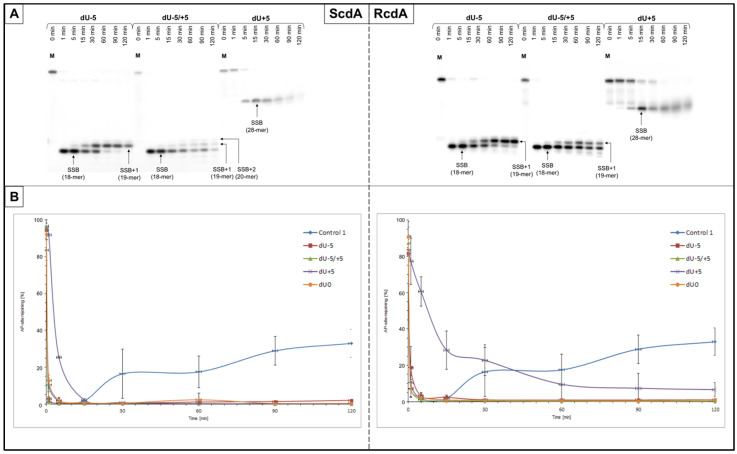
(**A**) The representative autoradiograms presenting stages of nuclear BER in XPC cell line (the strand incision, elongation, and repair) of dsDNA containing single-stranded clustered DNA lesions (ssCDL) with AP site and ScdA or RcdA on one strand distanced by 5 base pairs in 5′-end direction (negative numbers) or 3′-end direction (positive numbers); M—length marker (40-mer dsDNA with a single AP site); SSB—single-strand break (AP site cleavage resulting from the endonucleolytic activity of nuclear extract); SSB+1/SSB+2—cleaved strand with 1 or 2 nucleotide unit incorporated (resulting from polymerase activity of nuclear extract). Each experiment was performed three times for consistency. (**B**) Graphical representation of the AP site rejoining trends (strand reconstitution). Graphs in higher resolution and autoradiograms of all experimental repeats are available in the Supplementary Materials.

The incision of the AP sites resulting from the endonucleolytic activity of NE was observed for all tested substrates. SSBs were observed as bands corresponding to 18-mer for dU−5 and dU−5/+5, and to 28-mer for dU+5 (Figure 2 and Figures S3A–S6A).

The SSB formation for ScdA/dU−5 and ScdA/dU−5/+5 was the most efficient after 1 min, which was higher compared to the Control 1 (89.78 ± 2.93% and 88.04 ± 3.72%, respectively, vs. 56.32 ± 8.99% for Control 1) (Figure S5A, Tables S2 and S3). However, for ScdA/dU+5, it took longer, i.e., 15 min, to reach maximal strand incision (73.54 ± 7.18%, Table S3). Interestingly, in the case of RcdA the activity of endonucleases was lower: 63.21 ± 10.10% after 5 min for RcdA/dU−5, 73.51 ± 10.96% after 1 min for RcdA/dU−5/+5, and 40.35 ± 7.13% after 30 min for RcdA/dU+5 (Figure S6A, Table S4).

As expected, the reference cell line (BJ) demonstrated the same trends as XPC but reached higher levels of total endonucleolytic activity, 79.08 ± 5.18% after 1 min for Control 1, from 78.88 ± 3.41% to 91.43 ± 3.15% after 1–15 min for ScdA, and from 77.15 ± 6.97% to 85.38 ± 4.28% after 1–5 min for RcdA (Figures S8A and S9A, Tables S5–S7).

The overall trends for strand incision (for both cell lines) were as follows: dU−5/+5~dU−5 > dU+5 (for ScdA) and dU−5/+5 > dU−5 > dU+5 (for RcdA).

#### 3.1.2. Strand Elongation

The activity of polymerases was also taken under consideration for both cell lines. Incorporation of up to two nucleotide units (NUs) was observed for XPC suggesting that the LP-BER pathway was activated (Figure 2 and Figures S3 and S4, parts A). The same observation was made for BJ, where two NUs were incorporated (Figures S8 and S9).

The polymerase activity in XPC cells increased with time, reaching its maximums after 120 min—87.47 ± 6.21% for ScdA/dU−5, 41.88 ± 2.18% for ScdA/dU−5/+5, 33.02 ± 4.38% for ScdA/dU+5, 71.35 ± 7.22% for RcdA/dU−5, and 44.51 ± 11.71% for RcdA/dU+5 (Tables S3 and S4). The only exception was observed for RcdA/dU−5/+5, where the maximum of 41.13 ± 3.76% was achieved after 60 min instead of 120 min (Table S4). Interestingly, all tested oligos demonstrated higher activity than Control 1 (22.24 ± 4.30% after 120 min, Table S2). The overall activity of polymerases after 30 min was approximately 8% higher for ScdA/dU−5 than RcdA/dU−5. Oligos with RcdA/dU−5/+5 and RcdA/dU+5 reached higher values than those with ScdA—approximately 5 and 8%, respectively (Figure 2 and Figrues S3A–S6A, Tables S3 and S4).

The reference BJ cell line showed higher (for ScdA/dU−5/+5, ScdA/dU+5 and RcdA/dU−5) or similar (for ScdA/dU−5, RcdA/dU−5/+5, and RcdA/dU+5) levels of polymerase activity than XPC, which was expected. The overall trend of strand elongation efficiency by polymerases for all tested variants was as follows: dU−5 > dU−5/+5 > dU+5 (for ScdA and RcdA; XPC, and BJ).

#### 3.1.3. Strand Reconstitution

The strand ligation was not observed in NE of XPC for both cdA isomers compared to Control 1, where it reached 32.96 ± 7.65% after 120 min (Figure 2 and Figures S5 and S6, Tables S2–S4). Interestingly, little or no repair was observed for BJ cells—only ScdA/dU−5 showed an inconsiderable strand reconstitution (7.78 ± 4.23%) compared to Control 1 (82.98 ± 7.68% after 120 min) (Figures S8A–S11A, Tables S5–S7). In general, BJ cells were coping better with ssCDL containing cdA than XPC (Figures S13 and S14).

### 3.2. The Influence of Double-Stranded CDL on the Nuclear BER Pathway in XPC Cells

As shown previously for xrs5 cell line, dsCDL containing ScdA or RcdA impact the main stages of the BER pathway, i.e., strand incision, elongation, and reconstitution [40]. In this study, those stages of repair via base excision were further examined in the context of nuclear extracts of the NER-deficient XPC cell line.

#### 3.2.1. Strand Incision

In the case of dsCDL, the endonucleolytic activity of NE was noted for all tested oligonucleotides as SSBs, which were observed on autoradiograms as bands corresponding to the following strand lengths: 19-mer (dU−1), 16-mer (dU−4), 21-mer (dU+1), and 24-mer (dU+4) (Figure 3 and Figures S3B–S6B and S8B–S11B).

For the XPC cells, strands containing ScdA were incised with efficiency above 70% after following times: 1 min for ScdA/dU+1; 5 min for ScdA/dU−1 and ScdA/dU−4; and 15 min for ScdA/dU+4 (Figure S5B, Table S3). Interestingly, overall incision yields for ScdA were higher than for Control 1 (57.83 ± 7.31% after 5 min, Table S2). For oligos with RcdA, the incision efficiencies were lower than Control 1 (Figure S6, Tables S2 and S4). The following AP site incision rates were noted: 50.23 ± 18.55% after 5 min for RcdA/dU+1; 52.18 ± 19.25% after 5 min for RcdA/dU−4; 48.64 ± 5.15% after 5 min for RcdA/dU−1; and 23.16 ± 3.63% after 15 min for RcdA/dU+4 (Figure S6B, Table S4). In the case of XPC, the presence of RcdA inhibited the endonucleolytic activity of the NE approximately 20–50% more in comparison to ScdA. The overall incision rates for XPC were increasing in the following order (data compared for 1 min of reaction time): +4 < −1 < −4 < +1 (for both isomers).

On the other hand, the strands were incised with higher efficiency for BJ than XPC, but with slightly different incision trends. Only for ScdA/dU−4, SSBs were formed more efficiently than for Control 1 (92.16 ± 7.40% vs. 79.08 ± 5.18% after 1 min, respectively) (Tables S5–S7). The 5′*R* isomer achieved maximal endonucleolytic activity 4–20% lower than 5′*S* (except for AP site positioned −1 to cdA, where it was hydrolyzed 5% better for RcdA than ScdA) (Figures S10B and S11B, Tables S6 and S7). The rate of strand incision for BJ increased in the following order (data compared for 1 min reaction time): for ScdA +4 < −1 < +1 < −4; for RcdA +4 < −1 < −4 < +1 (Figures S8B–S11B, Tables S5–S7).

#### 3.2.2. Strand Elongation

The efficiency of strand elongation by polymerases was taken under consideration for dsCDL. The activity of SP-BER has been detected (incorporation of 1NU) for most examined substrates (both cell lines). No polymerase activity (no SSB+1 bands) was observed for lesions positioned opposite (position dU0) and +1 to cdA (dU+1) for all tested variants.

**Figure 3:**
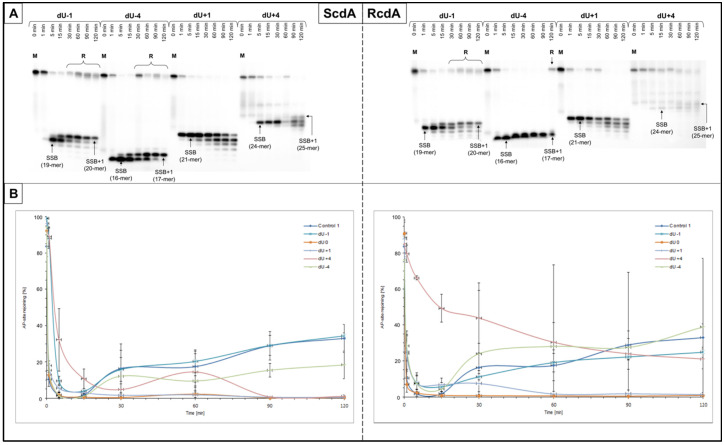
(**A**) The representative autoradiograms presenting the nuclear BER in XPC cell line (the strand incision, elongation, and repair) of dsDNA containing double-stranded clustered DNA lesions (dsCDL) with AP site and ScdA or RcdA on opposite strands distanced by 1–4 base pairs in 3′-end direction (negative numbers) or 5′-end direction (positive numbers); M—length marker (40-mer dsDNA with a single AP site); R—rejoined strand (40-mer dsDNA); SSB—single-strand break (AP site cleavage resulting from the endonucleolytic activity of nuclear extract); SSB+1/SSB+2—cleaved strand with 1 or 2 nucleotide unit incorporated (resulting from polymerase activity of nuclear extract). Each experiment was performed three times for consistency. (**B**) Graphical representation of the AP site rejoining trends (strand reconstitution). Graphs in higher resolution and autoradiograms of all experimental repeats are available in the Supplementary Materials.

Polymerases present in XPC showed higher rates of DNA synthesis for dU−1 and dU−4 (both isomers) than Control 1 (ScdA/dU−1: 42.17 ± 0.62%, ScdA/dU−4: 44.19 ± 10.44%; RcdA/dU−1: 28.87 ± 4.23%, and RcdA/dU−4: 42.15 ± 23.91% vs. 20.93 ± 3.87% after 30 min for Control 1). The dU+1 (both isomers) and ScdA/dU+4 did not reach 10%, while RcdA/dU+4 almost reached the control level (Figure 3 and Figures S3B–S6B, Tables S2–S4). Moreover, the maximal yields were approximately 9–22% lower for RcdA than ScdA in the XPC cell line. Interestingly, RcdA/dU−1 and RcdA/dU−4 achieved the maximum level of strand elongation after 60 min, while corresponding positions for ScdA reached their maximums after 90 min. The efficiency of DNA synthesis in XPC cells increased in the following order (data compared for 30 min of reaction time): +4 < −1 < −4 (both isomers). Worth noting is the fact that despite the same trends, the 5′*R* isomer inhibited the BER process by approximately 9–28% more than 5′*S*.

The trends of polymerases efficiency in the BJ cell line were similar to those observed in the XPC (Figures S8B–S11B and Tables S5–S7). The rates of gap-filling for both isomers in the case of dU−1 and dU−4 were approximately 17–28% higher than for Control 1 (25.48 ± 2.15% after 30 min). Furthermore, RcdA/dU−1 inhibited enzyme’s action approximately 10% stronger than ScdA/dU−1. Oligos with dU−4 reached levels close to the control, but after the different times (ScdA/dU−4: 68.67 ± 3.68% after 60 min; RcdA/dU−4: 69.37 ± 0.68% after 90 min, Tables S5–S7).

Interestingly, for BJ, dU+4 polymerases were approximately two-fold more efficient after 120 min than in XPC (BJ: 43.97 ± 1.27% for ScdA/dU+4 and 51.47 ± 3.54% for RcdA/dU+4 compared to XPC: 17.57 ± 2.38% for ScdA/dU+4 and 25.00 ± 10.62% for RcdA/dU+4). In general, the reference cell line showed maximal polymerase activity rates higher than XPC, which was the anticipated result. The exception was observed for ScdA/dU−1 and ScdA/dU−4 where the gap-filling was similar for both cell lines.

The overall trend for the DNA synthesis by polymerases increased in the following order (data compared for 30 min of reaction time): +4 < −1 < −4 (for ScdA and RcdA; XPC, and BJ).

#### 3.2.3. Strand Reconstitution

The influence of cdA on the strand rejoining was also considered for dsCDL (Figure 1 and Figure 3, Figures S3B–S6B, S7, S13 and S14, Tables S2–S4). The strand ligation was not observed for gaps denoted as dU0 and dU+1 in all tested variants. Moreover, in the case of XPC, ScdA/dU+4 showed no repair while for RcdA/dU+4 strand was not fully incised preventing its reconstitution (Figures S3B–S6B). ScdA/dU−1 and RcdA/dU−1 were repaired on the control level (20.30 ±6.34% and 19.10 ± 5.17%, respectively vs. 17.44 ± 8.56% after 60 min for Control 1) (Tables S2–S4). Interestingly, ScdA/dU−4 showed 9.49 ± 2.85% of strand rejoining, which was lower than Control 1 (Tables S2 and S3).

On the other hand, the reference cell line (BJ) showed higher rates of strand reconstitution than XPC for ScdA/dU−1, RcdA/dU−1, and ScdA/dU−4 (Figures S13 and S14). Conversely, RcdA/dU−4 was repaired better for XPC than BJ (Figure S14). For BJ, oligos denoted as ScdA/dU−1, and ScdA/dU−4 after 60 min were rejoined less efficiently than the control (26.55 ± 2.91% and 23.12 ± 7.32%, respectively vs. 44.70 ± 10.13% for Control 1). However, the values increased with time and reached the control level after 90 min (43.52 ± 2.41% for ScdA/dU−1 and 45.22 ± 12.17% for ScdA/dU−4). Oligos with 5′*R* diastereomer showed different trends in BJ cells. RcdA/dU−1 reached the repair level of 76.72 ± 5.73% after 120 min (compared to 82.98 ± 7.68% after 120 min for Control 1), while RcdA/dU−4 reached only 18.26 ± 6.44% after the same time. The dU−1 was repaired better when a 5′*R* isomer was present in the oligonucleotide’s sequence, whereas dU−4 showed the opposite result (Figure S12).

The overall rejoining efficiency increased in the following order for XPC: −4 < −1 (ScdA) and −1 < −4 (RcdA) and for BJ: −1 < −4 (both isomers).

## 4. Discussion

The presented study investigated how (5′*S*) and (5′*R*) 5′,8-cyclo-2′-deoxyadenosine affect the BER repair of ssCDL and dsCDL in NE of XPC cell line. XPC cells obtained from a patient with *Xeroderma pigmentosum* are NER-deficient. Given that cdPus are not a substrate for BER, this study focused on the second lesion (AP site) present in ssCDL or dsCDL and on the repair efficiency demonstrated by the total activity of nuclear BER proteins.

BER repair enzymes that excise isolated lesions are inhibited when the damage is a part of double- or single-stranded clusters. Previously presented observations confirmed that shifting the distribution of lesions by one nucleotide may change the course of repair or stop it [10,40]. An important observation was the fact that the first stages of the BER system worked more efficiently on oxidative damage (AP site) if it was located within ssCDL or dsCDL systems with the ScdA isomer. This is interesting because, with the rather limited efficiency of the NER system in removing cdPus, excision of 5′*R* isomer was more efficient than 5′*S* [12,13,49].

Moreover, as expected, the reference BJ cell line showed the same trends and achieved a higher enzymatic activity of nuclear extracts than XPC. The strand incision and reconstitution were more efficient for BJ, while polymerase activity showed similar or higher levels for BJ than for XPC. Our observations indicate that CDL is also a problem for BJ cells, confirming other results showing that clustered DNA damage is more difficult to repair for the cellular machinery than single lesions [50].

The results showed a similar strand incision trend for XPC and xrs5 cell lines; total endonucleolytic efficiency of NE was higher in the presence of ScdA for both ssCDL and dsCDL [40]. Consistent with the earlier observations obtained for line xrs5, lesions located in the 5′-end direction (positive numbers) of RcdA and ScdA were repaired less efficiently than those located in the 3′-end direction (negative numbers) for dsCDL. Polymerase activity was also observed. It was inhibited by the presence of RcdA even up to 28% more than for ScdA (for both, ssCDL and the dsCDL). It also confirms previous results obtained for xrs5 where the polymerase activity towards RcdA was lower by about 8–20% for most of the tested substrates. Additionally, the polymerase activity was higher in the case of the lesions located in the 3′-end direction for dsCDL ScdA/dU−4 (~44%), where a rate of DNA synthesis of several percent was observed for changes in the 5′-end direction. Again, this supported the observations from xrs5 where DNA synthesis efficiency increased towards the 3′-end [40]. However, results were different for ssCDL, where the polymerase activity was higher for the lesions directed towards 5′-end. The highest activity for XPC extracts was observed in ssCDL for ScdA/dU−5 (~87%), which confirms the observations for BER activity towards ssCDL in extracts obtained from xrs5 [10]. Nevertheless, the small differences in the repair for the dsCDL are very interesting. The extracts from all three discussed lines (BJ, XPC, and xrs5) most efficiently repaired the lesion shifted by 1 NU towards 3′-end for the ScdA and then ScdA/dU−4 (dU−1 > dU−4). The minor differences relate to the repairability of the RcdA isomer. In the case of the BJ reference cell line, the repair trend is the same as for the S isomer (dU−1 > dU−4). However, with a reduced repair efficiency in XPC, the best strand reconstruction was observed for the RcdA/dU−4, the repair of which is blocked in the case of xrs5 extracts.

By comparing the results obtained for XPC and previously for xrs5, the common trends emerged: (a) the BER system processes lesions in the vicinity of the ScdA more efficiently than RcdA which probably results from the fact that 5′*R* isomer distorts DNA helix to a higher degree than 5′*S* [51] and (b) lesions located in the 3′-end direction for dsCDL and the 5′-end direction for ssCDL are processed better by BER nuclear proteins, which confirms previous studies (Table 2).

## 5. Conclusions

In summary, the presented results indicate that the BER repair system in XPC nuclear extracts can repair the oxidative damage that constitutes CDL along cdPu. The efficiency and degree of repair depend on the mutual distance of individual lesions within the cluster, diastereomeric form of cdA, and the lesion arrangement on DNA strands (single- or double-stranded clustered DNA damage). This may explain why some lesions are more durable and their repair in the cell is delayed.

Moreover, the presented results confirm what was observed for xrs5 nuclear extracts. This fact additionally validates the use of the xrs5 model for the clustered DNA lesion studies. From the technical point of view, it is beneficial, because working with xrs5 cells is cheaper, more convenient, easier, and faster.

Detailed knowledge on how the distribution of damage within the CDL affects the efficiency of repair processes may have further application in radiotherapy, e.g., in determining the therapeutic dose of radiation. Moreover, it may explain why XP develops various clinical phenotypes which are difficult to predict. The accumulation of cdPus observed in XP patients may be the cause of the development and worsening of XP symptoms and/or other neurological diseases [52,53]. CdA diastereomers are repaired diversely; therefore, it is not surprising that the ScdA and RcdA affect BER in a different way. To understand the potential biological consequences of CDL, it is imperative to carefully recognize what happens to CDL in cells with physiological levels of repair enzymes.

## Figures and Tables

**Table 1:** The list of substrate ds-oligonucleotides containing 2′-deoxyuridine (dU) and 5′,8-cyclo-2′-deoxyadenosine (cdA).

Oligonucleotide	Sequence
Control 1	5′-CTCTTGTCAGGAATATTGTC**U**CTATGCTCCCACCAAAGGC-3′ 3′-GAGAACAGTCCTTATAACAGAGATACGAGGGTGGTTTCCG-5′
[ds] dU−4	5′-CTCTTGTCAGGAATAT**U**GTCTCTATGCTCCCACCAAAGGC-3′ 3′-GAGAACAGTCCTTATAACAG**X**GATACGAGGGTGGTTTCCG-5′
[ds] dU−1	5′-CTCTTGTCAGGAATATTGT**U**TCTATGCTCCCACCAAAGGC-3′ 3′-GAGAACAGTCCTTATAACAG**X**GATACGAGGGTGGTTTCCG-5′
[ds] dU0	5′-CTCTTGTCAGGAATATTGTC**U**CTATGCTCCCACCAAAGGC-3′ 3′-GAGAACAGTCCTTATAACAG**X**GATACGAGGGTGGTTTCCG-5′
[ds] dU+1	5′-CTCTTGTCAGGAATATTGTCT**U**TATGCTCCCACCAAAGGC-3′ 3′-GAGAACAGTCCTTATAACAG**X**GATACGAGGGTGGTTTCCG-5′
[ds] dU+4	5′-CTCTTGTCAGGAATATTGTCTCTA**U**GCTCCCACCAAAGGC-3′ 3′-GAGAACAGTCCTTATAACAG**X**GATACGAGGGTGGTTTCCG-5′
[ss] dU−5	5′-CTCTTGTCAGGAATATTG**U**CTCT**X**TGCTCCCACCAAAGGC-3′ 3′-GAGAACAGTCCTTATAACAGAGATACGAGGGTGGTTTCCG-5′
[ss] dU−5/+5	5′-CTCTTGTCAGGAATATTG**U**CTCT**X**TGCT**U**CCACCAAAGGC-3′ 3′-GAGAACAGTCCTTATAACAGAGATACGAGGGTGGTTTCCG-5′
[ss] dU+5	5′-CTCTTGTCAGGAATATTGTCTCT**X**TGCT**U**CCACCAAAGGC-3′ 3′-GAGAACAGTCCTTATAACAGAGATACGAGGGTGGTTTCCG-5′

[ds]—double-stranded lesions (located on the opposite DNA strands); [ss]—single-stranded lesions (located on the same DNA strand); U—2′-deoxyuridine as an AP site precursor (after treatment with UDG; see Section 2.5); X—(5′*S*)-5′,8-cyclo-2′-deoxyadenosine (ScdA) or (5′*R*)-5′,8-cyclo-2′-deoxyadenosine (RcdA).

**Table 2:** The observed trends of the main stages of nuclear BER in XPC cell line for single-stranded (ssCDL) and double-stranded (dsCDL) clustered DNA lesions containing AP site and 5′*S* or 5′*R* isomer of 5′,8-cyclo-2′-deoxyadenosine (ScdA or RcdA). Substrate annotations and oligonucleotide sequences are presented in Table 1.

		ScdA	RcdA
**ssCDL**	Strand incision	dU+5 < dU−5/+5 ~ dU−5	dU+5 < dU−5 < dU−5/+5
	Strand elonagation	dU+5 < dU−5/+5 < dU−5	
	Strand reconstitution	not observed	
**dsCDL**	Strand incision	dU+4 < dU−1 < dU−4 < dU+1	
	Strand elonagation	dU+4 < dU−1 < dU−4	
	Strand reconstitution	dU−4 < dU−1	dU−1 < dU−4

## Data Availability

Not applicable.

## Supplementary Materials

The following are available online at https://www.mdpi.com/article/10.3390/cells10113254/s1, Figure S1: Verification of the ss-oligos labeling, hybridization, AP sites’ formation (after UDG treatment), and SSBs’ formation (after hAPE1 treatment), Figure S2: The results of mass spectrometry analysis of chosen substrate oligonucleotides containing cdA, Figures S3 and S4: The autoradiograms presenting repair of ss-CDL and ds-CDL—XPC, Figures S5 and S6: Graphical representation of repair assays’ results for XPC, Figure S7: AP site rejoining [%] of ScdA vs. RcdA—comparison of individual strands—XPC, Figures S8 and S9: The autoradiograms presenting repair of ss-CDL and ds-CDL—BJ, Figures S10 and S11: Graphical representation of repair assays’ results for BJ, Figure S12: AP site rejoining [%] of ScdA vs. RcdA—comparison of individual strands—BJ, Figures S13 and S14: AP site rejoining [%] of BJ vs. XPC—comparison of individual strands, Table S1: The masses of oligonucleotides, Tables S2–S4: Numerical data—XPC, Tables S5–S7: Numerical data—BJ.
