# Supplementary material for: Effects of 5′,8′-Cyclo-2′-Deoxypurines on the Base Excision Repair of Clustered DNA Lesions in Nuclear Extracts of the XPC Cell Line
Source: Cells. 2021 Nov 20;10(11):3254. doi: 10.3390/cells10113254 (PMC8618216; doi:10.3390/cells10113254)
Supplement: Supplementary file 1 [file cells-10-03254-s001.zip › cells-1406731-supplementary.pdf]

Effects of 5',8'-cyclo-2'-deoxypurines on the Base Excision Repair of Clustered DNA Lesions in Nuclear Extracts of XPC Cell Line

Supplementary Materials

Page	Item	Title
2	**Figure S1**	Verification of the ss-oligos labeling, hybridization, AP sites' formation (after UDG treatment) and SSBs' formation (after hAPE1 treatment)
2	**Table S1**	The calculated vs. found masses of chosen substrate oligonucleotides
3-6	**Figure S2**	Results of mass spectrometry analysis of chosen substrate oligonucleotides containing cdA
7-8	**Figure S3**	The autoradiograms presenting repair of ss-CDL and ds-CDL containing **ScdA** – XPC Nuclear Extract
9-10	**Figure S4**	The autoradiograms presenting repair of ss-CDL and ds-CDL containing **RcdA** – XPC Nuclear Extract
11-13	**Figure S5**	Graphical representation of repair assays' results for ScdA – XPC Nuclear Extract
14	**Table S2**	Numerical data – Control 1 – XPC Nuclear Extract
15-17	**Table S3**	Numerical data – ScdA – XPC Nuclear Extract
18-20	**Figure S6**	Graphical representation of repair assays' results for RcdA – XPC Nuclear Extract
21-23	**Table S4**	Numerical data – RcdA – XPC Nuclear Extract
24-27	**Figure S7**	AP site rejoining [%] of ScdA vs. RcdA – comparison of individual strands – XPC Nuclear Extract
28-29	**Figure S8**	The autoradiograms presenting repair of ss-CDL and ds-CDL containing **ScdA** – BJ Nuclear Extract
30-31	**Figure S9**	The autoradiograms presenting repair of ss-CDL and ds-CDL containing **RcdA** – BJ Nuclear Extract
32-34	**Figure S10**	Graphical representation of repair assays' results for ScdA – BJ Nuclear Extract
35	**Table S5**	Numerical data – Control 1 – BJ Nuclear Extract
36-38	**Table S6**	Numerical data – ScdA – BJ Nuclear Extract
39-41	**Figure S11**	Graphical representation of repair assays' results for RcdA – BJ Nuclear Extract
42-44	**Table S7**	Numerical data – RcdA – BJ Nuclear Extract
45-48	**Figure S12**	AP site rejoining [%] of ScdA vs. RcdA – comparison of individual strands – BJ Nuclear Extract
49-52	**Figure S13**	AP site rejoining [%] of BJ vs. XPC – comparison of individual strands – ScdA Nuclear Extract
53-56	**Figure S14**	AP site rejoining [%] of BJ vs. XPC – comparison of individual strands – RcdA Nuclear Extract

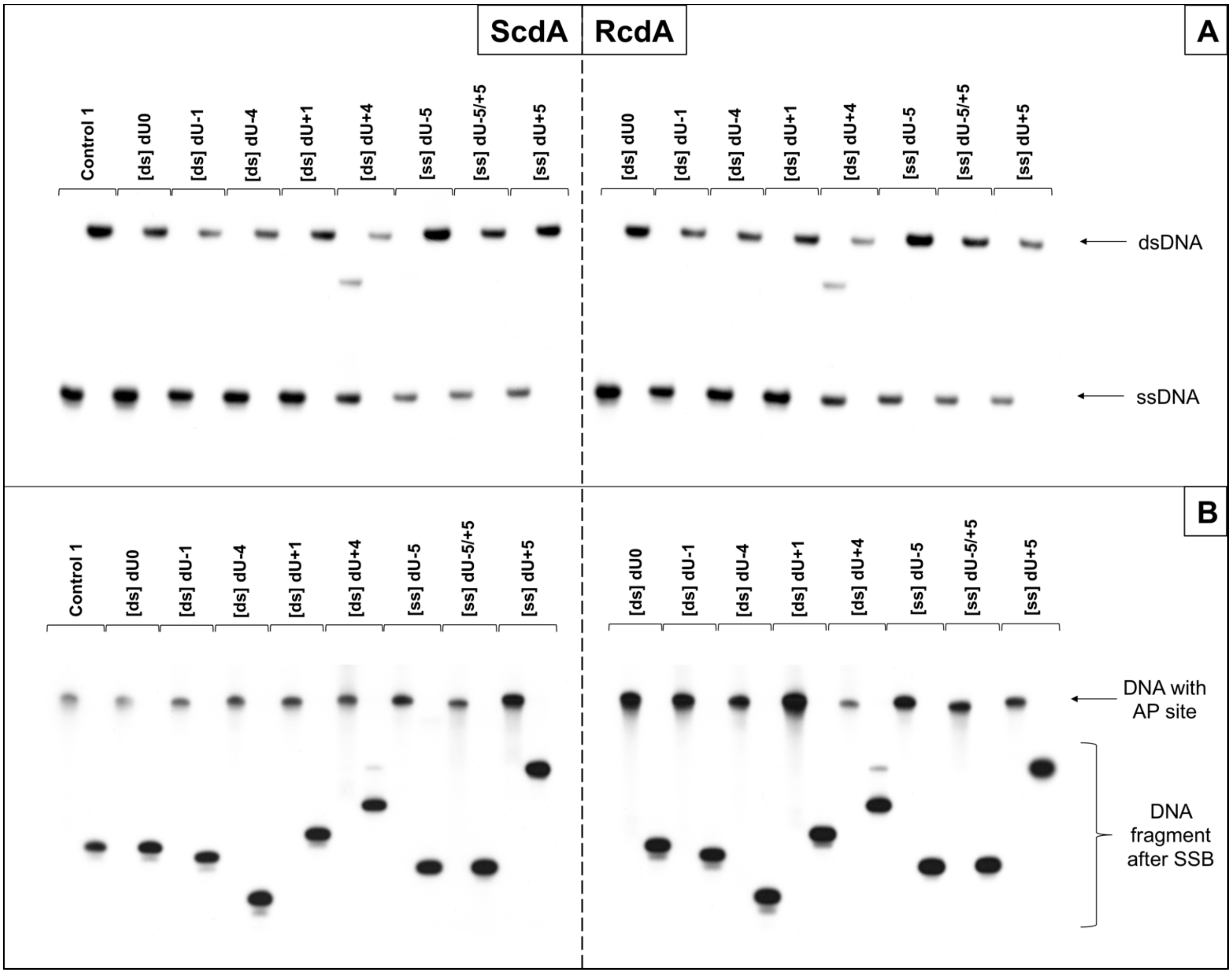

Figure S1. (A) Verification of efficient annealing of single-stranded (ssDNA) and double-stranded (dsDNA) oligonucleotides on the 15% native polyacrylamide gel. **(B)** Verification of AP sites' stability/purity and SSBs formation (after APE1 treatment) on the 15% denaturing polyacrylamide gel. [ds] – double-stranded lesions (located on the opposite DNA strands); [ss] – single-stranded lesions (located on the same DNA strand); SSB – single stranded break.

Table S1. The calculated vs. found masses of chosen substrate oligonucleotide strands used in the study.

Oligonucleotide	Calculated Mass	Found Mass
Control 1 – strand with dU	12,167.90	12,168.25
Control 1 – native strand	12,181.98	12,182.42
Mtx-ScdA	12,407.00	12,408.30
Mtx-RcdA	12,407.00	12,407.30
ScdA(-5)	12,165.90	12,166.501
ScdA(-5/+5)	12,166.89	12,167.2002
ScdA(+5)	12,180.90	12,181.541

A.

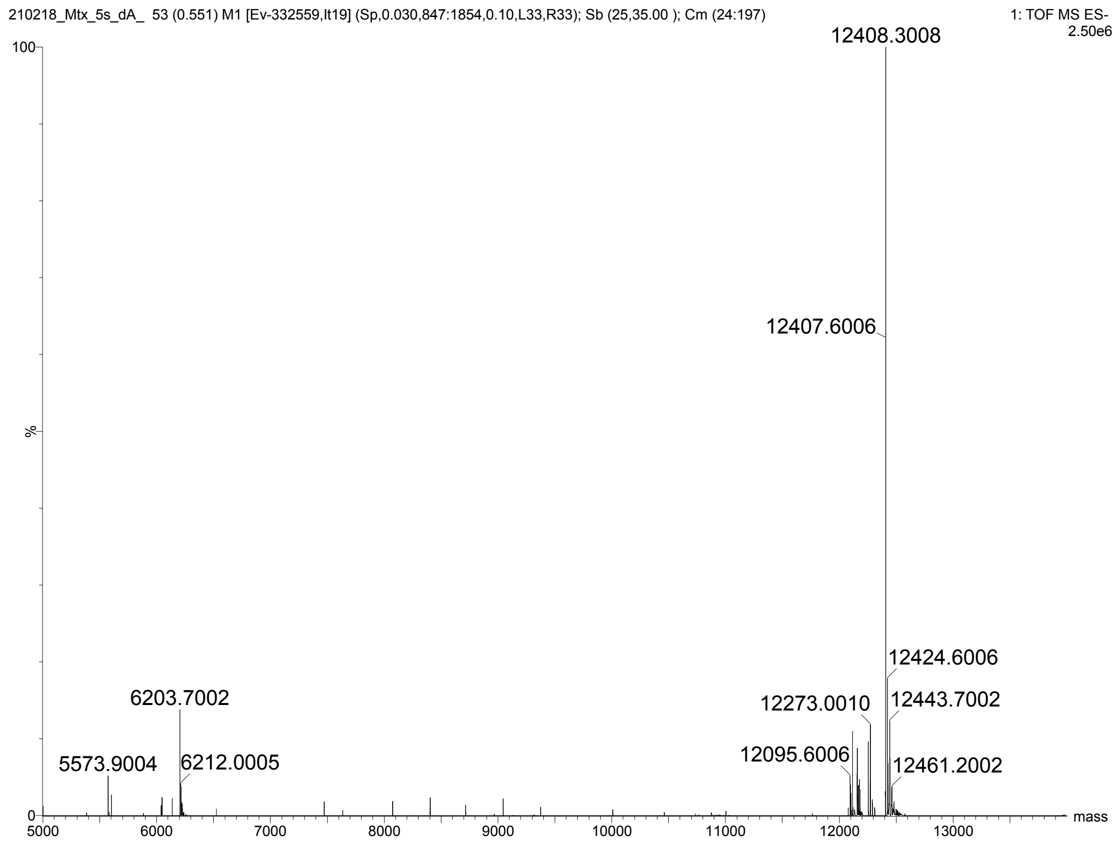
210218_Mtx_5s_dA_ 53 (0.551) M1 [Ev-332559,It19] (Sp,0.030,847:1854,0.10,L33,R33); Sb (25,35.00); Cm (24:197)
1: TOF MS ES-
2.50e6
12408.3008
12407.6006
12424.6006
12443.7002
12273.0010
12095.6006
12461.2002
6203.7002
5573.9004
6212.0005
100
%
0
5000
6000
7000
8000
9000
10000
11000
12000
13000
mass

B.

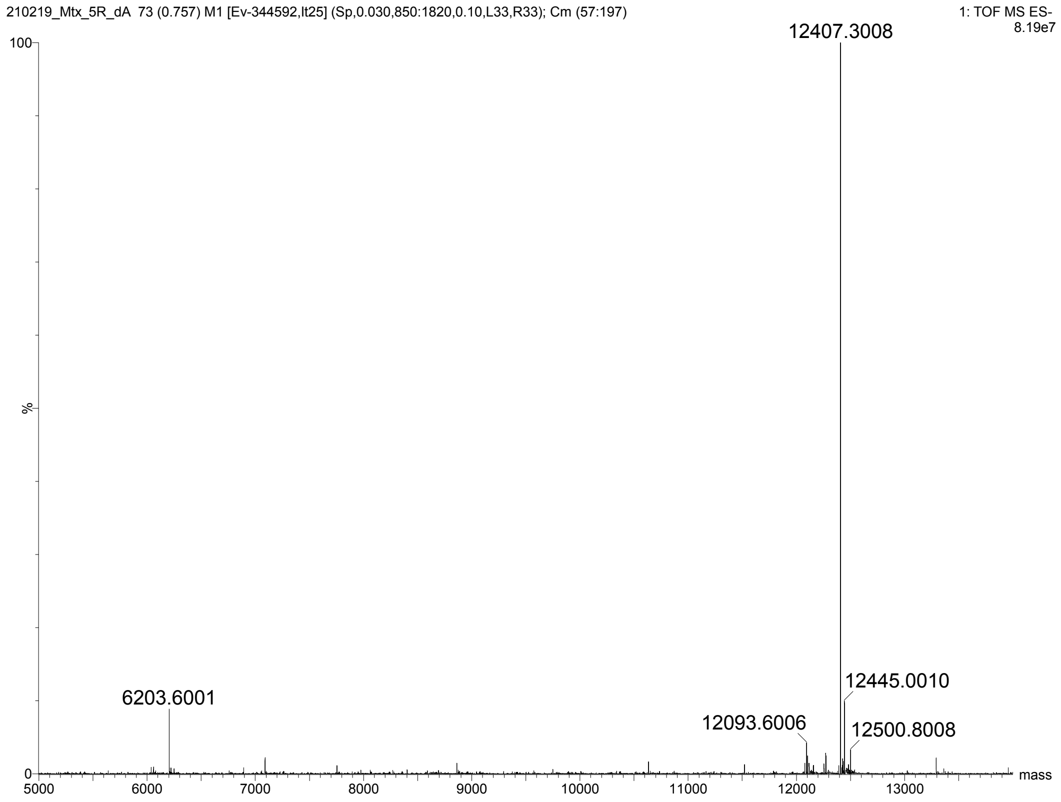
210219_Mtx_5R_dA 73 (0.757) M1 [Ev-344592,It25] (Sp,0.030,850:1820,0.10,L33,R33); Cm (57:197)
1: TOF MS ES-
8.19e7
12407.3008
12445.0010
12093.6006
12500.8008
6203.6001
100
%
0
5000
6000
7000
8000
9000
10000
11000
12000
13000
mass

C.

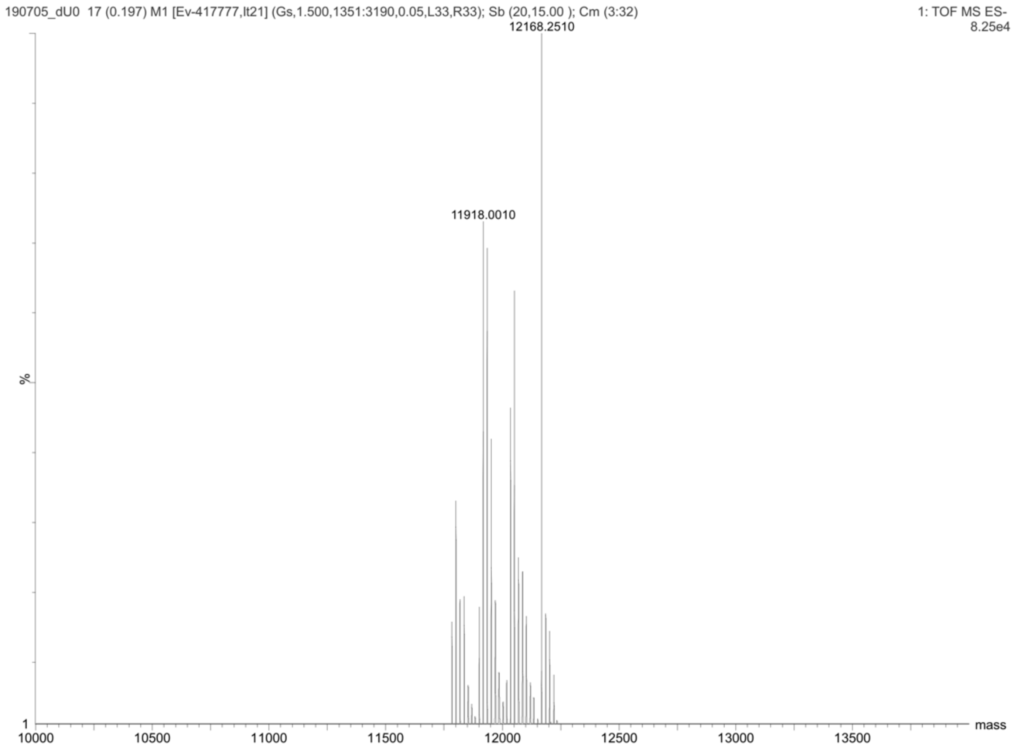
190705_dU0 17 (0.197) M1 [Ev-417777,It21] (Gs,1.500,1351:3190,0.05,L33,R33); Sb (20,15.00); Cm (3:32)
1: TOF MS ES-
8.25e4
12168.2510
11918.0010
%
1
10000
10500
11000
11500
12000
12500
13000
13500
mass

D.

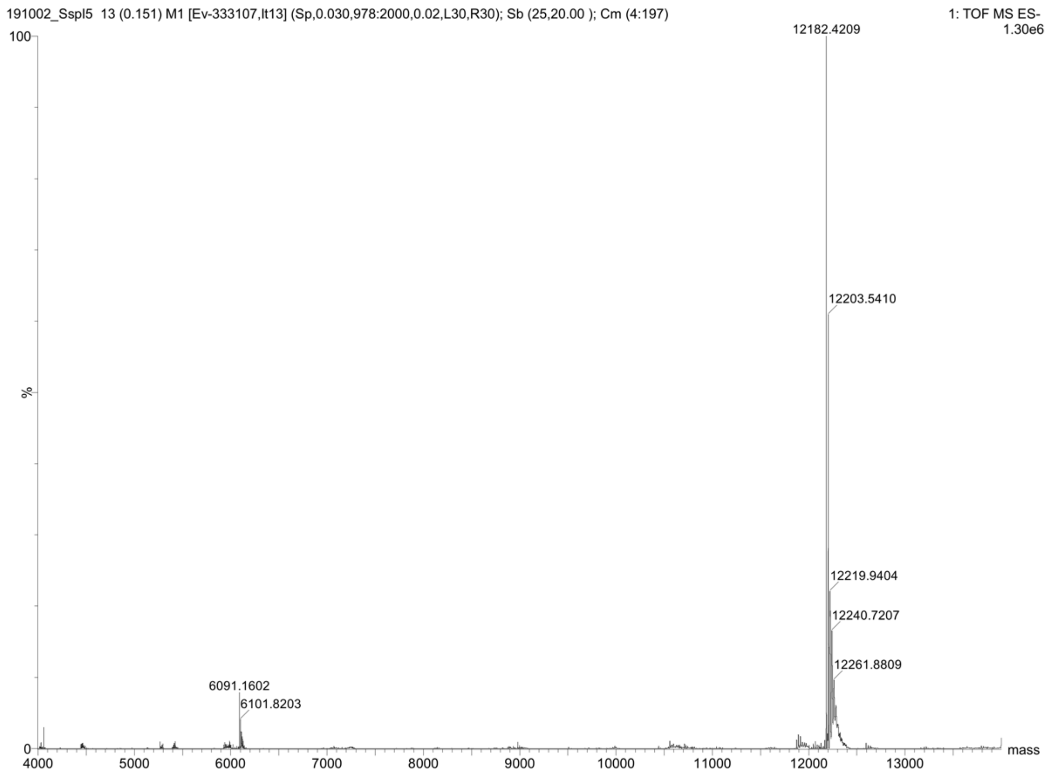
191002_Sspl5 13 (0.151) M1 [Ev-333107,It13] (Sp,0.030,978:2000,0.02,L30,R30); Sb (25,20.00); Cm (4:197)
1: TOF MS ES-
1.30e6
12182.4209
100
12203.5410
%
12219.9404
12240.7207
12261.8809
6091.1602
6101.8203
0
4000
5000
6000
7000
8000
9000
10000
11000
12000
13000
mass

E.

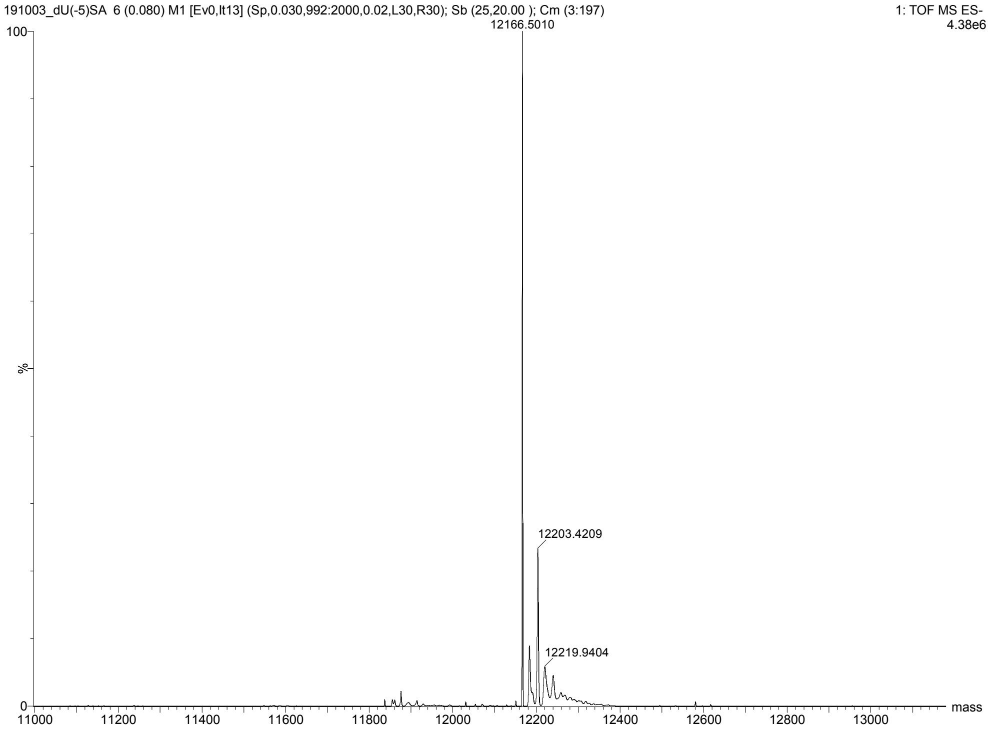
191003_dU(-5)SA 6 (0.080) M1 [Ev0,It13] (Sp,0.030,992:2000,0.02,L30,R30); Sb (25,20.00); Cm (3:197)
1: TOF MS ES-
4.38e6
12166.5010
12203.4209
12219.9404
100
%
0
mass
11000
11200
11400
11600
11800
12000
12200
12400
12600
12800
13000

F.

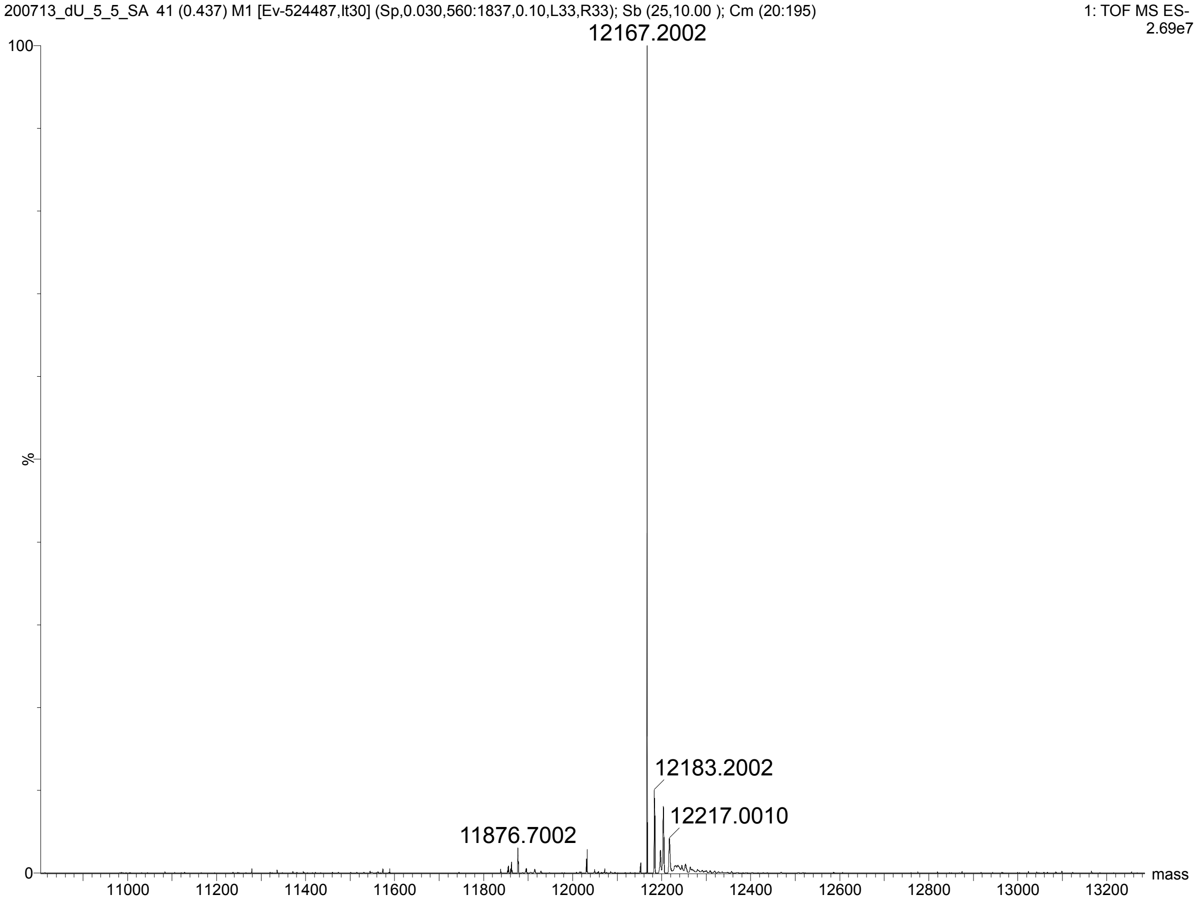
200713_dU_5_5_SA 41 (0.437) M1 [Ev-524487,It30] (Sp,0.030,560:1837,0.10,L33,R33); Sb (25,10.00); Cm (20:195)
1: TOF MS ES-
2.69e7
12167.2002
12183.2002
12217.0010
11876.7002
100
%
0
mass
11000
11200
11400
11600
11800
12000
12200
12400
12600
12800
13000
13200

G.

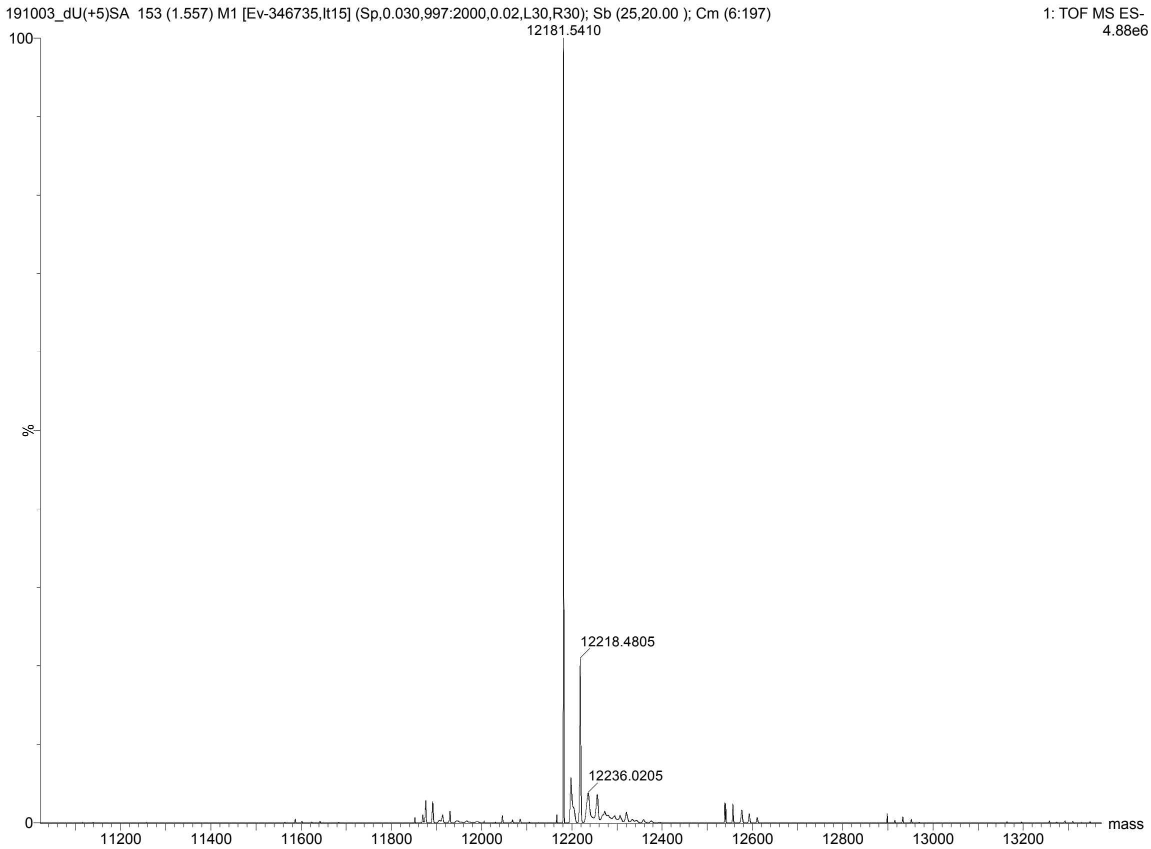

Figure S2. Results of mass spectrometry analysis of chosen substrate oligonucleotides containing cdA. **(A)** ssDNA with (5′S)-5′,8-cyclo-2′-deoxyadenosine (Mtx-ScdA); **(B)** ssDNA with (5′R)-5′,8-cyclo-2′-deoxyadenosine (Mtx-RcdA); **(C)** ssDNA with 2′-deoxyuridine (Control 1 – strand with dU); **(D)** native ssDNA (Control 1 – native strand); **(E)** ssDNA with (5′S)-5′,8-cyclo-2′-deoxyadenosine and 2′-deoxyuridine in the -5 position (ScdA(-5)); **(F)** ssDNA with (5′S)-5′,8-cyclo-2′-deoxyadenosine and two 2′-deoxyuridines in the -5 and +5 position (ScdA(-5/+5)); **(G)** ssDNA with (5′S)-5′,8-cyclo-2′-deoxyadenosine and 2′-deoxyuridine in the +5 position (ScdA(+5)).

A.

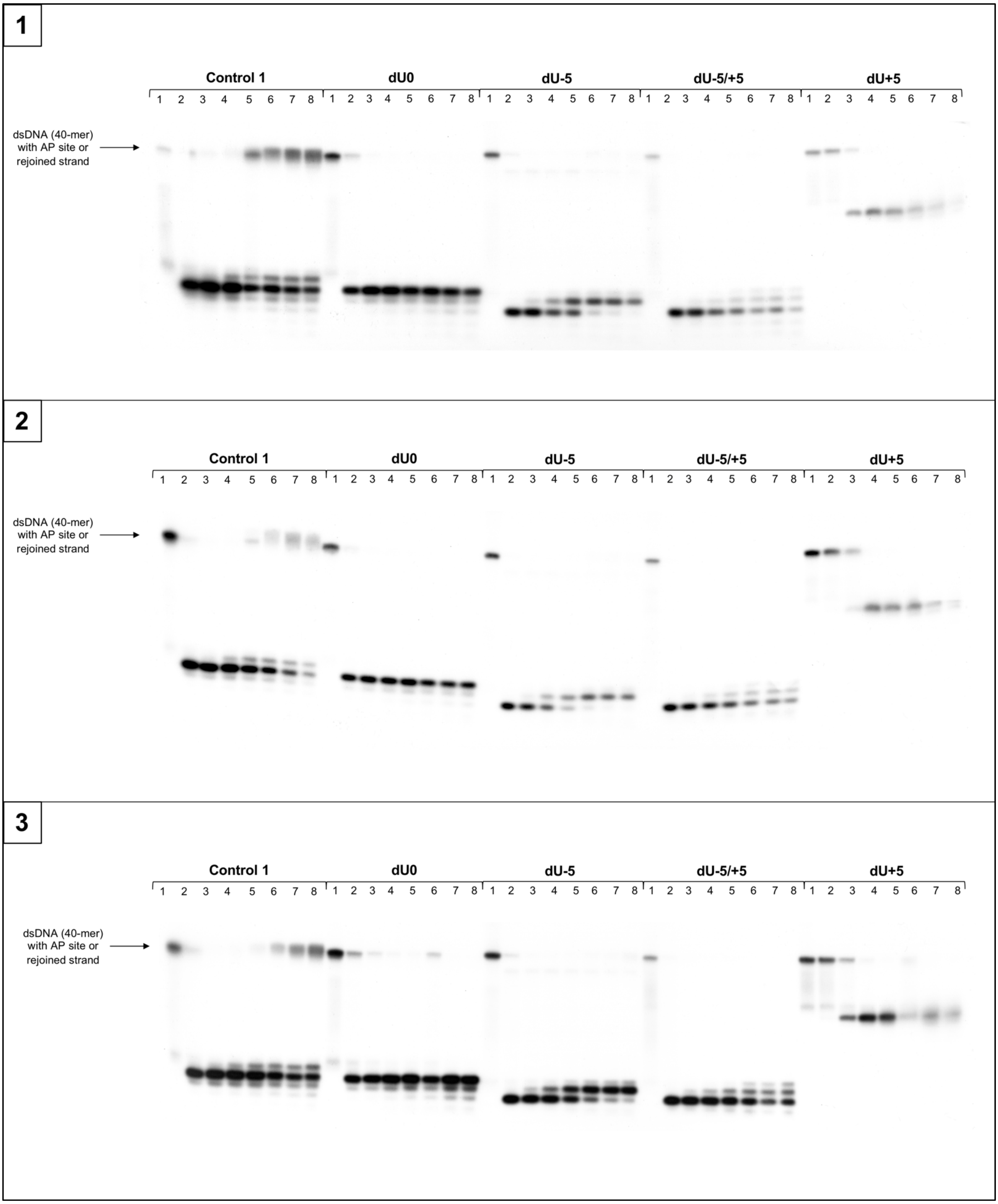
1
Control 1
dU0
dU-5
dU-5/+5
dU+5
1 2 3 4 5 6 7 8
dsDNA (40-mer) with AP site or rejoined strand
2
Control 1
dU0
dU-5
dU-5/+5
dU+5
1 2 3 4 5 6 7 8
dsDNA (40-mer) with AP site or rejoined strand
3
Control 1
dU0
dU-5
dU-5/+5
dU+5
1 2 3 4 5 6 7 8
dsDNA (40-mer) with AP site or rejoined strand

B.

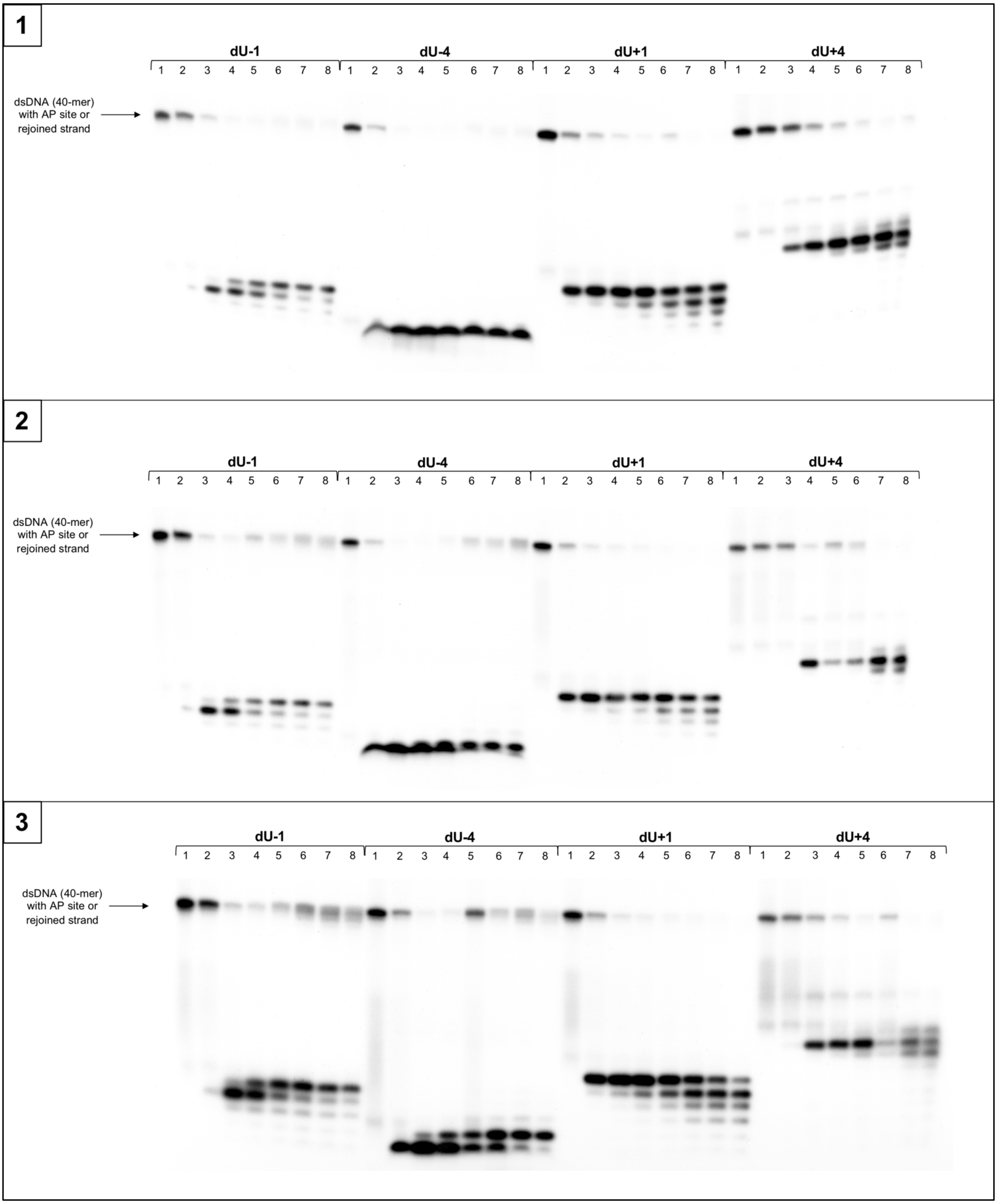

Figure S3. The autoradiograms presenting repair of **(A)** ss-CDL and **(B)** ds-CDL containing **ScdA** – XPC Nuclear Extract. Lanes correspond with different assay time: lane 1 - 0 min; lane 2 - 1 min; lane 3 – 5 min; lane 4 – 15 min; lane 5 – 30 min; lane 6 – 60 min; lane 7 – 90 min; lane 8 – 120 min. Three replications of the experiment are shown.

A.

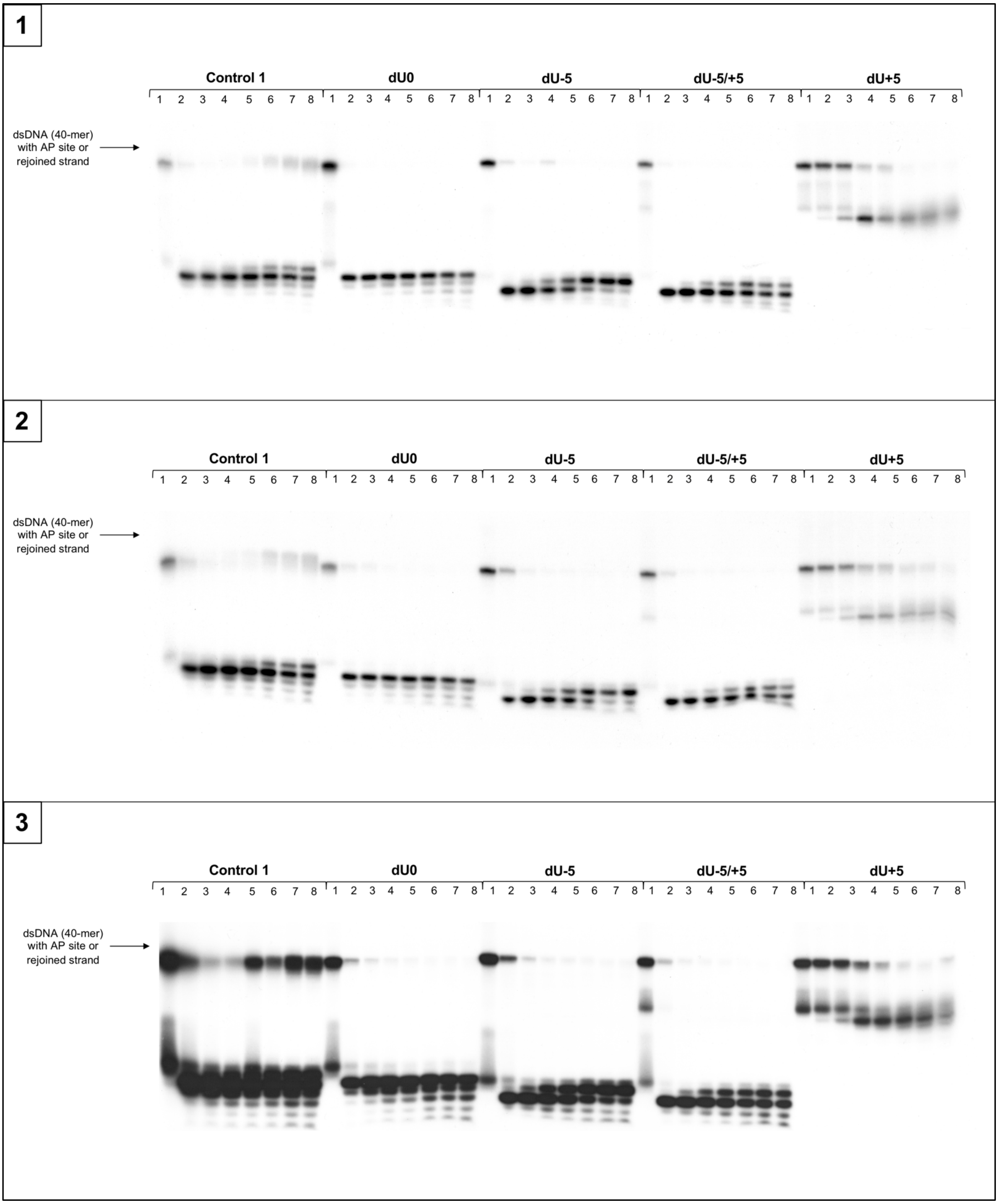
1
Control 1
dU0
dU-5
dU-5/+5
dU+5
1 2 3 4 5 6 7 8
dsDNA (40-mer) with AP site or rejoined strand
2
Control 1
dU0
dU-5
dU-5/+5
dU+5
1 2 3 4 5 6 7 8
dsDNA (40-mer) with AP site or rejoined strand
3
Control 1
dU0
dU-5
dU-5/+5
dU+5
1 2 3 4 5 6 7 8
dsDNA (40-mer) with AP site or rejoined strand

B.

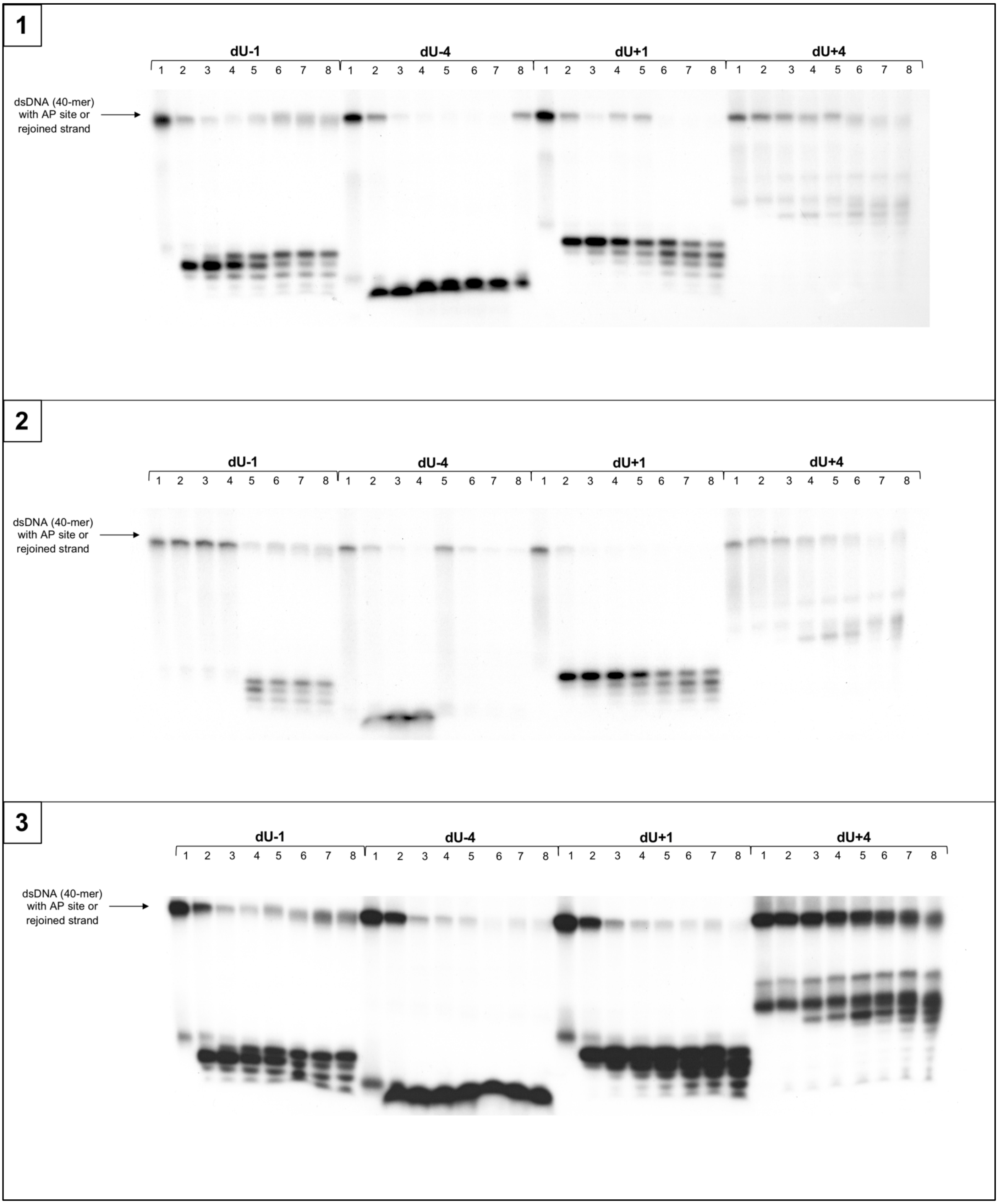

Figure S4. The autoradiograms presenting repair of **(A)** ss-CDL and **(B)** ds-CDL containing **RcdA** – XPC Nuclear Extract.
Lanes correspond with different assay time: lane 1 - 0 min; lane 2 - 1 min; lane 3 – 5 min; lane 4 – 15 min; lane 5 – 30 min; lane 6 – 60 min; lane 7 – 90 min; lane 8 – 120 min. Three replications of the experiment are shown.

A.

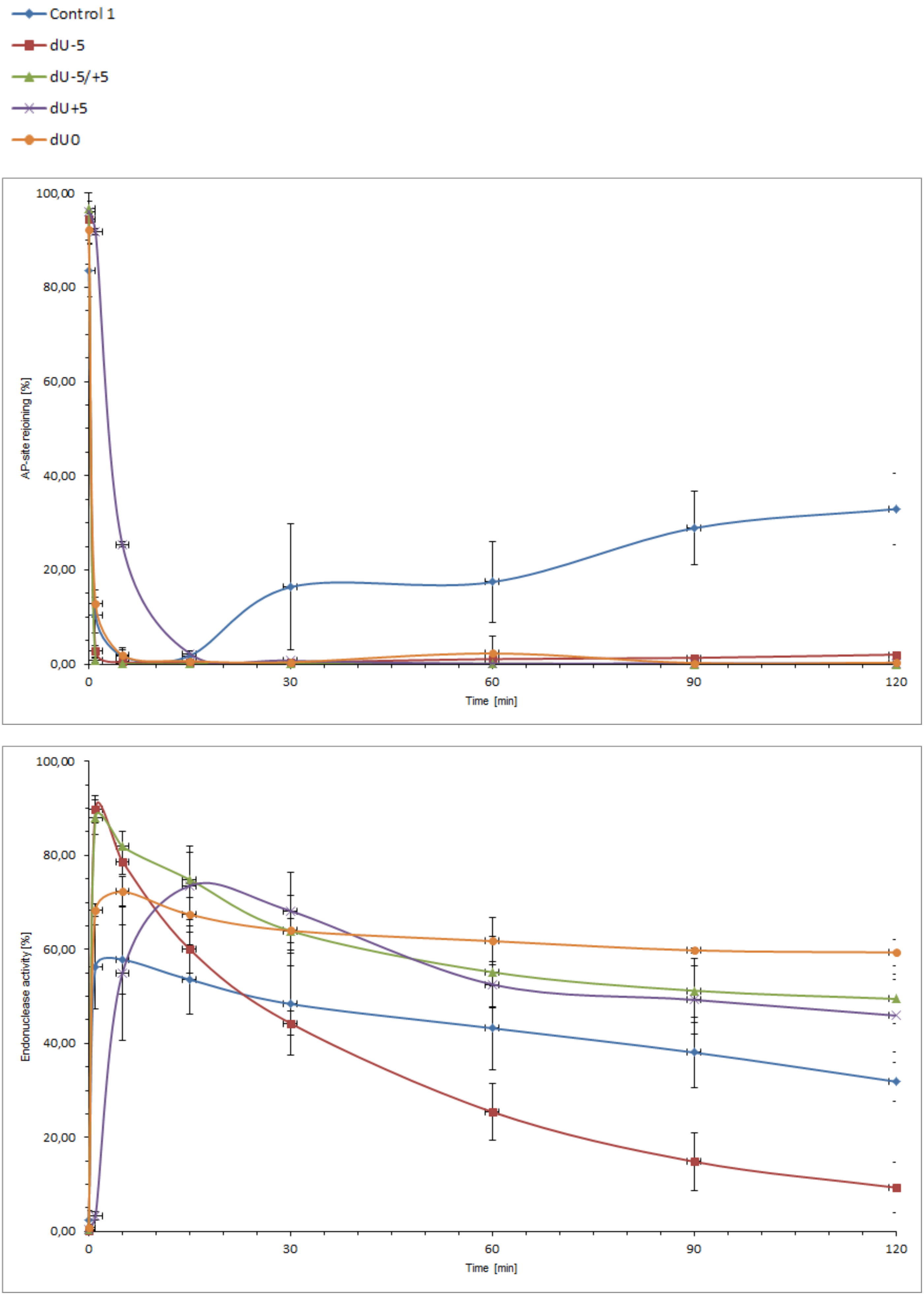

Control 1
dU-5
dU-5/+5
dU+5
dU0
AP-site rejoining [%]
100,00
80,00
60,00
40,00
20,00
0,00
0
30
60
90
120
Time [min]
Endonuclease activity [%]
100,00
80,00
60,00
40,00
20,00
0,00
0
30
60
90
120
Time [min]

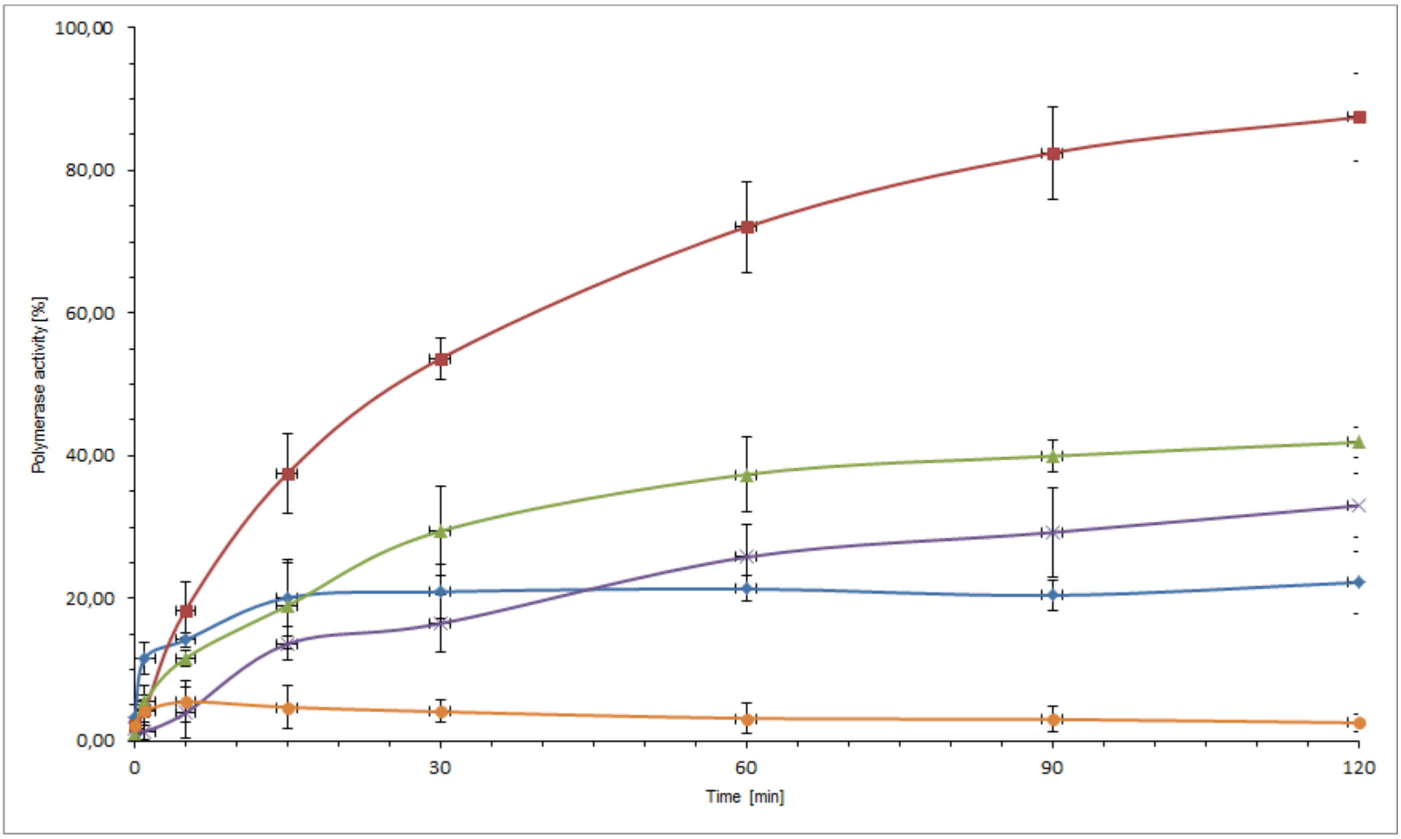
100,00
80,00
60,00
40,00
20,00
0,00
Polymerase activity [%]
0
30
60
90
120
Time [min]

B.

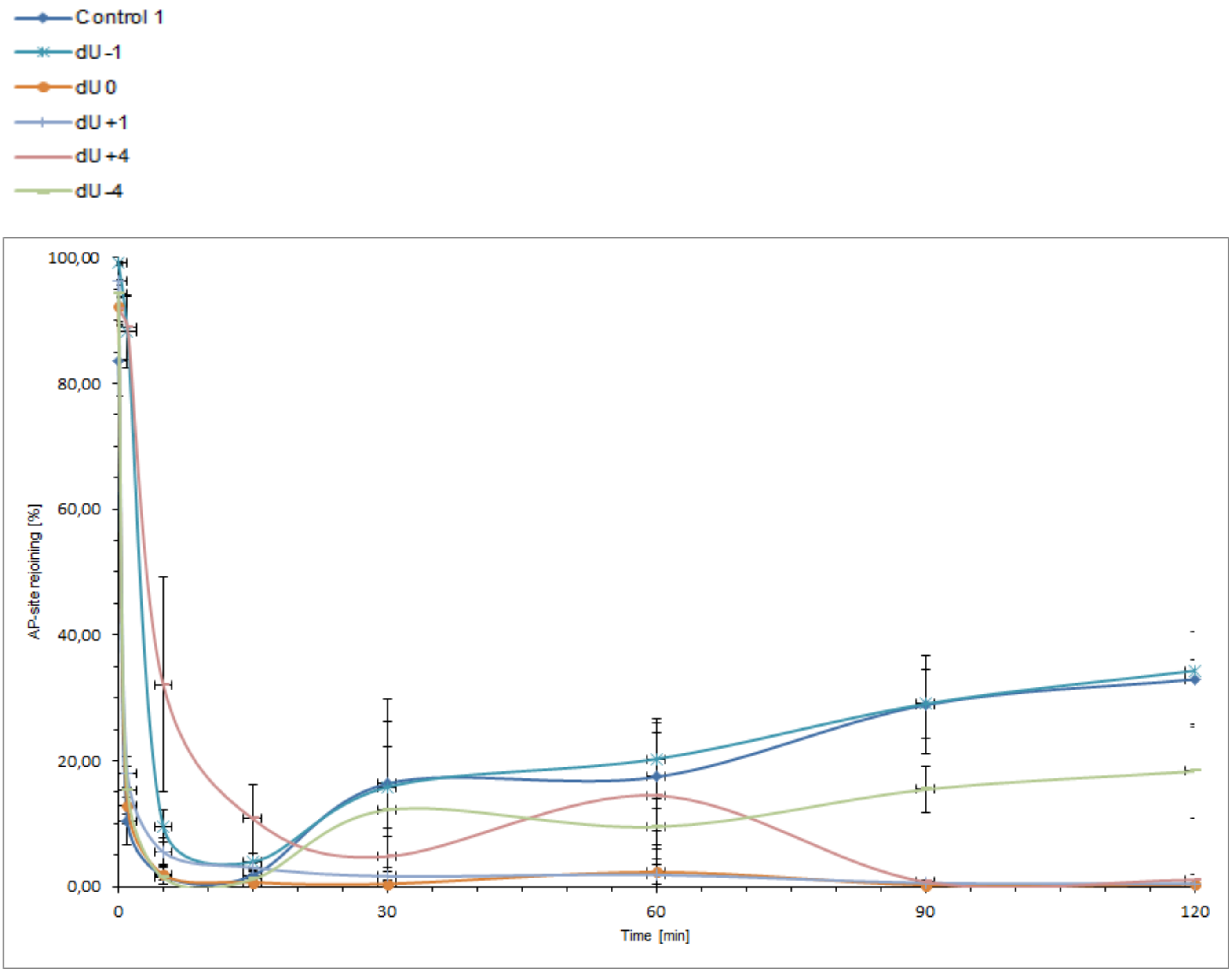
Control 1
dU -1
dU 0
dU +1
dU +4
dU -4
100,00
80,00
60,00
40,00
20,00
0,00
AP-site rejoining [%]
0
30
60
90
120
Time [min]

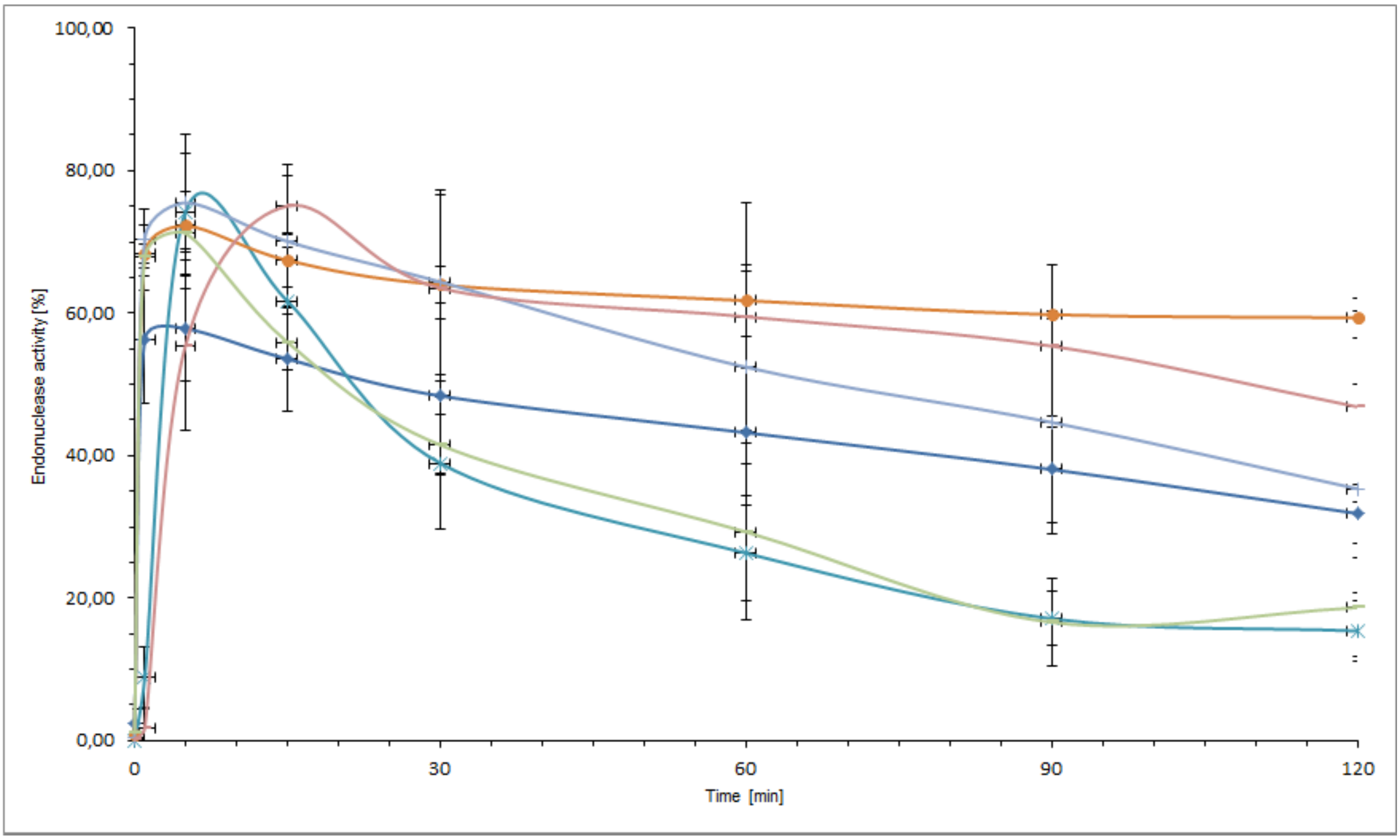

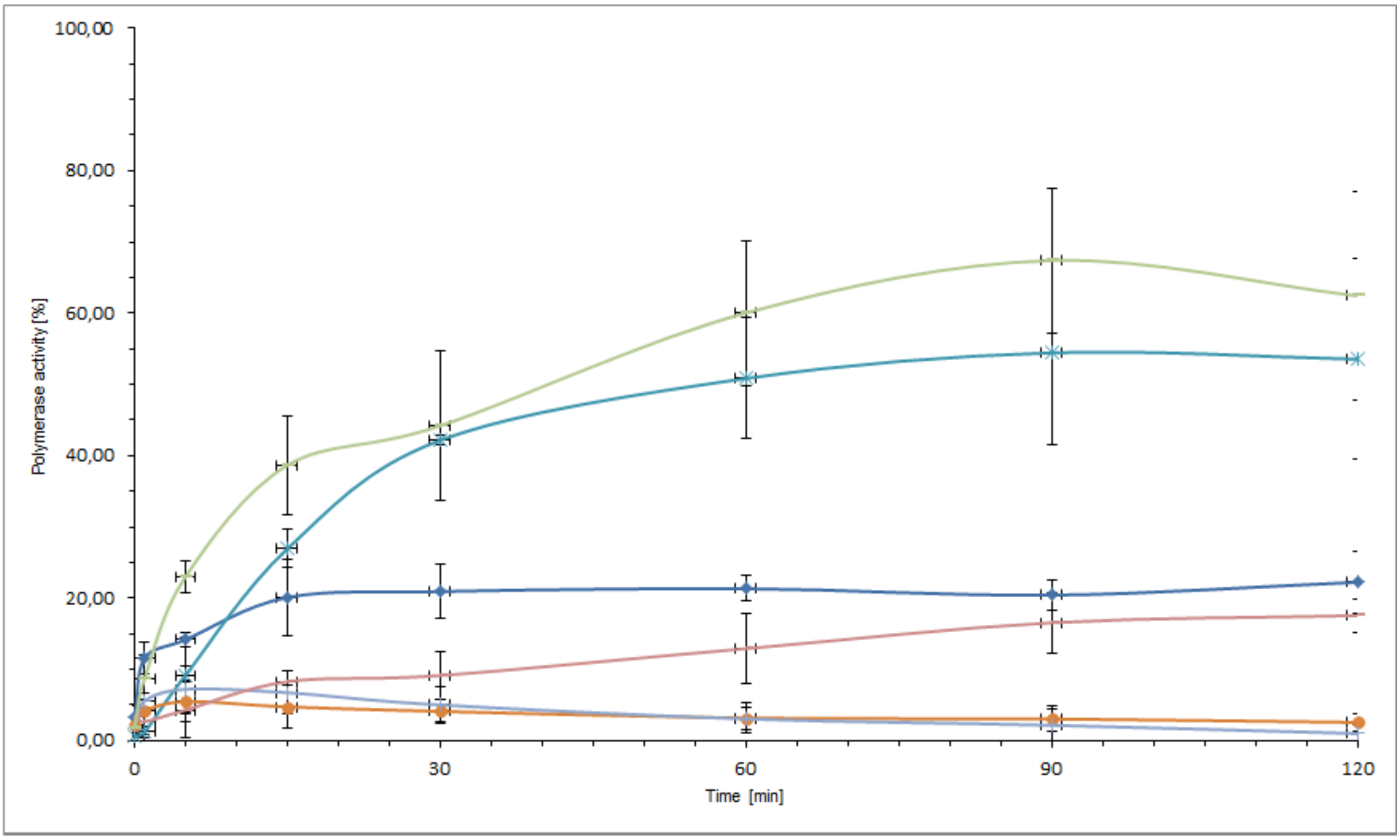

Figure S5. Graphical representation of DNA repair assays' results for **ScdA** – XPC Nuclear Extract. **(A)** ss-CDL, **(B)** ds-CDL

Table S2. Raw numerical data – Control 1 – XPC Nuclear Extract. * rejected values

		Time [min]							
		0	1	5	15	30	60	90	120
Strand	Data set	**AP site rejoining [%]**							
Control 1	1.	85,02	5,49	1,23	1,20	3,40	8,83	16,75	21,67
	2.	75,90	13,68	4,06	3,27	4,23	9,54	12,94*	17,60*
	3.	67,09*	28,76*	12,53*	12,43*	28,18	23,19	30,46	29,81
	4.	85,96	12,80	1,41	2,18	32,51	29,20	35,99	39,58
	5.	80,66	13,17	1,19	1,55	13,60	22,32	35,02	40,21
	6.	90,74	7,04	0,64	0,82	2,36*	11,54	26,22	33,53
	Avg	83,65	10,44	1,70	1,80	16,39	17,44	28,89	32,96
	SD	5,62	3,86	1,35	0,96	13,45	8,56	7,83	7,65

		Time [min]							
		0	1	5	15	30	60	90	120
Strand	Data set	**Endonuclease activity [%]**							
Control 1	1.	1,40	60,30	60,14	56,12	53,75	46,34	40,28	31,91
	2.	5,75	55,95	59,50	57,26	57,07	48,12	45,53	36,20
	3.	16,12*	24,92*	27,83*	27,65*	22,10*	24,66*	20,69*	20,99*
	4.	0,49	53,91	54,84	48,48	36,20	33,51	30,23	26,88
	5.	2,83	43,46	47,43	43,79	37,20	34,31	29,93	28,78
	6.	1,32	67,99	67,25	62,24	57,81	53,91	44,39	35,66
	Avg	2,36	56,32	57,83	53,58	48,40	43,24	38,07	31,89
	SD	2,07	8,99	7,31	7,36	10,80	8,97	7,56	4,11

		Time [min]							
		0	1	5	15	30	60	90	120
Strand	Data set	**Polymerase activity [%]**							
Control 1	1.	5,64	12,47	13,04	16,47	18,49	22,57	21,81	27,80
	2.	3,42	12,35	12,88	13,84	15,81	19,40	23,31	25,67
	3.	11,20*	10,08	14,43	16,34	20,69	21,62	18,14	18,68
	4.	1,64	7,96	14,65	26,20	19,81	20,89	19,65	20,47
	5.	4,15	13,46	15,43	26,03	26,21	23,97	18,93	19,70
	6.	1,86	13,40	14,67	21,74	24,59	19,60	18,47	17,57
	Avg	3,34	11,62	14,18	20,11	20,93	21,34	20,44	22,24
	SD	1,66	2,17	1,01	5,32	3,87	1,76	2,06	4,30

Table S3. Raw numerical data – ScdA – XPC Nuclear Extract.

ScdA		Time [min]							
		0	1	5	15	30	60	90	120
Strand	Data set	**AP site rejoining [%]**							
dU0	1.	91,10	11,48	0,71	0,26	0,15	0,10	0,02	0,09
	2.	95,97	10,96	1,25	0,59	0,36	0,27	0,29	0,50
	3.	89,98	16,10	3,75	1,18	0,81	6,51	0,44	0,43
	Avg	92,35	12,85	1,90	0,68	0,44	2,29	0,25	0,34
	SD	3,19	2,83	1,62	0,46	0,34	3,65	0,21	0,22
dU-5	1.	93,19	2,40	0,17	0,12	0,20	1,12	0,98	1,38
	2.	97,13	1,86	0,45	0,25	0,40	1,22	1,95	2,36
	3.	92,90	4,18	0,89	0,61	0,63	0,94	1,14	2,39
	Avg	94,41	2,81	0,50	0,33	0,41	1,09	1,36	2,04
	SD	2,36	1,22	0,37	0,26	0,21	0,14	0,52	0,57
dU -5/+5	1.	99,78	0,37	0,01	0,01	0,11	0,37	0,00	0,00
	2.	97,30	0,90	0,03	0,00	0,00	0,00	0,01	0,00
	3.	93,33	1,25	0,29	0,11	0,09	0,03	0,02	0,03
	Avg	96,80	0,84	0,11	0,04	0,07	0,14	0,01	0,01
	SD	3,25	0,44	0,16	0,06	0,06	0,21	0,01	0,02
dU+5	1.	96,77	91,42	24,99	1,99	0,39	0,08	0,05	0,04
	2.	97,78	92,53	54,80*	2,81	1,15	0,19	0,12	0,12
	3.	93,82	91,54	25,79	1,98	0,71	13,04*	0,47	0,32
	Avg	96,12	91,83	25,39	2,26	0,75	0,13	0,21	0,16
	SD	2,06	0,61	0,56	0,48	0,39	0,07	0,22	0,14
dU-1	1.	99,26	81,66	7,73	5,17	11,25	24,78	32,89	35,50
	2.	99,17	91,42	12,52	2,55	2,60*	4,39*	7,15*	4,69*
	3.	98,95	91,41	8,58	4,16	20,43	15,82	25,23	33,18
	Avg	99,13	88,16	9,61	3,96	15,84	20,30	29,06	34,34
	SD	0,16	5,63	2,56	1,32	6,49	6,34	5,42	1,64
dU-4	1.	93,89	19,34	1,83	2,12	22,16	7,47	18,05	12,98
	2.	92,13	14,55	1,07	0,47	0,67*	2,10*	2,63*	3,50*
	3.	96,59	12,05	1,22	0,89	2,23	11,50	12,82	23,69
	Avg	94,20	15,31	1,38	1,16	12,20	9,49	15,43	18,33
	SD	2,25	3,70	0,40	0,86	14,10	2,85	3,69	7,57
dU+1	1.	99,21	16,28	4,00	2,46	1,56	1,07	0,63	0,51
	2.	94,67	21,02	8,11	2,92	1,10	3,68	0,36	0,29
	3.	95,00	16,50	4,39	3,65	2,32	0,92	0,72	0,57
	Avg	96,29	17,93	5,50	3,01	1,66	1,89	0,57	0,46
	SD	2,53	2,67	2,26	0,60	0,62	1,55	0,19	0,15
dU+4	1.	91,29	83,09	20,01	8,90	2,55	19,29	0,77	0,78
	2.	90,16	91,06	44,23	16,83	7,05	2,99	0,94	2,02
	3.	93,75	92,78	93,79*	6,53	43,48*	21,18	0,57	0,46
	Avg	91,73	88,98	32,12	10,76	4,80	14,48	0,76	1,09
	SD	1,84	5,17	17,12	5,40	3,18	10,00	0,18	0,82

* rejected values

ScdA		Time [min]							
		0	1	5	15	30	60	90	120
Strand	Data set	**Endonuclease activity [%]**							
dU0	1.	1,69	69,81	70,71	65,06	63,99	58,22	59,32	62,11
	2.	0,34	67,26	70,19	65,52	61,31	59,67	60,41	56,59
	3.	0,57	67,88	75,95	71,69	66,66	67,41	59,66	59,39
	Avg	0,87	68,31	72,28	67,43	63,98	61,77	59,80	59,36
	SD	0,72	1,33	3,19	3,70	2,67	4,94	0,56	2,76
dU-5	1.	0,00	92,32	79,89	59,01	43,35	20,68	11,31	4,84
	2.	0,00	90,44	75,63	55,58	42,37	23,49	11,32	8,05
	3.	0,55	86,57	80,64	65,53	47,14	32,11	21,96	15,15
	Avg	0,18	89,78	78,72	60,04	44,29	25,42	14,86	9,35
	SD	0,32	2,93	2,70	5,05	2,52	5,96	6,15	5,28
dU -5/+5	1.	0,00	91,18	83,16	78,48	64,48	55,07	51,04	51,13
	2.	0,00	83,94	78,30	66,59	56,16	47,71	44,51	43,60
	3.	0,97	89,00	84,16	79,47	71,18	62,71	58,11	53,68
	Avg	0,32	88,04	81,87	74,85	63,94	55,16	51,22	49,47
	SD	0,56	3,72	3,13	7,17	7,52	7,50	6,80	5,24
dU+5	1.	0,13	3,80	61,68	67,31	59,48	48,91	40,99	37,48
	2.	0,60	3,98	38,56	71,92	69,22	57,93	52,69	47,71
	3.	0,42	2,20	64,60	81,39	75,82	50,83	54,11	52,58
	Avg	0,38	3,33	54,95	73,54	68,17	52,56	49,26	45,93
	SD	0,24	0,98	14,26	7,18	8,22	4,75	7,20	7,70
dU-1	1.	0,00	13,78	61,83	50,66	34,87	22,83	16,78	14,98
	2.	0,02	6,69	79,82	67,52	49,63	34,02	21,06	19,84
	3.	0,01	6,10	81,07	66,91	32,35	22,13	13,52	11,38
	Avg	0,01	8,86	74,24	61,69	38,95	26,33	17,12	15,40
	SD	0,01	4,27	10,76	9,56	9,34	6,67	3,78	4,25
dU-4	1.	0,59	66,92	66,70	58,76	38,46	38,12	21,03	13,76
	2.	1,87	63,80	77,73	76,35*	80,49*	83,75*	87,40*	83,36*
	3.	0,84	72,68	69,25	53,11	44,54	20,49	12,19	23,62
	Avg	1,10	67,80	71,23	55,94	41,50	29,30	16,61	18,69
	SD	0,68	4,50	5,78	4,00	4,31	12,46	6,25	6,97
dU+1	1.	0,00	70,90	70,60	60,77	52,78	39,42	29,72	20,11
	2.	0,70	66,03	72,46	70,06	62,03	51,42	43,47	36,64
	3.	0,49	74,22	83,50	79,39	78,38	66,44	60,85	49,31
	Avg	0,40	70,38	75,52	70,07	64,40	52,42	44,68	35,35
	SD	0,36	4,12	6,97	9,31	12,96	13,54	15,60	14,64
dU+4	1.	0,00	4,80	63,94	70,13	67,13	41,34	42,17	31,31
	2.	0,11	0,32	46,98	73,55	74,46	71,56	61,56	54,29
	3.	0,25	0,34	0,58*	81,47	49,00	65,61	62,43	54,97
	Avg	0,12	1,82	55,46	75,05	63,53	59,50	55,39	46,86
	SD	0,13	2,58	11,99	5,82	13,11	16,01	11,45	13,47

* rejected values

ScdA		Time [min]							
		0	1	5	15	30	60	90	120
Strand	Data set	**Polymerase activity [%]**							
dU0	1.	2,86	4,69	6,16	4,23	3,69	3,03	2,09	1,93
	2.	0,94	2,45	2,27	2,14	2,80	1,22	2,02	1,74
	3.	2,84	5,40	8,06	7,92	5,87	5,35	5,06	4,05
	Avg	2,22	4,18	5,50	4,77	4,12	3,20	3,06	2,57
	SD	1,10	1,54	2,95	2,92	1,58	2,07	1,74	1,28
dU-5	1.	1,91	2,17	17,21	38,97	54,40	77,25	86,81	92,91
	2.	1,06	4,42	22,64	42,33	56,12	73,95	85,60	88,80
	3.	3,56	6,46	15,17	31,35	50,36	65,01	75,05	80,71
	Avg	2,18	4,35	18,34	37,55	53,63	72,07	82,49	87,47
	SD	1,27	2,15	3,86	5,62	2,96	6,33	6,47	6,21
dU -5/+5	1.	0,21	4,50	12,19	18,27	29,95	38,26	37,86	39,43
	2.	0,52	8,01	12,36	25,43	35,44	42,11	42,41	43,60
	3.	2,56	4,08	10,31	13,28	22,91	31,77	39,59	42,62
	Avg	1,10	5,53	11,62	18,99	29,43	37,38	39,95	41,88
	SD	1,27	2,16	1,14	6,10	6,28	5,23	2,30	2,18
dU+5	1.	2,35	1,78	6,90	15,57	21,01	27,84	32,89	36,18
	2.	0,00	0,00	0,00	14,30	13,79	20,44	22,02	28,02
	3.	2,62	2,31	4,93	11,01	14,67	29,08	32,84	34,86
	Avg	1,66	1,36	3,95	13,63	16,49	25,79	29,25	33,02
	SD	1,44	1,21	3,55	2,35	3,94	4,67	6,26	4,38
dU-1	1.	0,74	2,14	15,29	29,92	41,56	41,04	40,96	41,42
	2.	0,54	0,84	5,55	26,08	42,80	55,81	66,70	69,01
	3.	0,37	0,79	6,71	24,80	42,14	55,69	55,69	50,18
	Avg	0,55	1,26	9,18	26,93	42,17	50,85	54,45	53,54
	SD	0,18	0,76	5,32	2,67	0,62	8,49	12,91	14,10
dU-4	1.	1,48	6,42	21,47	33,76	36,81	52,85	60,23	72,82
	2.	5,41*	14,30*	7,69*	12,25*	11,92*	10,58*	6,91*	10,73*
	3.	2,18	11,14	24,56	43,55	51,57	67,24	74,57	52,04
	Avg	1,83	8,78	23,01	38,65	44,19	60,05	67,40	62,43
	SD	0,50	3,33	2,19	6,93	10,44	10,17	10,14	14,69
dU+1	1.	0,79	5,43	7,41	5,57	3,09	1,39	0,27	0,00
	2.	1,78	6,69	10,28	10,12	7,93	4,49	4,52	2,33
	3.	2,72	4,65	3,97	4,54	4,05	3,30	1,72	0,68
	Avg	1,76	5,59	7,22	6,74	5,02	3,06	2,17	1,00
	SD	0,96	1,03	3,16	2,97	2,56	1,56	2,16	1,20
dU+4	1.	4,76	4,40	8,89	9,14	12,63	18,43	21,32	20,13
	2.	1,66	0,93	2,52	6,49	8,80	11,54	15,15	17,18
	3.	3,61	2,72	1,53	9,25	6,08	8,81	13,22	15,41
	Avg	3,34	2,68	4,31	8,29	9,17	12,93	16,56	17,57
	SD	1,57	1,73	3,99	1,56	3,29	4,96	4,23	2,38

* rejected values

A.

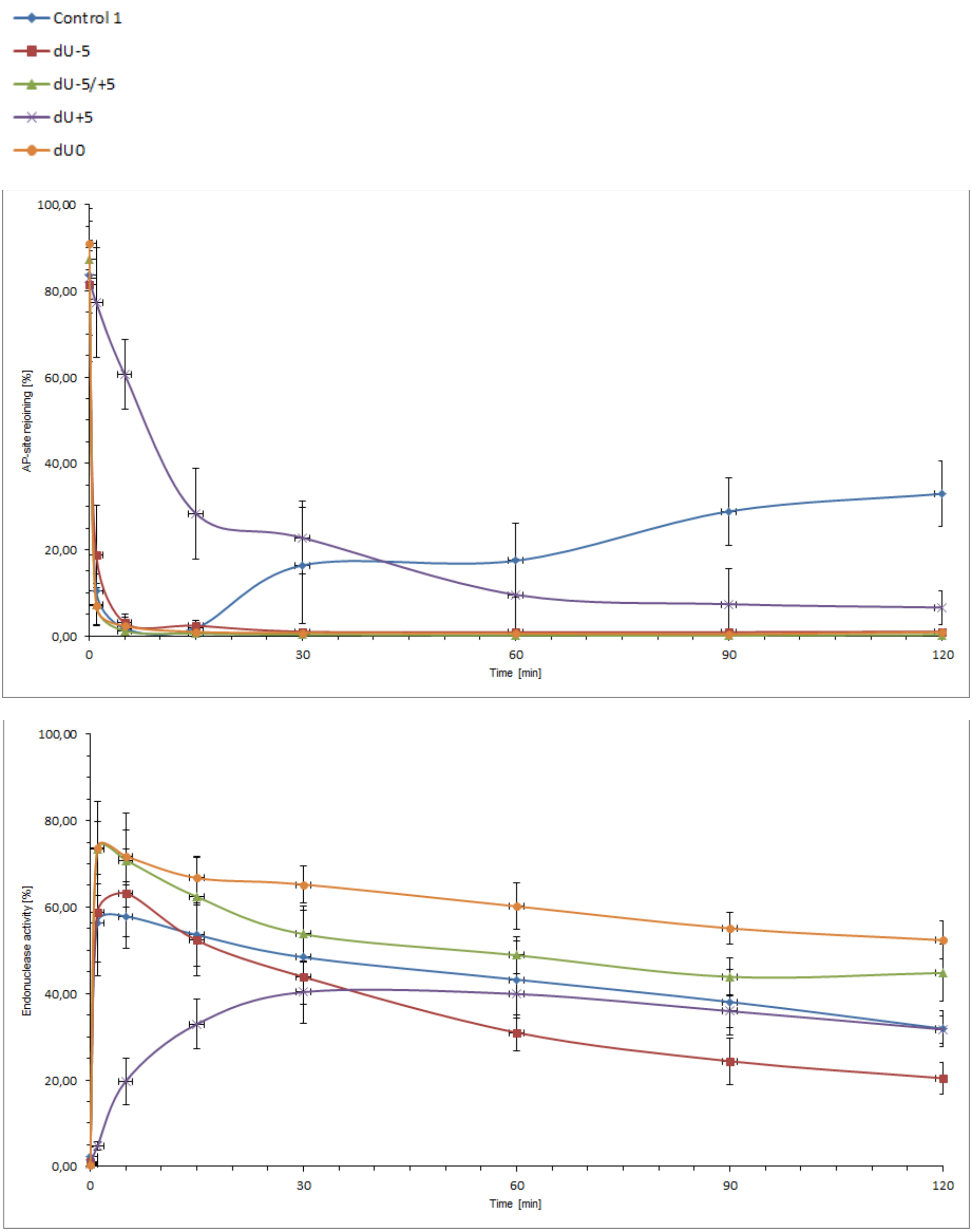
Control 1
dU-5
dU-5/+5
dU+5
dU0
100,00
80,00
60,00
40,00
20,00
0,00
AP-site rejoining [%]
0
30
60
90
120
Time [min]
100,00
80,00
60,00
40,00
20,00
0,00
Endonuclease activity [%]
0
30
60
90
120
Time [min]

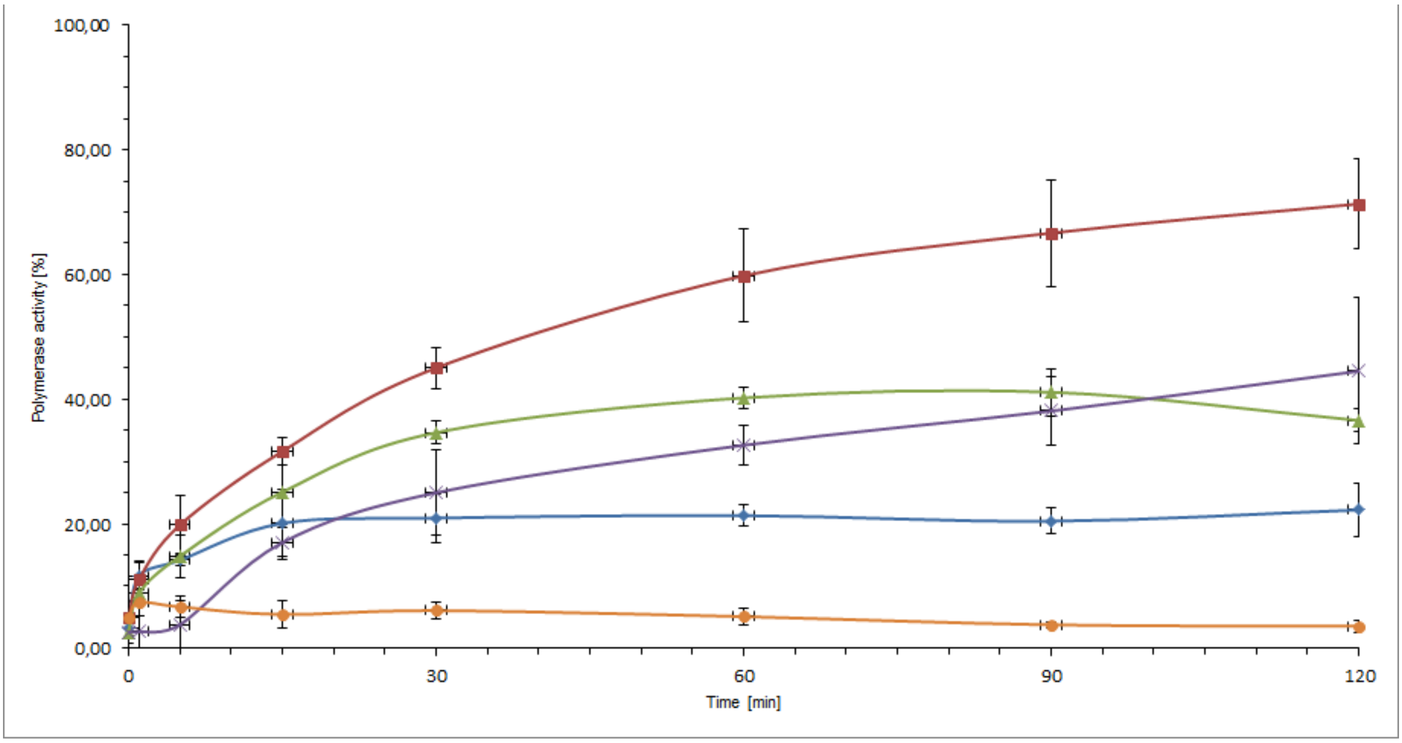
100,00
80,00
60,00
40,00
20,00
0,00
Polymerase activity [%]
0
30
60
90
120
Time [min]

B.

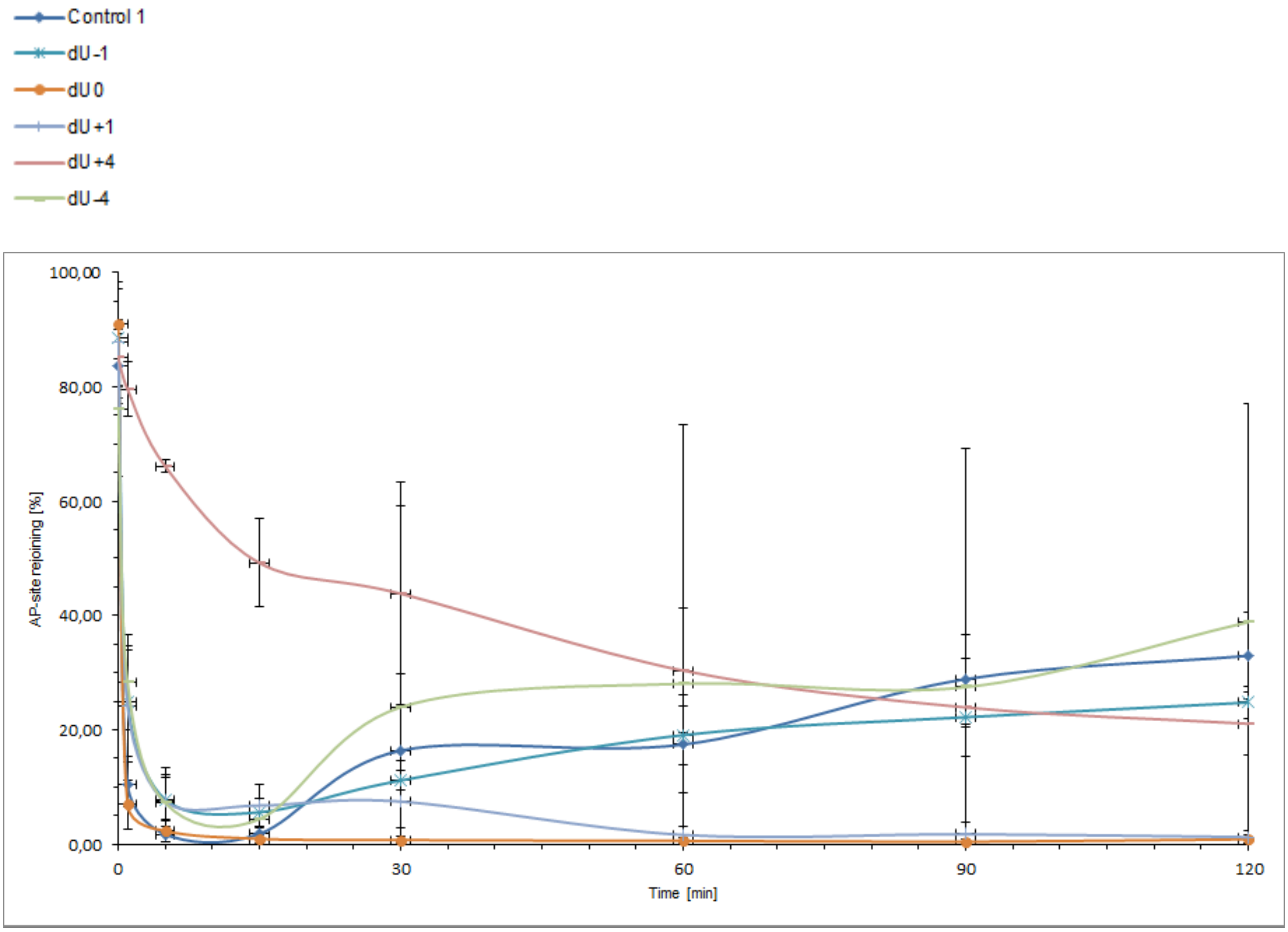
Control 1
dU -1
dU 0
dU +1
dU +4
dU -4
100,00
80,00
60,00
40,00
20,00
0,00
AP-site rejoining [%]
0
30
60
90
120
Time [min]

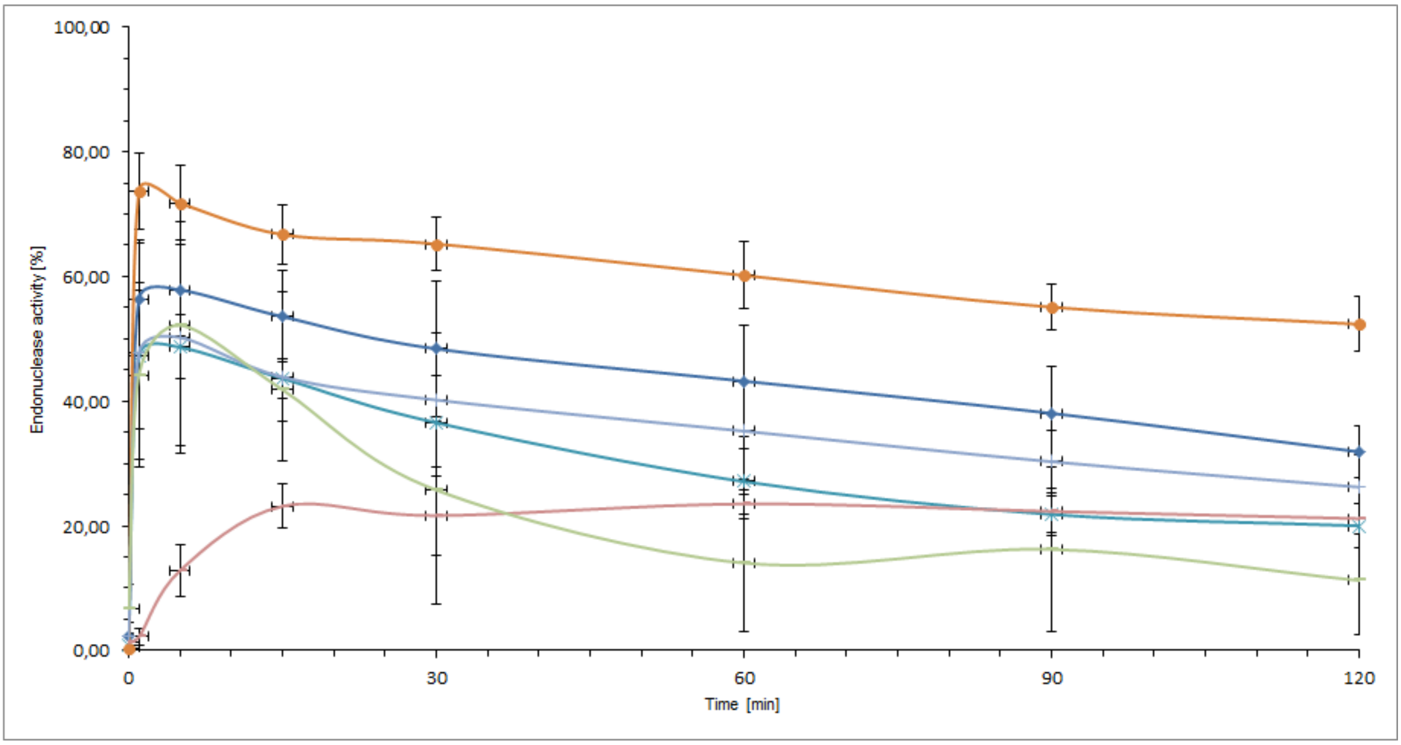

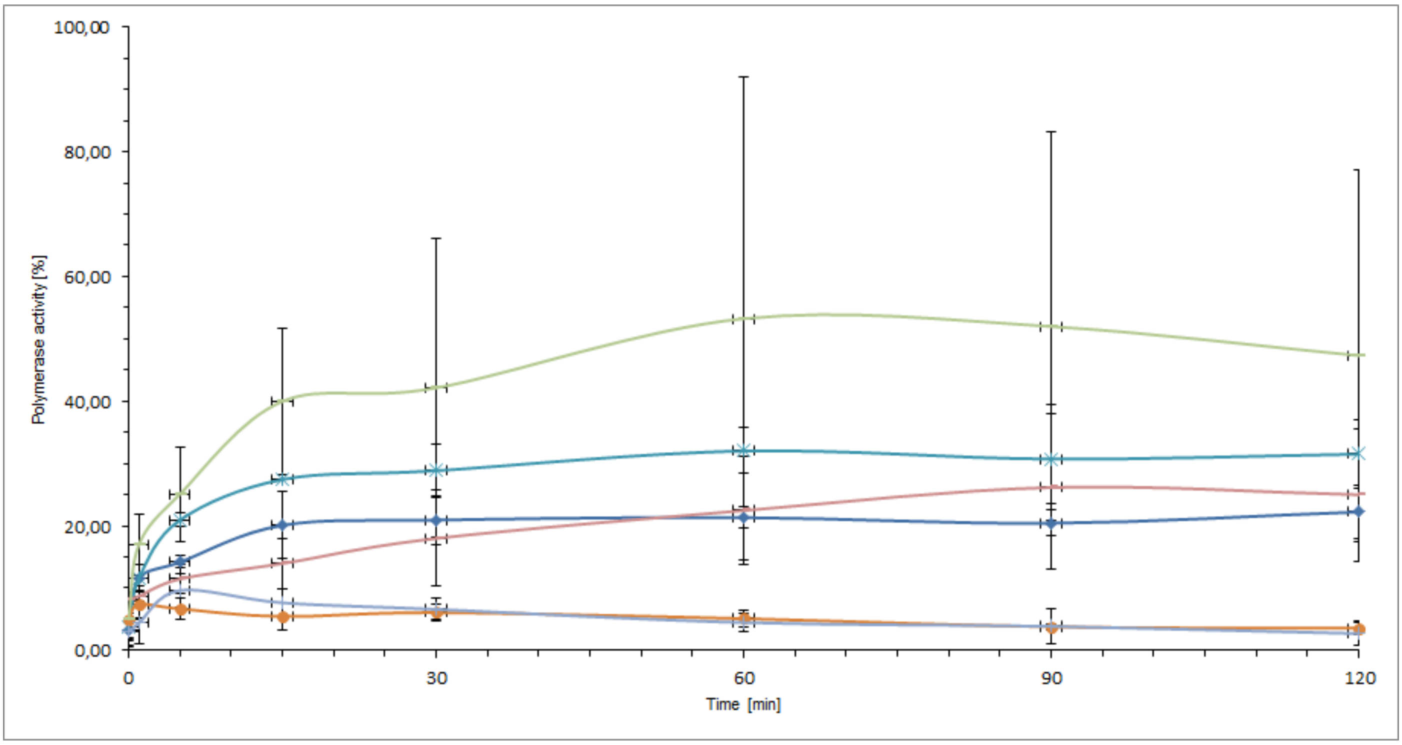

Figure S6. Graphical representation of DNA repair assays' results for **RcdA** – XPC Nuclear Extract. **(A)** ss-CDL, **(B)** ds-CDL

Table S3. Raw numerical data – RcdA – XPC Nuclear Extract.

RcdA		Time [min]							
		0	1	5	15	30	60	90	120
Strand	Data set	AP site rejoining [%]							
dU0	1.	90,46	2,40	0,22	0,06	0,10	0,07	0,01	0,00
	2.	91,61	7,20	4,09	1,48	1,13	1,07	0,61	1,32
	3.	57,24*	11,12	2,71	1,27	0,92	0,61	0,65	1,28
	Avg	91,03	6,91	2,34	0,94	0,72	0,58	0,43	0,87
	SD	0,82	4,37	1,96	0,77	0,54	0,50	0,36	0,75
dU-5	1.	95,64	5,41	0,84	3,73	0,31	0,18	0,25	0,38
	2.	86,97	26,63	3,75	1,77	1,36	1,36	1,48	1,07
	3.	61,41	23,93	4,62	1,61	1,29	1,03	0,89	1,54
	Avg	81,34	18,66	3,07	2,37	0,99	0,86	0,88	1,00
	SD	17,79	11,55	1,98	1,18	0,59	0,61	0,62	0,59
dU -5/+5	1.	96,92	1,55	0,29	0,13	0,09	0,03	0,00	0,00
	2.	92,17	9,35	1,20	0,32	0,10	0,00	0,00	0,00
	3.	73,08	10,67	2,00	1,16	0,65	0,30	0,20	0,72
	Avg	87,39	7,19	1,16	0,54	0,28	0,11	0,07	0,24
	SD	12,62	4,93	0,86	0,54	0,32	0,16	0,12	0,41
dU+5	1.	91,50	84,53	61,14	17,02	20,31	3,31	1,50	2,10
	2.	89,52	84,62	68,32	37,78	32,23	19,00	16,83	9,44
	3.	67,79	62,65	52,32	30,40	15,76	6,33	3,78	8,25
	Avg	82,94	77,27	60,59	28,40	22,76	9,55	7,37	6,59
	SD	13,15	12,66	8,02	10,52	8,51	8,32	8,27	3,94
dU-1	1.	93,82	18,07	5,14	3,86	9,95	19,03	21,66	23,33
	2.	78,70	31,75	10,46	7,31	12,33	13,96	24,12	22,97
	3.	93,02	85,84*	83,67*	83,29*	18,14*	24,30	20,85	28,04
	Avg	88,51	24,91	7,80	5,59	11,14	19,10	22,21	24,78
	SD	8,51	9,68	3,76	2,44	1,68	5,17	1,70	2,83
dU-4	1.	86,26	21,79	2,70	1,10	0,87	0,55	0,63	25,06
	2.	63,23	37,63	12,47	8,23	6,67	3,33	6,16	9,49
	3.	78,57	25,59	6,40	3,87	64,40	80,35	75,64	82,05
	Avg	76,02	28,33	7,19	4,40	23,98	28,08	27,48	38,87
	SD	11,72	8,27	4,93	3,59	35,12	45,29	41,80	38,20
dU+1	1.	94,84	18,21	3,10	10,42	15,46	0,46	0,00	0,00
	2.	75,44	35,50	14,07	6,99	4,82	3,11	4,18	2,42
	3.	93,02	18,60	5,14	2,90	2,11	1,31	1,07	1,32
	Avg	87,77	24,10	7,44	6,77	7,46	1,63	1,75	1,25
	SD	10,71	9,87	5,83	3,77	7,06	1,35	2,17	1,21
dU+4	1.	88,54	82,99	66,92	54,68	57,64	38,07	29,94	24,99
	2.	47,44*	47,18*	40,37*	39,95*	33,47*	34,11*	30,39*	25,17*
	3.	81,76	76,16	65,29	43,72	30,02	22,67	17,91	17,14
	Avg	85,15	79,57	66,10	49,20	43,83	30,37	23,92	21,07
	SD	4,79	4,83	1,15	7,74	19,53	10,89	8,51	5,55

* rejected values

RcdA		Time [min]							
		0	1	5	15	30	60	90	120
Strand	Data set	**Endonuclease activity [%]**							
dU0	1.	0,46	78,10	75,98	70,10	68,23	63,99	57,73	55,46
	2.	0,30	69,42	67,51	63,37	62,25	56,40	52,43	49,23
	3.	10,45*	53,09*	50,62*	47,04*	48,71*	41,07*	40,32*	37,28*
	Avg	0,38	73,76	71,75	66,74	65,24	60,20	55,08	52,35
	SD	0,11	6,14	5,99	4,76	4,23	5,37	3,75	4,40
dU-5	1.	0,18	74,22	69,05	54,72	44,15	26,51	21,04	17,72
	2.	0,38	56,53	69,04	59,01	47,04	31,79	21,49	19,00
	3.	2,66	45,42	51,55	43,21	40,38	34,64	30,58	24,54
	Avg	1,07	58,73	63,21	52,31	43,86	30,98	24,37	20,42
	SD	1,38	14,52	10,10	8,17	3,34	4,12	5,39	3,62
dU -5/+5	1.	0,00	85,65	80,49	70,63	59,46	49,93	48,43	52,25
	2.	0,22	70,49	72,88	64,33	55,01	52,41	43,56	42,26
	3.	2,23	64,37	59,21	52,21	46,79	44,25	39,63	39,85
	Avg	0,81	73,51	70,86	62,39	53,75	48,87	43,87	44,79
	SD	1,23	10,96	10,78	9,36	6,43	4,18	4,41	6,58
dU+5	1.	0,27	4,69	25,59	60,42*	48,47	49,74	40,30	32,43
	2.	1,26	5,59	18,66	37,03	37,44	34,19	33,49	34,48
	3.	0,23	3,75	14,92	28,81	35,12	35,78	34,04	28,22
	Avg	0,59	4,68	19,73	32,92	40,35	39,90	35,94	31,71
	SD	0,58	0,92	5,41	5,81	7,13	8,56	3,78	3,19
dU-1	1.	0,38	55,55	52,27	45,86	36,46	23,41	19,76	17,51
	2.	1,13	38,96	45,00	41,43	36,66	30,81	23,94	22,51
	3.	2,79*	5,54*	7,23*	7,96*	28,31*	25,75*	28,11*	26,09*
	Avg	0,76	47,25	48,64	43,65	36,56	27,11	21,85	20,01
	SD	0,53	11,73	5,15	3,13	0,14	5,23	2,96	3,54
dU-4	1.	2,30	59,88	73,14	45,12	35,82	23,04	28,12	11,23
	2.	9,03	37,70	48,11	44,30	36,75	17,24	18,52	19,92
	3.	8,79	35,02	35,29	36,10	4,65	1,89	2,08	2,58
	Avg	6,70	44,20	52,18	41,84	25,74	14,06	16,24	11,24
	SD	3,82	13,64	19,25	4,99	18,27	10,93	13,17	8,67
dU+1	1.	0,00	65,36	69,16	57,59	49,67	41,05	32,62	25,13
	2.	3,09	28,99	32,08	30,39	28,59	26,29	24,50	21,79
	3.	3,97	48,67	49,44	43,67	42,37	38,27	33,80	31,75
	Avg	2,35	47,67	50,23	43,88	40,21	35,21	30,31	26,22
	SD	2,09	18,21	18,55	13,60	10,71	7,84	5,06	5,07
dU+4	1.	0,00	0,60	14,59	25,28	14,46	25,96	26,59	23,41
	2.	1,45	3,23	15,89	18,97	24,29	21,44	21,05	21,56
	3.	2,33	2,71	7,98	25,23	26,23	23,15	19,33	18,53
	Avg	1,26	2,18	12,82	23,16	21,66	23,52	22,32	21,17
	SD	1,18	1,39	4,24	3,63	6,31	2,28	3,79	2,46

* rejected values

RcdA		Time [min]							
		0	1	5	15	30	60	90	120
Strand	Data set	**Polymerase activity [%]**							
dU0	1.	3,01	9,72	8,05	7,93	7,71	6,57	4,16	3,62
	2.	3,63	7,06	4,79	4,76	5,63	5,13	3,98	4,50
	3.	8,04	5,45	7,27	3,74	4,92	3,68	3,28	2,61
	Avg	4,90	7,41	6,70	5,48	6,09	5,13	3,81	3,58
	SD	2,74	2,16	1,70	2,18	1,45	1,44	0,47	0,94
dU-5	1.	1,30	12,92	20,48	33,58	47,94	67,99	74,14	78,25
	2.	4,01	7,66	14,79	29,11	41,46	58,22	68,47	71,95
	3.	9,34	12,53	24,21	32,26	45,68	53,20	57,37	63,84
	Avg	4,88	11,04	19,83	31,65	45,03	59,80	66,66	71,35
	SD	4,09	2,93	4,74	2,29	3,29	7,52	8,53	7,22
dU -5/+5	1.	0,93	8,28	12,43	20,94	32,74	40,03	38,49	34,43
	2.	1,41	8,15	13,32	22,87	35,00	41,95	45,43	38,03
	3.	5,42	10,29	18,70	31,54	36,25	38,68	39,46	37,35
	Avg	2,59	8,91	14,82	25,12	34,67	40,22	41,13	36,60
	SD	2,46	1,20	3,40	5,65	1,78	1,64	3,76	1,91
dU+5	1.	2,18	2,84	3,96	14,84	23,51	31,18	43,89	57,15
	2.	0,06	0,00	0,00	16,05	19,06	30,34	32,80	34,03
	3.	5,66	5,21	7,54	20,15	32,45	36,36	37,66	42,37
	Avg	2,63	2,68	3,83	17,01	25,01	32,63	38,12	44,51
	SD	2,83	2,61	3,77	2,78	6,82	3,26	5,56	11,71
dU-1	1.	2,12	13,14	21,67	27,42	31,86	34,62	35,80	35,47
	2.	5,33	10,07	20,20	27,47	25,87	29,42	25,67	27,55
	3.	0,02*	0,00*	0,00*	0,00*	5,37*	4,52*	5,24*	3,06*
	Avg	3,72	11,61	20,94	27,45	28,87	32,02	30,74	31,51
	SD	2,27	2,17	1,04	0,03	4,23	3,68	7,16	5,60
dU-4	1.	8,01	11,91	17,45	51,61	61,44	74,80	70,50	62,23
	2.	0,08	17,21	32,67	40,24	49,60	76,35	69,52	66,68
	3.	7,07	21,82	25,21	28,07	15,40	8,62	15,92	13,05
	Avg	5,05	16,98	25,11	39,97	42,15	53,26	51,98	47,32
	SD	4,33	4,96	7,61	11,77	23,91	38,67	31,23	29,76
dU+1	1.	2,32	6,17	8,17	6,06	4,91	2,88	0,92	0,60
	2.	5,73	6,68	8,02	6,73	6,55	5,28	6,36	2,95
	3.	1,15	0,43	12,75	10,25	8,38	5,33	4,40	4,61
	Avg	3,07	4,43	9,64	7,68	6,61	4,50	3,89	2,72
	SD	2,38	3,47	2,69	2,25	1,73	1,40	2,75	2,01
dU+4	1.	7,28	7,11	9,19	11,96	15,64	21,38	25,08	28,28
	2.	8,43	8,76	11,39	11,32	11,73	14,36	13,51	13,14
	3.	9,06	10,25	13,88	18,66	26,60	31,64	39,91	33,59
	Avg	8,26	8,71	11,49	13,98	17,99	22,46	26,16	25,00
	SD	0,90	1,57	2,34	4,07	7,71	8,69	13,23	10,62

* rejected values

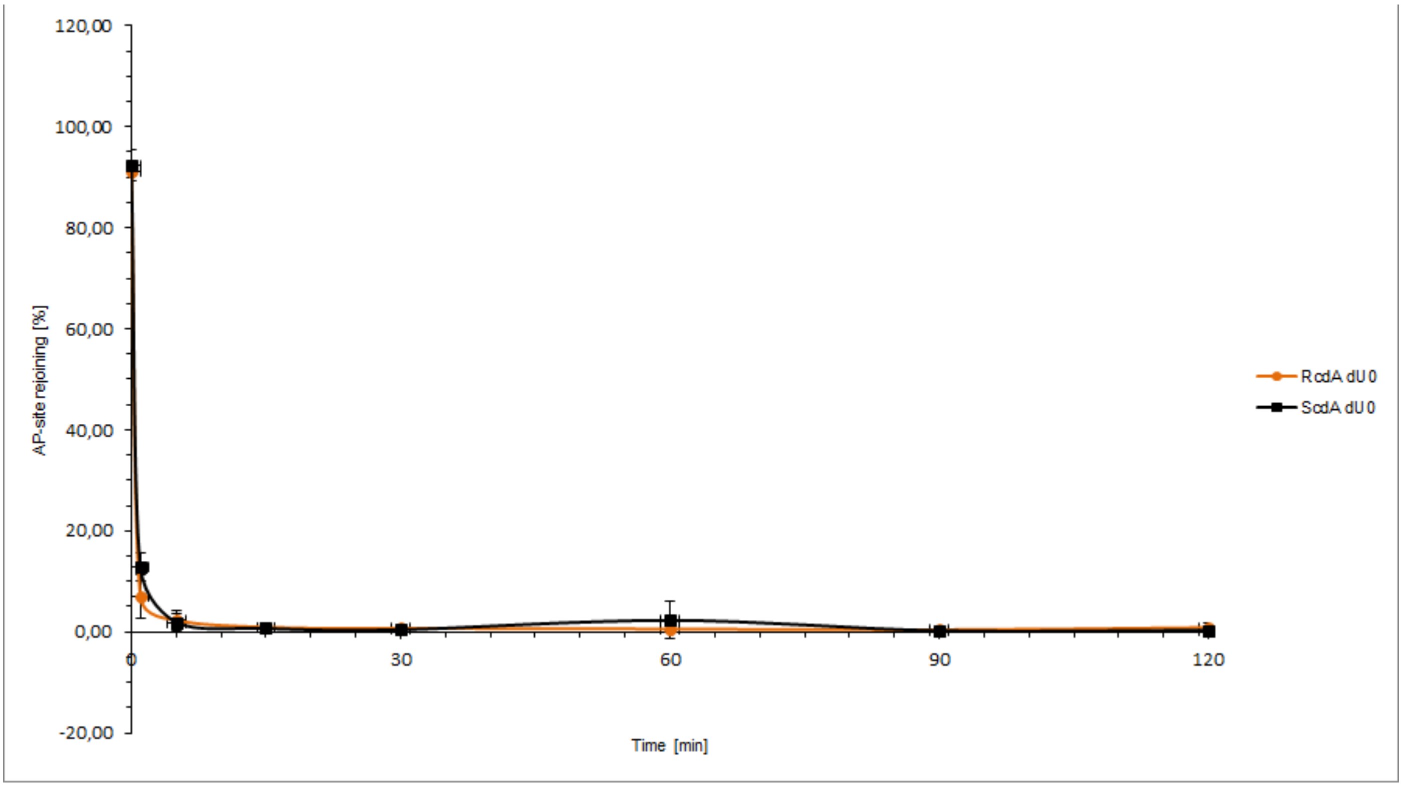

120,00
100,00
80,00
60,00
40,00
20,00
0,00
-20,00
AP-site rejoining [%]
0
30
60
90
120
Time [min]
RcdA dU 0
ScdA dU 0

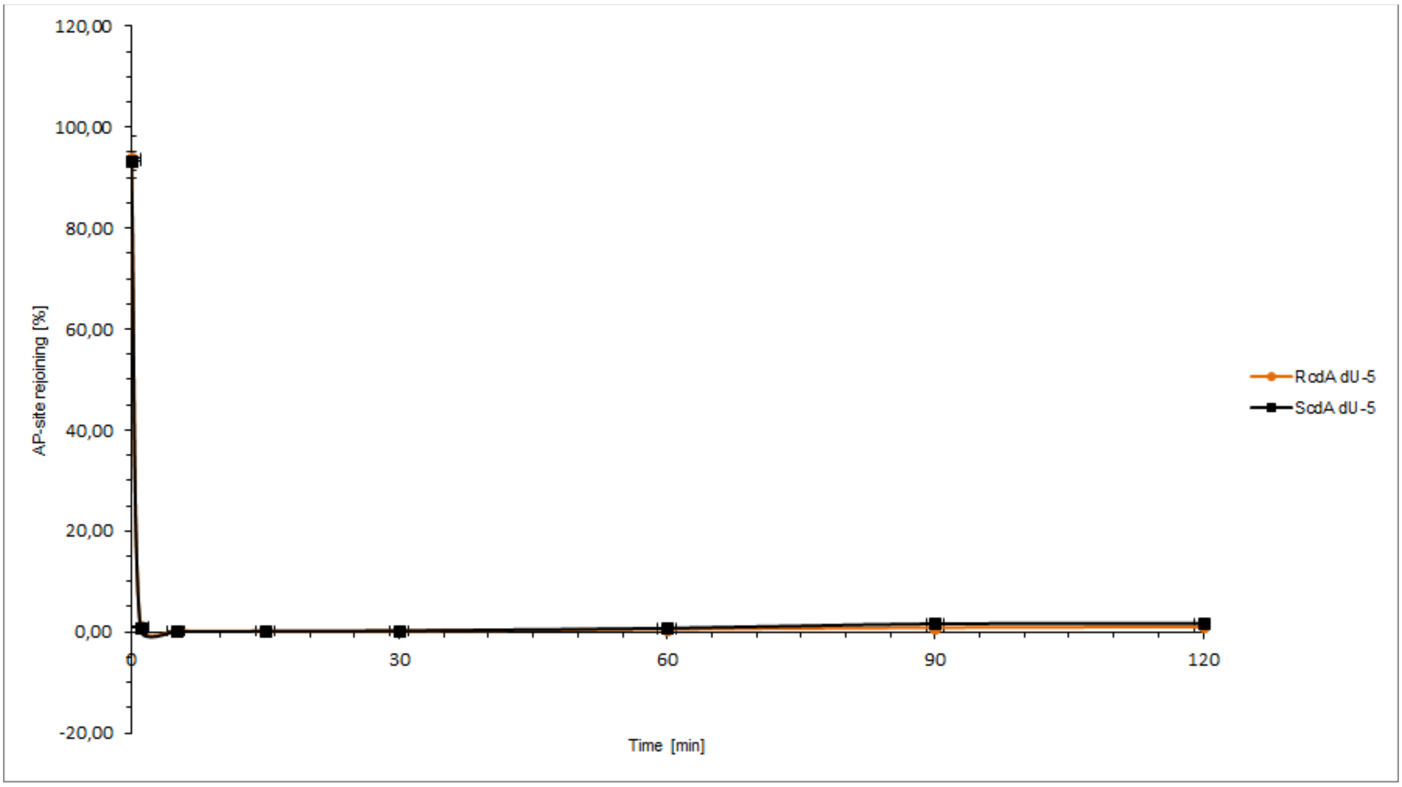

120,00
100,00
80,00
60,00
40,00
20,00
0,00
-20,00
AP-site rejoining [%]
0
30
60
90
120
Time [min]
RcdA dU-5
ScdA dU-5

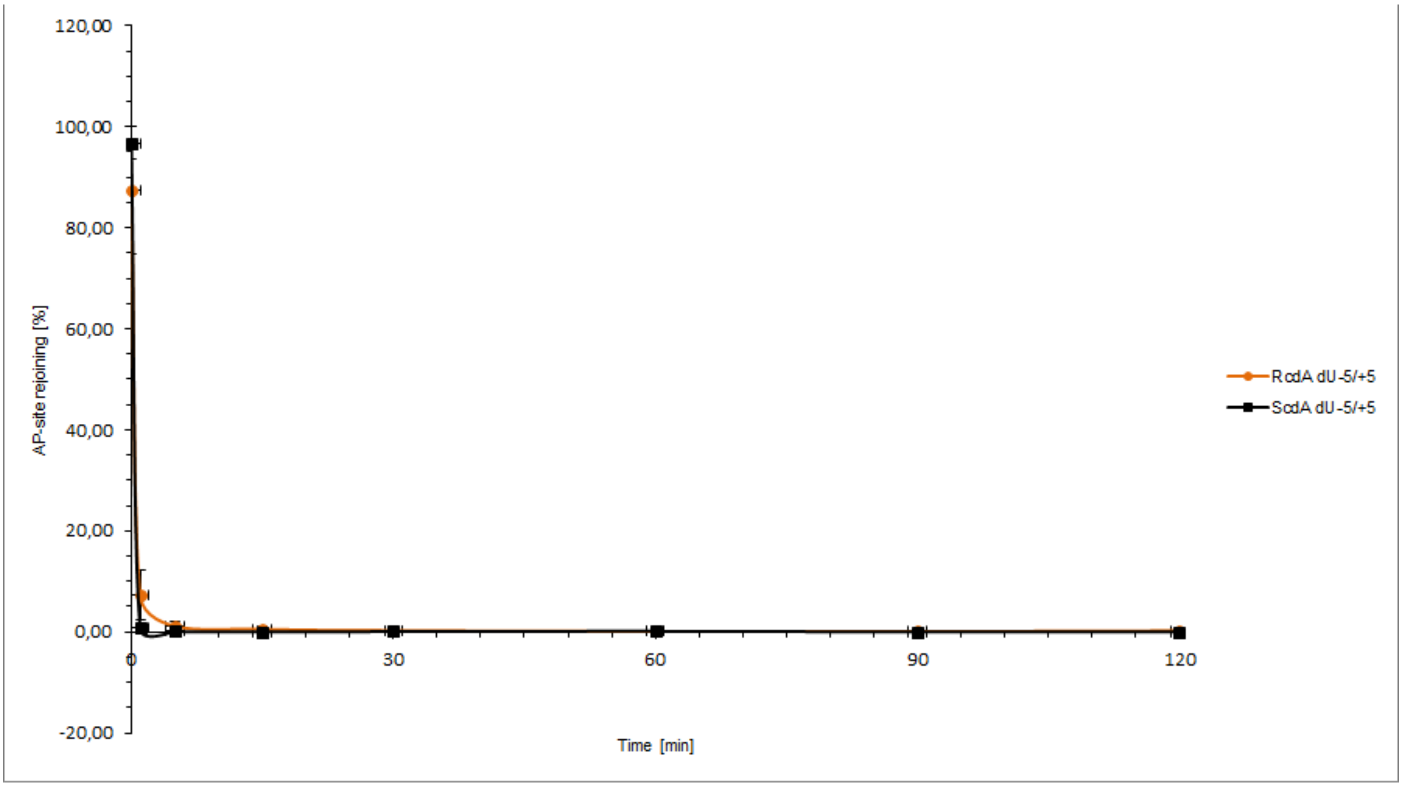
120,00
100,00
80,00
60,00
40,00
20,00
0,00
-20,00
AP-site rejoining [%]
0
30
60
90
120
Time [min]
RcdA dU-5/+5
ScdA dU-5/+5

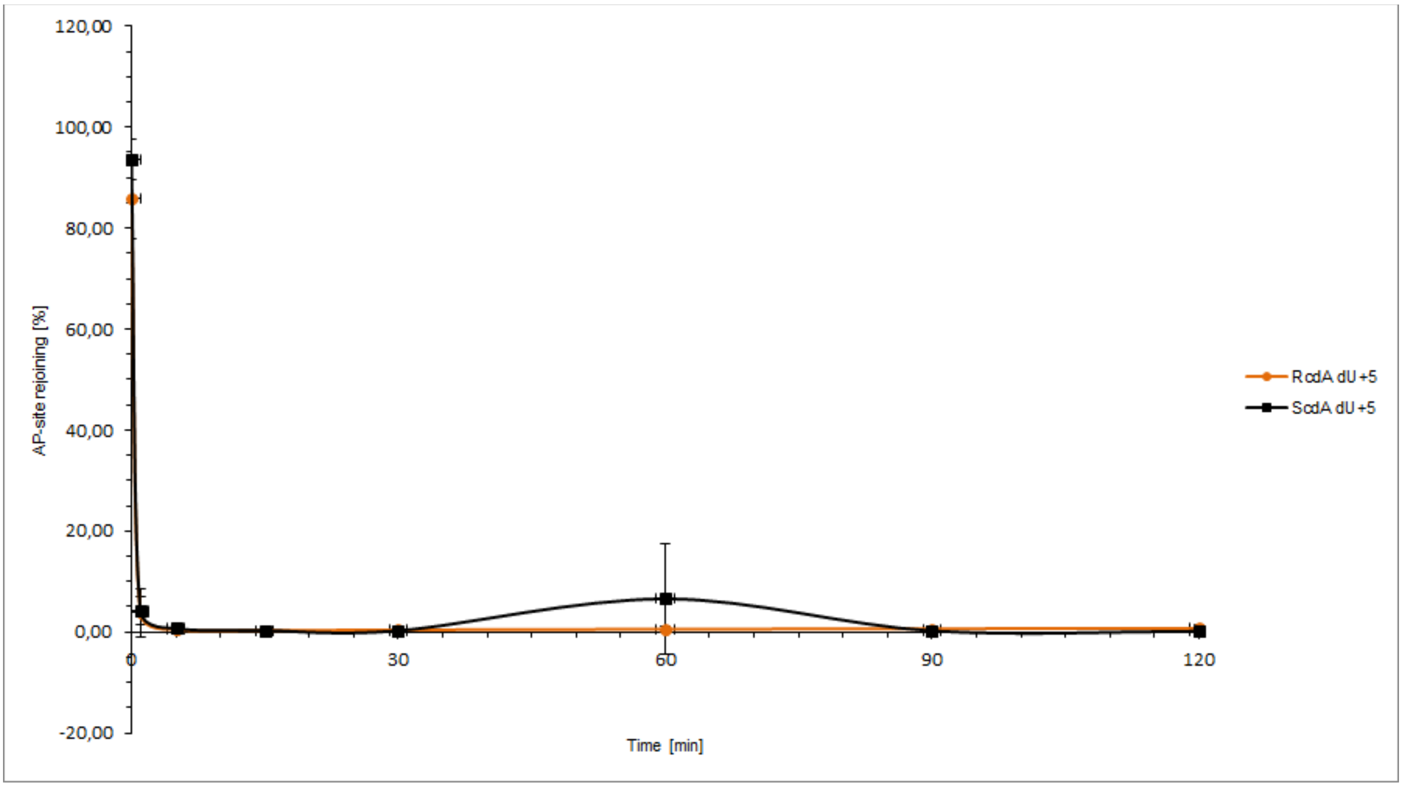
120,00
100,00
80,00
60,00
40,00
20,00
0,00
-20,00
AP-site rejoining [%]
0
30
60
90
120
Time [min]
RcdA dU+5
ScdA dU+5

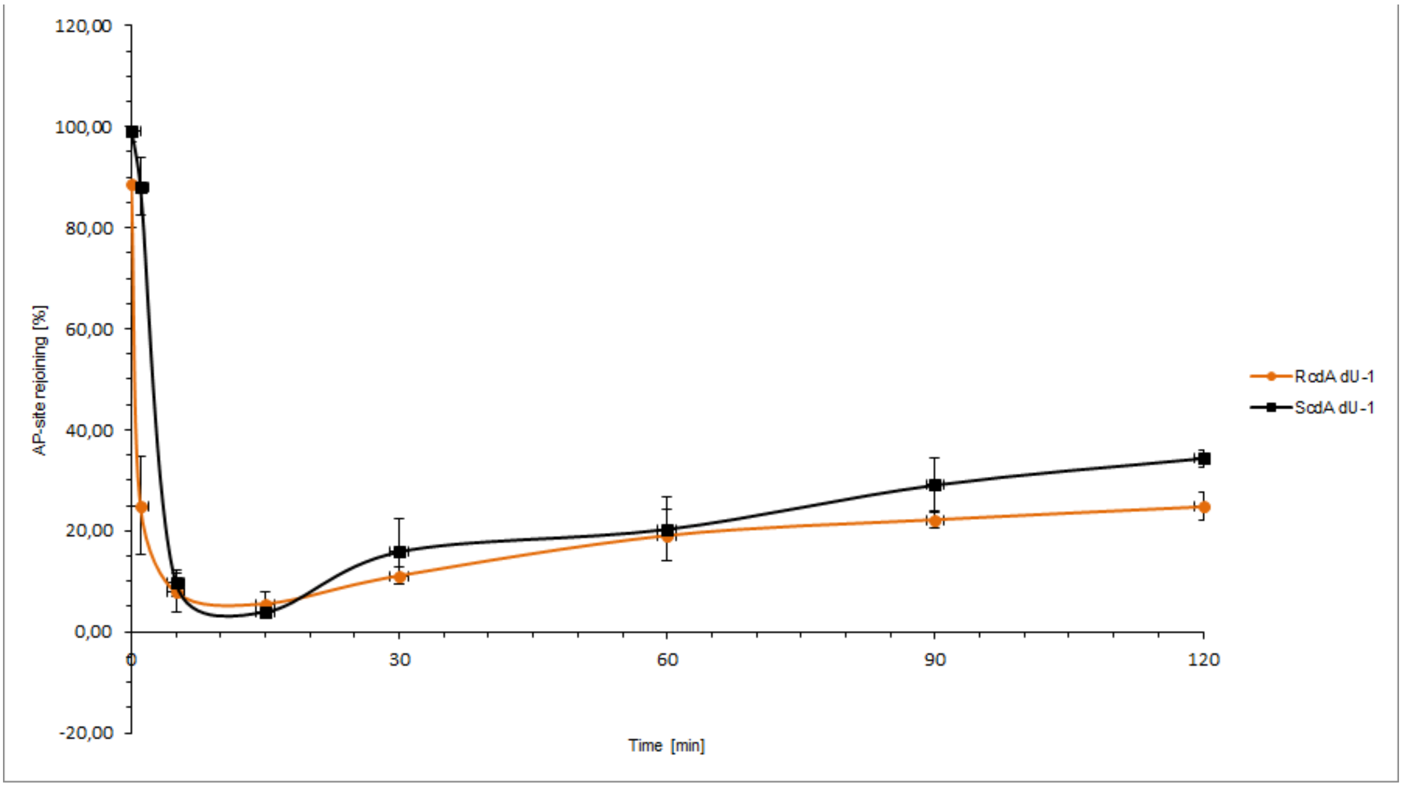
120,00
100,00
80,00
60,00
40,00
20,00
0,00
-20,00
AP-site rejoining [%]
0
30
60
90
120
Time [min]
RcdA dU-1
ScdA dU-1

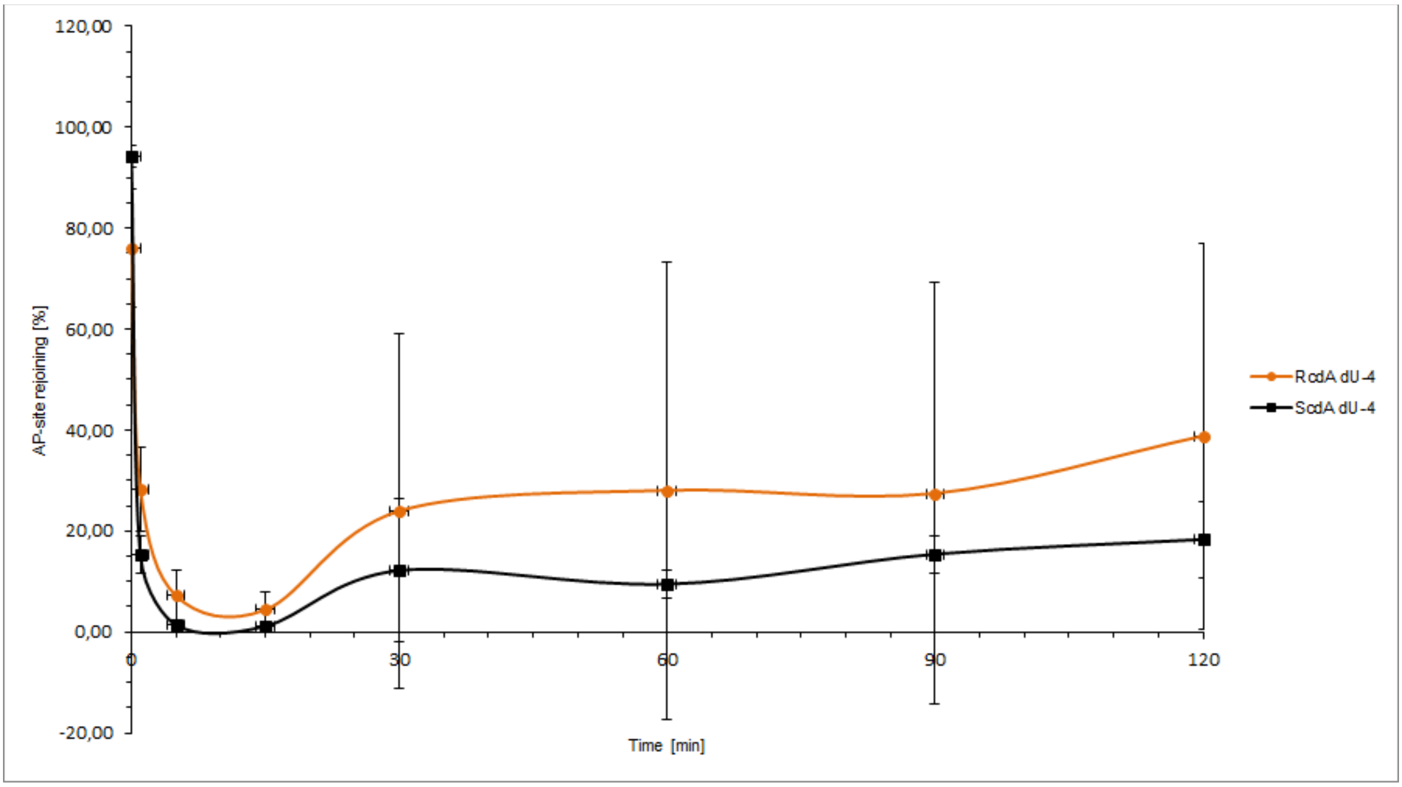
120,00
100,00
80,00
60,00
40,00
20,00
0,00
-20,00
AP-site rejoining [%]
0
30
60
90
120
Time [min]
RcdA dU-4
ScdA dU-4

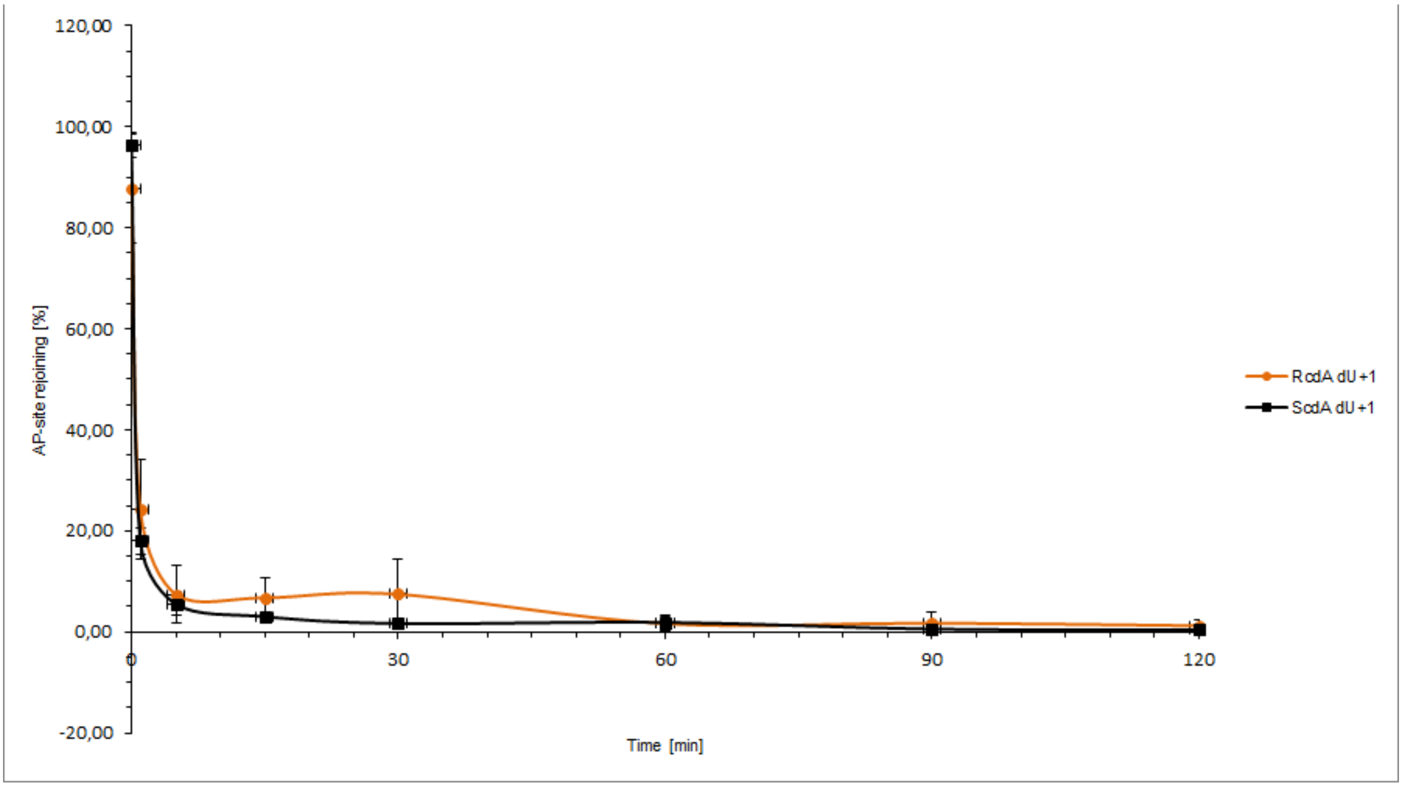

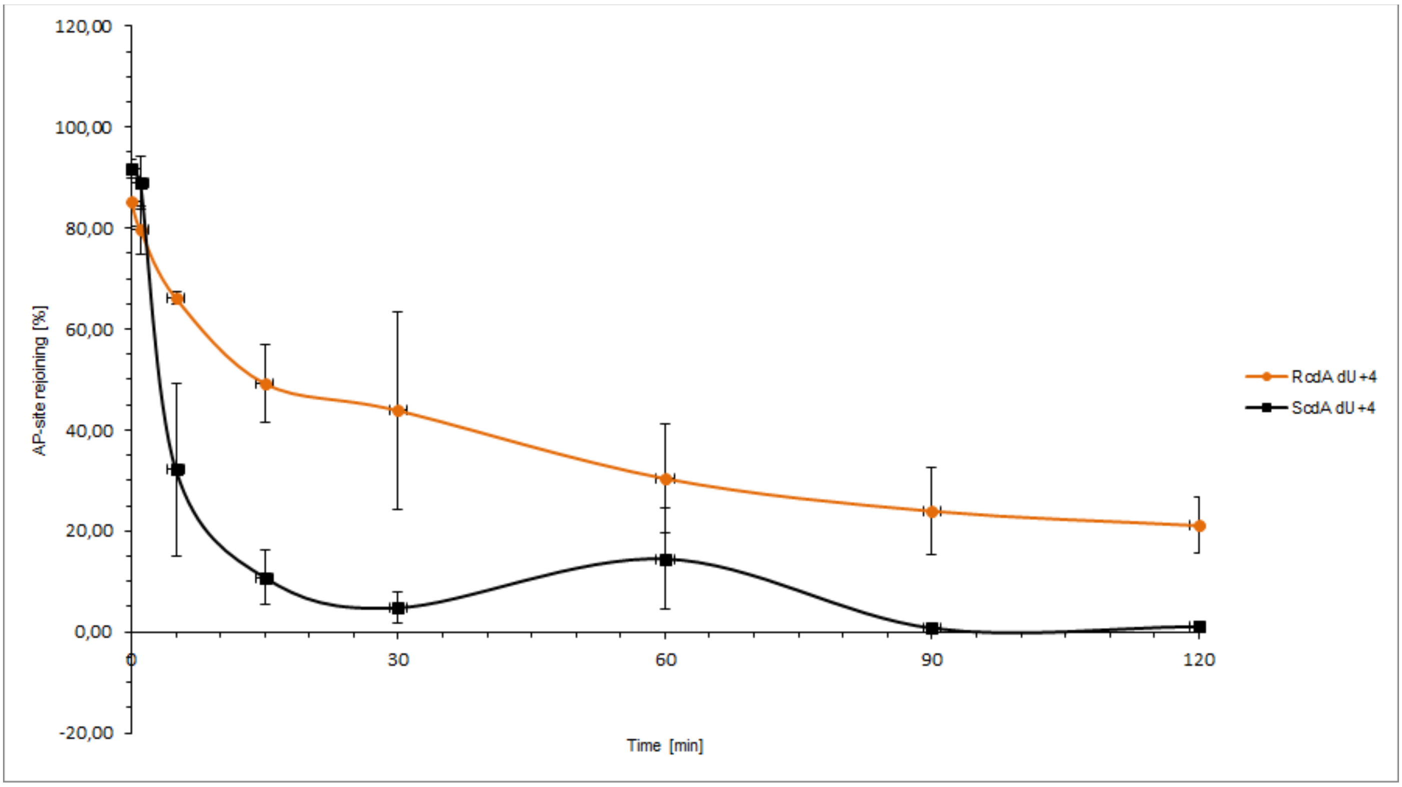

Figure S7. AP site rejoining [%] of ScdA vs. RcdA – comparison of individual strands – XPC Nuclear Extract.

A.

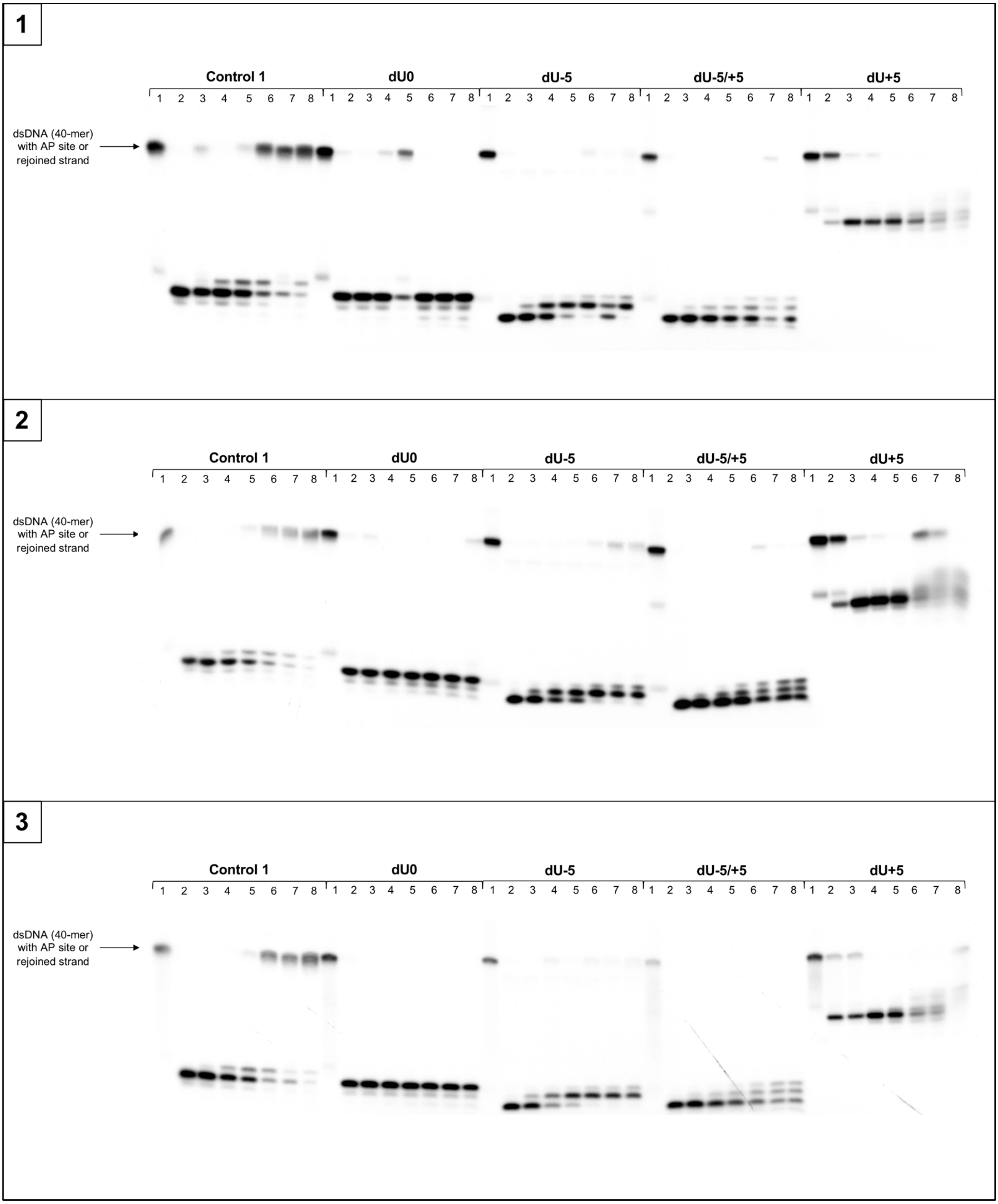
1
Control 1
dU0
dU-5
dU-5/+5
dU+5
1 2 3 4 5 6 7 8
dsDNA (40-mer) with AP site or rejoined strand
2
Control 1
dU0
dU-5
dU-5/+5
dU+5
1 2 3 4 5 6 7 8
dsDNA (40-mer) with AP site or rejoined strand
3
Control 1
dU0
dU-5
dU-5/+5
dU+5
1 2 3 4 5 6 7 8
dsDNA (40-mer) with AP site or rejoined strand

B.

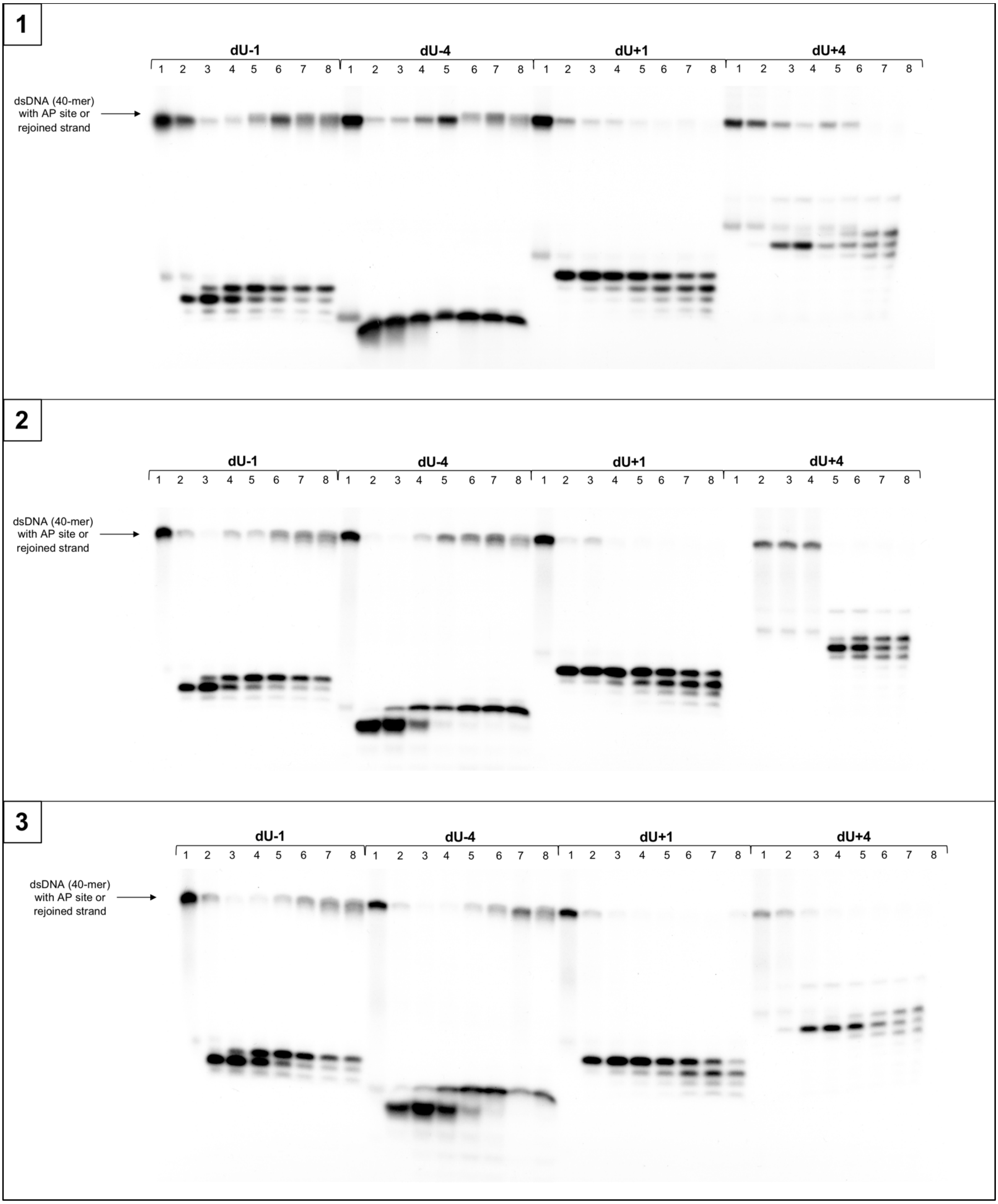

Figure S8. The autoradiograms presenting repair of **(A)** ss-CDL and **(B)** ds-CDL containing **ScdA** – BJ Nuclear Extract.
Lanes correspond with different assay time: lane 1 - 0 min; lane 2 - 1 min; lane 3 – 5 min; lane 4 – 15 min; lane 5 – 30 min; lane 6 – 60 min; lane 7 – 90 min; lane 8 – 120 min. Three replications of the experiment are shown.

A.

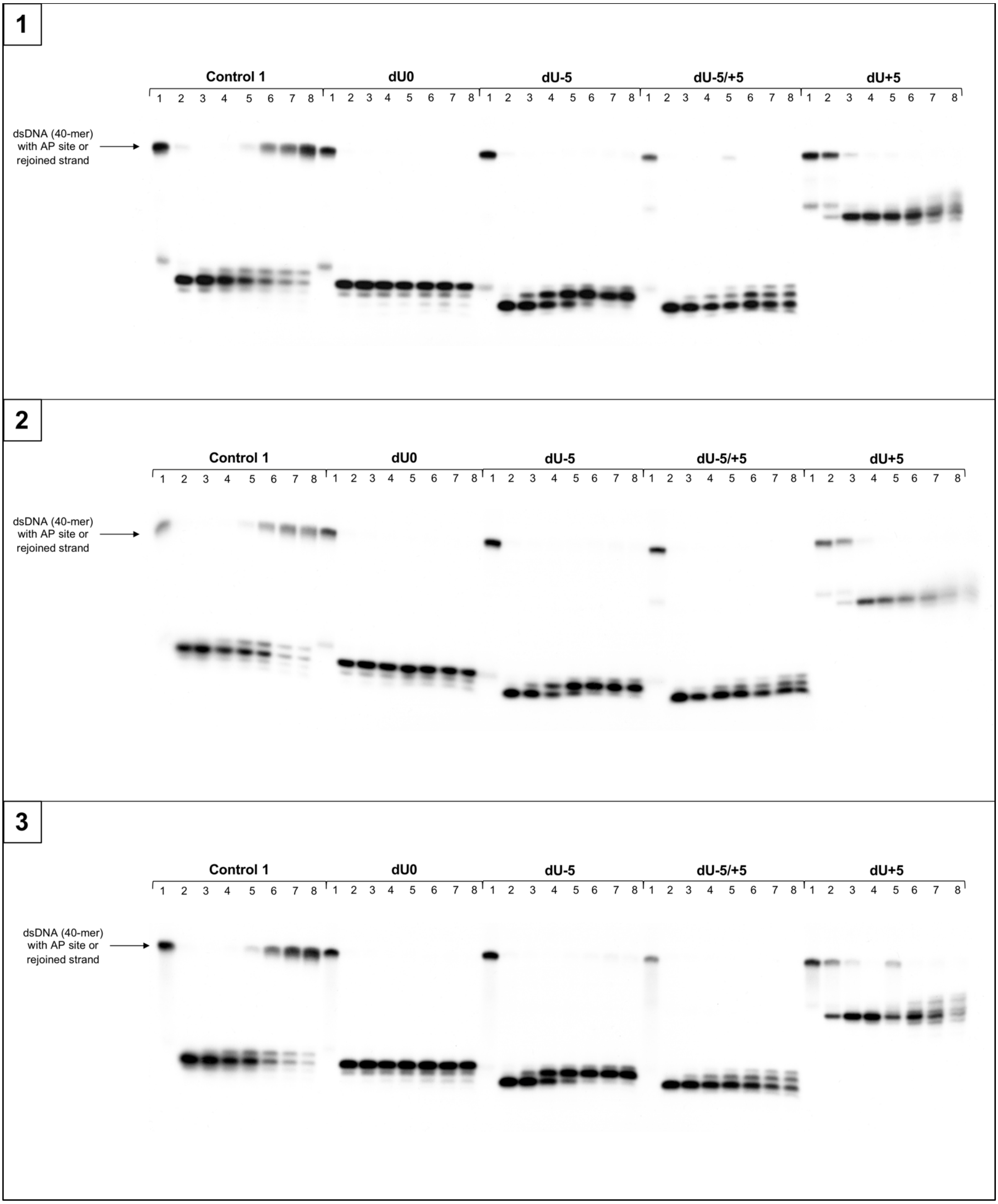
1
Control 1
dU0
dU-5
dU-5/+5
dU+5
1 2 3 4 5 6 7 8
dsDNA (40-mer) with AP site or rejoined strand
2
Control 1
dU0
dU-5
dU-5/+5
dU+5
1 2 3 4 5 6 7 8
dsDNA (40-mer) with AP site or rejoined strand
3
Control 1
dU0
dU-5
dU-5/+5
dU+5
1 2 3 4 5 6 7 8
dsDNA (40-mer) with AP site or rejoined strand

B.

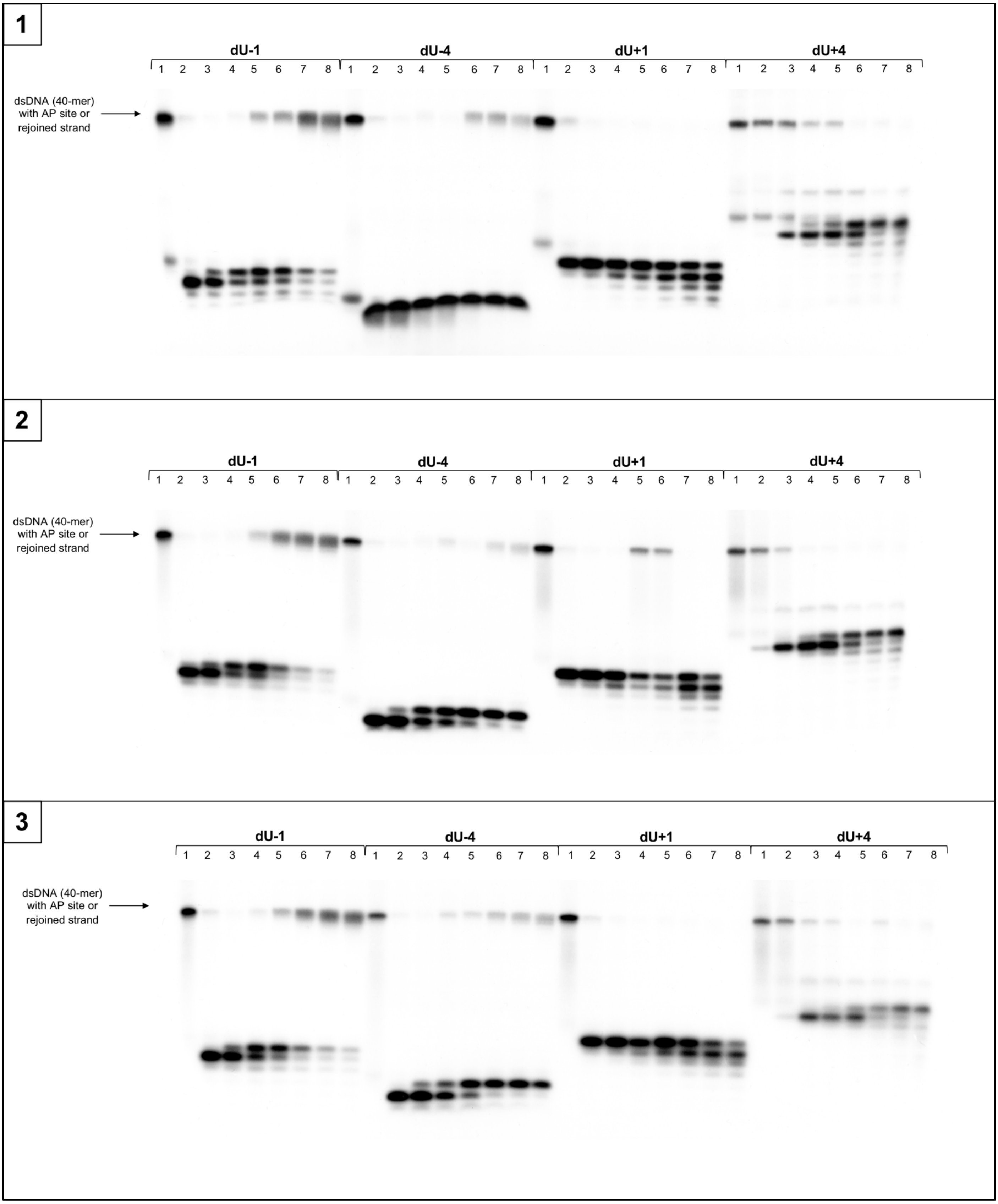

Figure S9. The autoradiograms presenting repair of **(A)** ss-CDL and **(B)** ds-CDL containing **RcdA** – BJ Nuclear Extract.
Lanes correspond with different assay time: lane 1 - 0 min; lane 2 - 1 min; lane 3 – 5 min; lane 4 – 15 min; lane 5 – 30 min; lane 6 – 60 min; lane 7 – 90 min; lane 8 – 120 min. Three replications of the experiment are shown.

A.

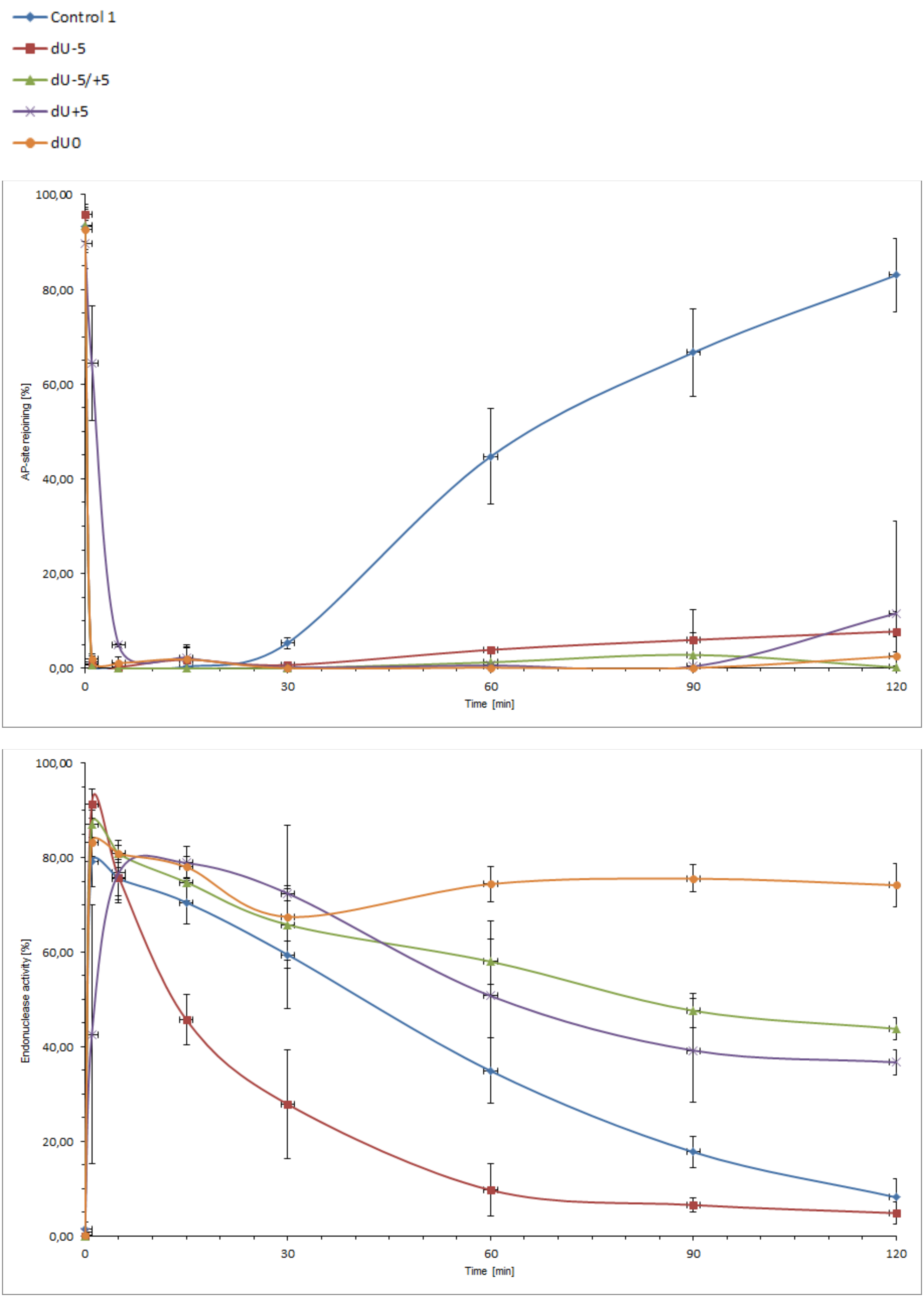
Control 1
dU-5
dU-5/+5
dU+5
dU0
100,00
80,00
60,00
40,00
20,00
0,00
AP-site rejoining [%]
0
30
60
90
120
Time [min]
100,00
80,00
60,00
40,00
20,00
0,00
Endonuclease activity [%]
0
30
60
90
120
Time [min]

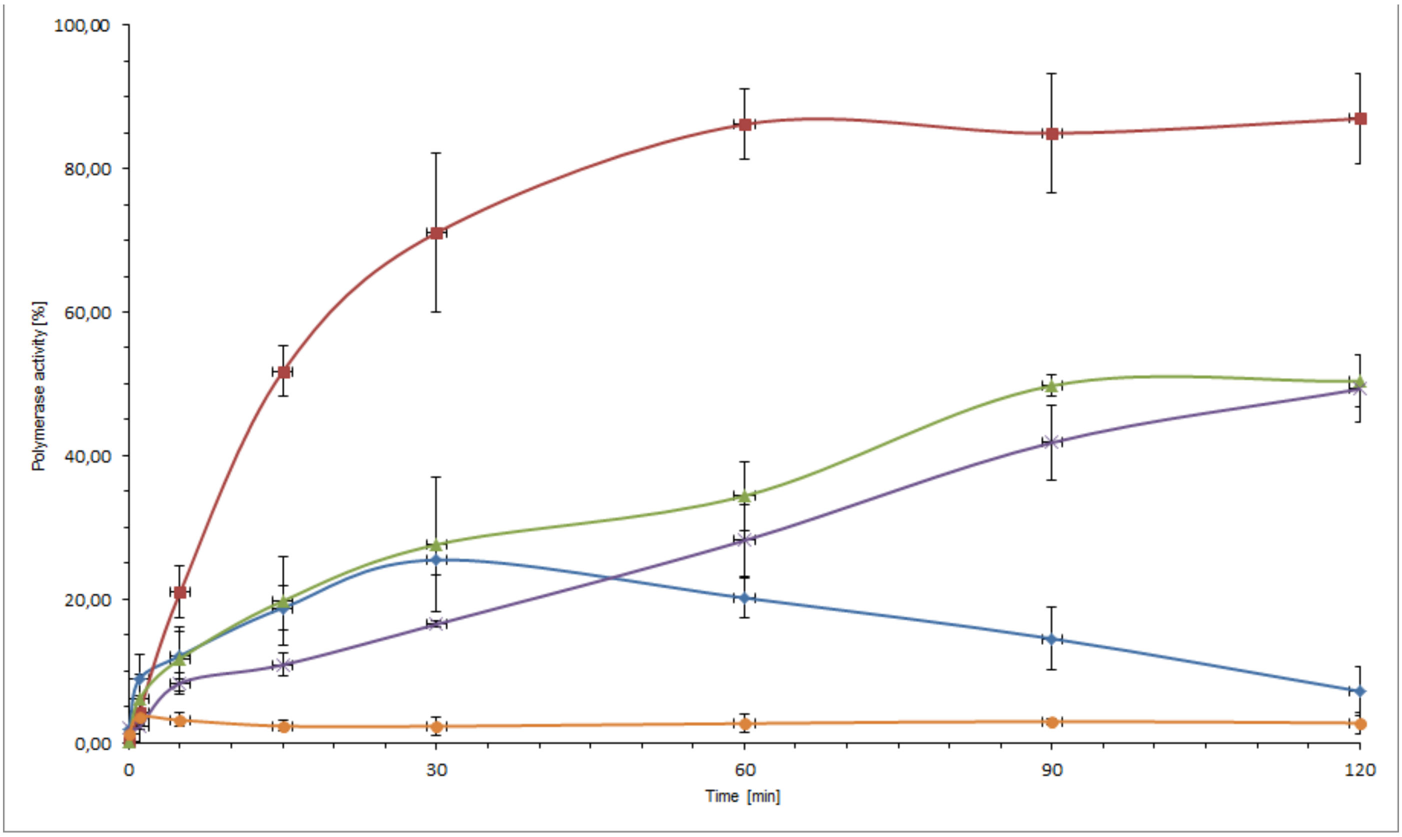
100,00
80,00
60,00
40,00
20,00
0,00
Polymerase activity [%]
0
30
60
90
120
Time [min]

B.

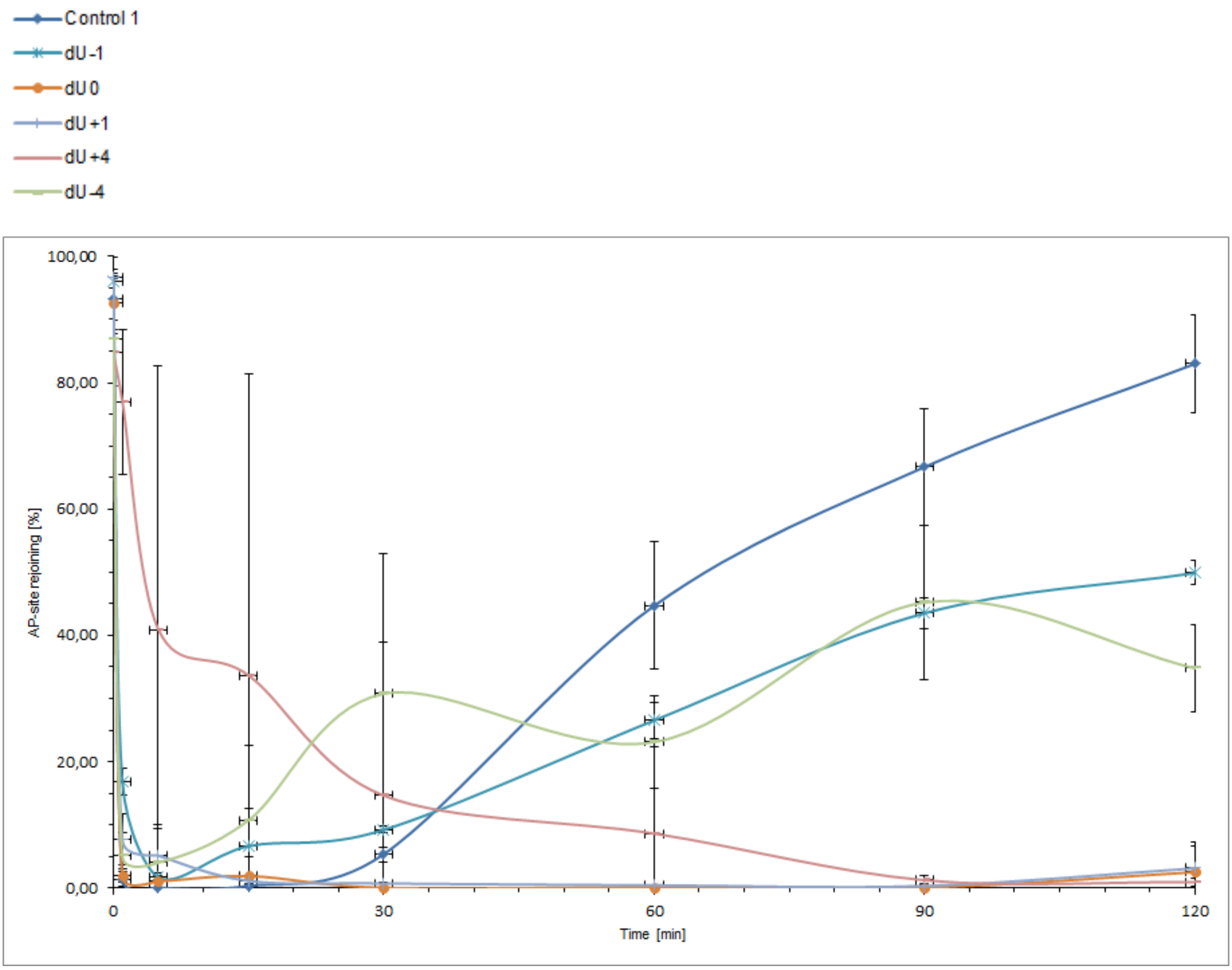
Control 1
dU -1
dU 0
dU +1
dU +4
dU -4
100,00
80,00
60,00
40,00
20,00
0,00
AP-site rejoining [%]
0
30
60
90
120
Time [min]

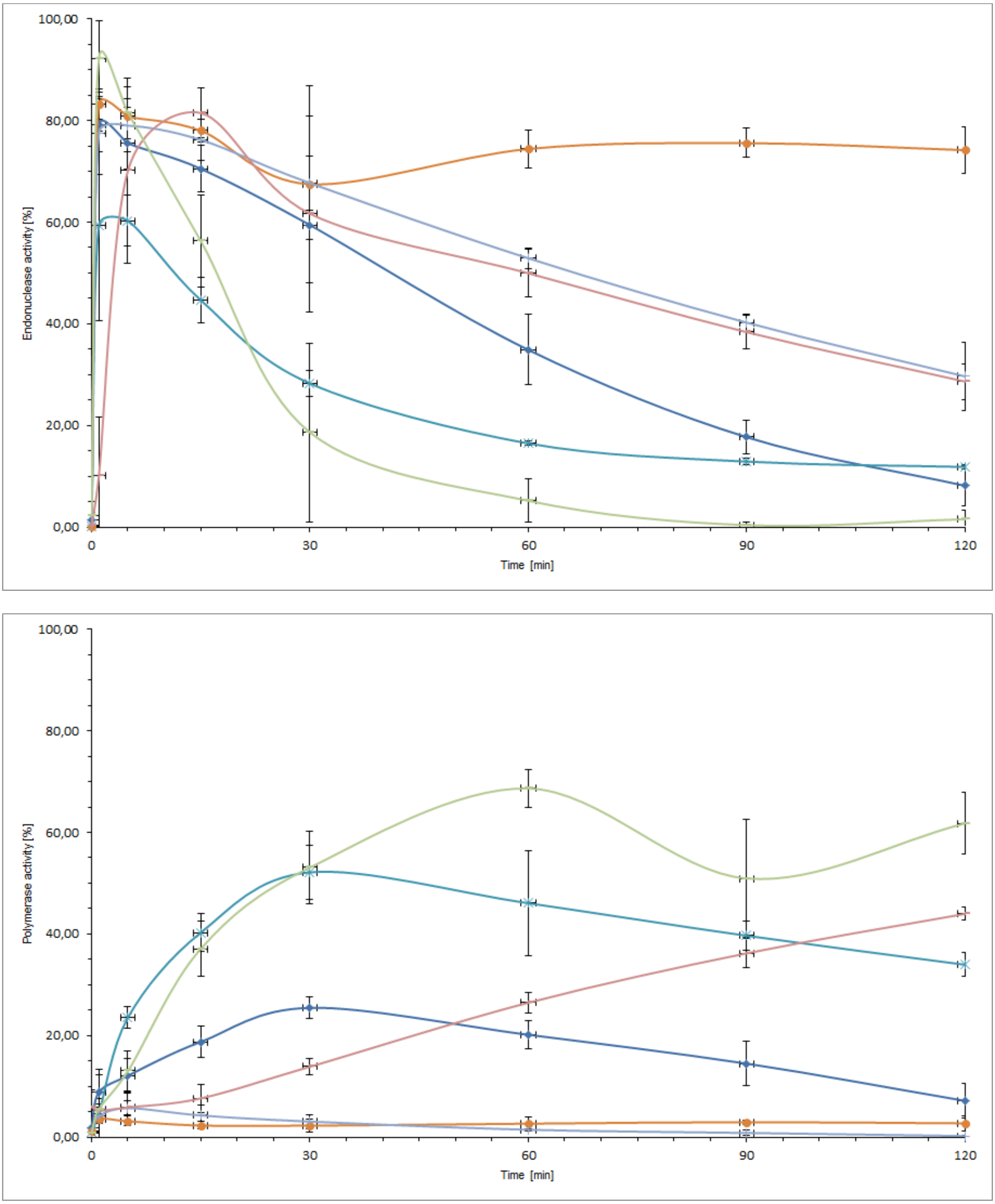

Figure S10. Graphical representation of DNA repair assays' results for ScdA – BJ Nuclear Extract. **(A)** ss-CDL, **(B)** ds-CDL

Table S4. Raw numerical data – Control 1 – BJ Nuclear Extract. * rejected values

		Time [min]							
		0	1	5	15	30	60	90	120
Strand	Data set	**AP site rejoining [%]**							
Control 1	1.	93,83	0,87	9,73*	0,55	6,20	52,04	73,82	79,86
	2.	95,42	0,70	0,06	0,28	6,11	37,93	66,81	90,54
	3.	97,67	0,23	0,05	0,15	4,78	57,57	72,02	91,79
	4.	85,07	3,42	0,05	0,33	4,59	32,76	49,07	74,22
	5.	90,50	2,16	0,17	0,27	3,74	23,88*	66,10	75,09
	6.	96,71	1,03	0,14	0,65	6,77	43,21	72,09	86,36
	Avg	93,20	1,40	0,09	0,37	5,37	44,70	66,65	82,98
	SD	4,71	1,18	0,06	0,19	1,17	10,13	9,16	7,68

		Time [min]							
		0	1	5	15	30	60	90	120
Strand	Data set	**Endonuclease activity [%]**							
Control 1	1.	0,23	80,03	74,09	67,73	58,27	25,92	20,45	8,17
	2.	1,27	80,28	78,76	74,25	61,83	32,64	14,02	4,09
	3.	0,93	83,90	81,89	76,37	61,45	21,84*	21,61	4,06
	4.	0,51	80,97	74,28	68,57	61,48	39,63	27,69*	13,27
	5.	4,01	70,23	68,65	65,76	59,42	43,68	17,60	12,13
	6.	1,70	65,43*	61,13*	58,62*	54,26	32,60	14,89	7,20
	Avg	1,44	79,08	75,54	70,53	59,45	34,90	17,72	8,15
	SD	1,36	5,18	5,05	4,54	2,90	6,90	3,33	3,90

		Time [min]							
		0	1	5	15	30	60	90	120
Strand	Data set	**Polymerase activity [%]**							
Control 1	1.	1,50	4,93*	6,07*	16,77	26,85	18,67	5,01*	10,94
	2.	2,11	9,12	10,77	16,53	24,13	24,03	16,10	4,00
	3.	0,83	5,16	8,02	15,38	28,51	18,25	4,94*	3,74
	4.	1,79	5,74	10,81	19,83	23,87	20,91	19,75	10,52
	5.	4,22	10,23	15,68	23,15	26,58	22,57	12,07	8,93
	6.	0,69	13,82	15,29	20,90	22,93	16,64	10,02	4,81
	Avg	1,86	8,81	12,11	18,76	25,48	20,18	14,48	7,16
	SD	1,28	3,53	3,28	3,01	2,15	2,81	4,32	3,34

Table S5. Raw numerical data – ScdA – BJ Nuclear Extract.

ScdA		Time [min]							
		0	1	5	15	30	60	90	120
Strand	Data set	**AP site rejoining [%]**							
dU0	1.	88,26	2,96	0,60	5,53	50,05*	0,26	0,08	0,09
	2.	91,72	1,89	2,67	0,15	0,07	0,11	0,08	7,40
	3.	97,67	0,95	0,13	0,07	0,05	0,05	0,07	0,15
	Avg	92,55	1,93	1,13	1,92	0,06	0,14	0,08	2,55
	SD	4,76	1,01	1,35	3,13	0,02	0,10	0,01	4,20
dU-5	1.	95,18	1,14	0,08	0,05	0,15	3,89	1,20	3,86
	2.	94,84	0,81	0,58	0,61	0,69	3,30	13,19	12,27
	3.	97,16	0,54	0,33	4,78	1,27	4,53	3,62	7,22
	Avg	95,72	0,83	0,33	1,81	0,70	3,91	6,00	7,78
	SD	1,25	0,30	0,25	2,58	0,56	0,61	6,34	4,23
dU -5/+5	1.	93,97	1,34	0,02	0,01	0,02	0,07	8,16	0,02
	2.	92,13	0,47	0,03	0,02	0,05	3,70	0,30	0,39
	3.	94,24	0,17	0,00	0,02	0,07	0,04	0,06	0,21
	Avg	93,44	0,66	0,02	0,02	0,04	1,27	2,84	0,20
	SD	1,15	0,61	0,01	0,00	0,03	2,10	4,61	0,18
dU+5	1.	91,16	72,97	4,68	4,60	0,23	0,40	0,29	0,32
	2.	83,71	55,86	5,06	1,81	0,39	28,91*	18,35*	0,51
	3.	94,07	19,43*	18,35*	0,20	0,18	0,81	0,54	34,00*
	Avg	89,65	64,42	4,87	2,20	0,27	0,61	0,41	11,61
	SD	5,34	12,10	0,27	2,23	0,11	0,29	0,18	19,39
dU-1	1.	91,59	49,93*	7,14*	6,49*	18,98*	45,60*	45,54	47,86
	2.	98,97	15,36	1,46	10,89	9,59	28,61	40,85	50,21
	3.	97,29	18,35	1,94	2,43	8,86	24,49	44,15	51,59
	Avg	95,95	16,85	1,70	6,66	9,22	26,55	43,52	49,88
	SD	3,87	2,12	0,34	5,98	0,52	2,91	2,41	1,89
dU-4	1.	75,74	9,06	11,00	23,93	49,05	21,68	37,05	33,89
	2.	94,89	1,91	0,53	7,42	37,02	31,05	39,40	28,37
	3.	89,85	4,20	0,55	0,88	6,24	16,63	59,20	42,11
	Avg	86,83	5,06	4,03	10,74	30,77	23,12	45,22	34,79
	SD	9,93	3,65	6,04	11,88	22,08	7,32	12,17	6,91
dU+1	1.	88,71*	21,63*	7,38*	6,25*	3,24*	1,92*	2,95*	1,55*
	2.	96,53	4,79	8,17	1,21	0,71	0,46	0,27	0,23
	3.	96,74	10,51	2,15	1,10	0,80	0,46	0,43	6,07
	Avg	96,64	7,65	5,16	1,16	0,76	0,46	0,35	3,15
	SD	0,14	4,04	4,26	0,08	0,07	0,00	0,11	4,13
dU+4	1.	78,67	77,33	28,66	10,70	42,66	24,42	1,95	1,57
	2.	88,25	88,28	87,31	88,53	0,48	0,54	0,75	0,37
	3.	87,21	65,35	6,81	1,82	0,83	0,98	1,06	0,95
	Avg	84,71	76,98	40,93	33,68	14,65	8,65	1,26	0,96
	SD	5,26	11,47	41,63	47,70	24,25	13,67	0,62	0,60

* rejected values

ScdA		Time [min]							
		0	1	5	15	30	60	90	120
Strand	Data set	**Endonuclease activity [%]**							
dU0	1.	0,12	80,77	80,85	75,67	45,25	71,40	73,56	71,46
	2.	0,37	82,46	79,06	78,28	76,98	73,28	74,32	71,59
	3.	0,01	86,45	82,57	80,10	80,24	78,61	78,87	79,54
	Avg	0,17	83,23	80,83	78,02	67,49	74,43	75,58	74,20
	SD	0,18	2,92	1,75	2,22	19,33	3,74	2,88	4,62
dU-5	1.	0,00	94,94	78,94	50,84	21,75	5,55	44,83*	3,01
	2.	0,00	88,86	71,56	45,92	41,07	15,96	7,59	7,40
	3.	0,00	90,48	76,88	40,35	20,64	7,53	5,42	3,97
	Avg	0,00	91,43	75,79	45,70	27,82	9,68	6,51	4,79
	SD	0,00	3,15	3,81	5,25	11,49	5,53	1,53	2,31
dU -5/+5	1.	0,00	90,28	84,05	78,50	74,55	63,06	50,98	61,31*
	2.	0,00	84,54	78,70	70,75	61,45	57,33	48,05	45,52
	3.	0,00	86,61	79,71	75,12	61,48	53,66	43,83	42,08
	Avg	0,00	87,14	80,82	74,79	65,82	58,02	47,62	43,80
	SD	0,00	2,91	2,84	3,88	7,56	4,74	3,59	2,43
dU+5	1.	0,21	20,30	83,04	77,61	72,85	64,01	48,20	34,86
	2.	0,94	34,18	75,84	76,28	70,73	33,15	26,99	38,55
	3.	1,13	73,13	71,69	82,74	73,75	55,11	42,42	9,40*
	Avg	0,76	42,54	76,85	78,88	72,44	50,76	39,20	27,60
	SD	0,48	27,38	5,74	3,41	1,55	15,88	10,97	15,87
dU-1	1.	0,00	39,21	55,39	42,41	26,06	16,05	12,22	11,72
	2.	0,04	76,37	65,38	41,77	27,82	16,48	13,00	11,55
	3.	0,35	62,19	60,10	49,88	30,97	16,92	13,48	12,27
	Avg	0,13	59,26	60,29	44,68	28,28	16,48	12,90	11,85
	SD	0,19	18,75	4,99	4,51	2,49	0,44	0,64	0,38
dU-4	1.	3,06*	50,59*	60,22	33,05	5,11	10,06	5,99	3,85
	2.	0,35	97,40	85,14	49,92	6,21	2,18	0,75	2,78
	3.	4,19	86,93	77,98	62,72	31,07	8,21	0,00	0,35
	Avg	2,27	92,16	81,56	56,32	18,64	5,19	0,38	1,56
	SD	2,72	7,40	5,06	9,05	17,58	4,27	0,53	1,72
dU+1	1.	0,00	68,20	74,13	73,46	65,34	51,74	40,31	32,77
	2.	0,48	82,96	78,44	74,38	63,97	51,80	41,97	34,33
	3.	0,32	81,16	84,53	80,82	73,86	55,21	38,49	21,92
	Avg	0,27	77,44	79,03	76,22	67,72	52,92	40,26	29,67
	SD	0,24	8,05	5,23	4,01	5,36	1,99	1,74	6,76
dU+4	1.	0,00	1,88	57,25	78,08	39,53	44,86	41,78	32,47
	2.	0,11	0,12*	0,17*	0,27*	70,79	50,85	38,39	27,94
	3.	0,58	18,34	83,09	84,93	74,77	54,17	34,98	25,55
	Avg	0,23	10,11	70,17	81,51	61,69	49,96	38,38	28,65
	SD	0,31	11,64	18,27	4,85	19,30	4,72	3,40	3,51

* rejected values

ScdA		Time [min]							
		0	1	5	15	30	60	90	120
Strand	Data set	**Polymerase activity [%]**							
dU0	1.	1,97	4,81	2,06	2,13	0,94	1,56	2,90	2,70
	2.	1,08	2,95	3,99	3,08	2,60	4,10	3,37	4,16
	3.	0,69	3,09	3,38	1,71	3,29	2,30	2,61	1,30
	Avg	1,25	3,62	3,14	2,31	2,28	2,65	2,96	2,72
	SD	0,66	1,03	0,98	0,70	1,21	1,30	0,39	1,43
dU-5	1.	0,00	1,91	18,13	47,68	77,08	90,06	53,14*	92,35
	2.	0,48	6,48	25,07	53,47	58,24	80,68	79,05	80,11
	3.	0,00	4,14	19,74	54,06	77,85	87,82	90,83	88,50
	Avg	0,16	4,18	20,98	51,74	71,06	86,19	84,94	86,98
	SD	0,28	2,29	3,63	3,52	11,11	4,90	8,33	6,26
dU -5/+5	1.	0,00	2,95	9,10	15,97	18,14	28,90	32,58*	29,20*
	2.	0,09	9,90	16,87	26,83	36,87	37,96	50,82	52,91
	3.	0,00	5,30	9,10	16,40	27,82	36,28	48,68	47,83
	Avg	0,03	6,05	11,69	19,73	27,61	34,38	49,75	50,37
	SD	0,05	3,54	4,49	6,15	9,37	4,82	1,52	3,59
dU+5	1.	0,90	0,66	7,01	12,25	16,44	22,54	35,91	50,84
	2.	0,14	0,06	9,95	11,18	16,12	29,77	44,04	44,01
	3.	4,80	5,74	7,92	9,10	16,90	32,20	45,50	53,03
	Avg	1,95	2,15	8,29	10,84	16,49	28,17	41,82	49,29
	SD	2,50	3,12	1,51	1,60	0,39	5,03	5,16	4,71
dU-1	1.	1,06	3,43	24,19	41,92	47,66	34,09	38,42	35,40
	2.	0,36	3,63	25,39	42,84	58,12	51,39	42,99	35,25
	3.	1,44	6,77	21,12	36,07	50,66	52,75	37,62	31,23
	Avg	0,95	4,61	23,57	40,28	52,15	46,08	39,68	33,96
	SD	0,55	1,87	2,20	3,67	5,39	10,40	2,90	2,37
dU-4	1.	1,64	14,47	8,60	37,30	44,95	67,12	56,27	61,58
	2.	0,00	0,63	14,22	42,43	55,92	66,02	58,87	67,97
	3.	0,53	1,49	16,22	31,55	58,39	72,86	37,69	55,82
	Avg	0,72	5,53	13,02	37,09	53,09	68,67	50,95	61,79
	SD	0,84	7,75	3,95	5,44	7,16	3,68	11,55	6,08
dU+1	1.	1,85	3,94	8,90	6,35	4,25	1,43	1,43	0,20
	2.	0,60	4,17	3,46	4,24	3,43	1,69	0,47	0,06
	3.	0,52	4,35	4,78	2,28	1,52	1,27	0,61	0,42
	Avg	0,99	4,15	5,71	4,29	3,07	1,46	0,84	0,23
	SD	0,74	0,21	2,84	2,04	1,40	0,21	0,52	0,18
dU+4	1.	9,38	6,35	6,98	9,72	15,29	28,44	39,45	43,23
	2.	2,55	2,47	4,32	4,47	14,33	26,67	34,15	45,43
	3.	5,98	6,87	6,30	8,76	12,00	24,31	35,03	43,26
	Avg	5,97	5,23	5,87	7,65	13,87	26,47	36,21	43,97
	SD	3,41	2,41	1,38	2,80	1,69	2,07	2,84	1,27

* rejected values

A.

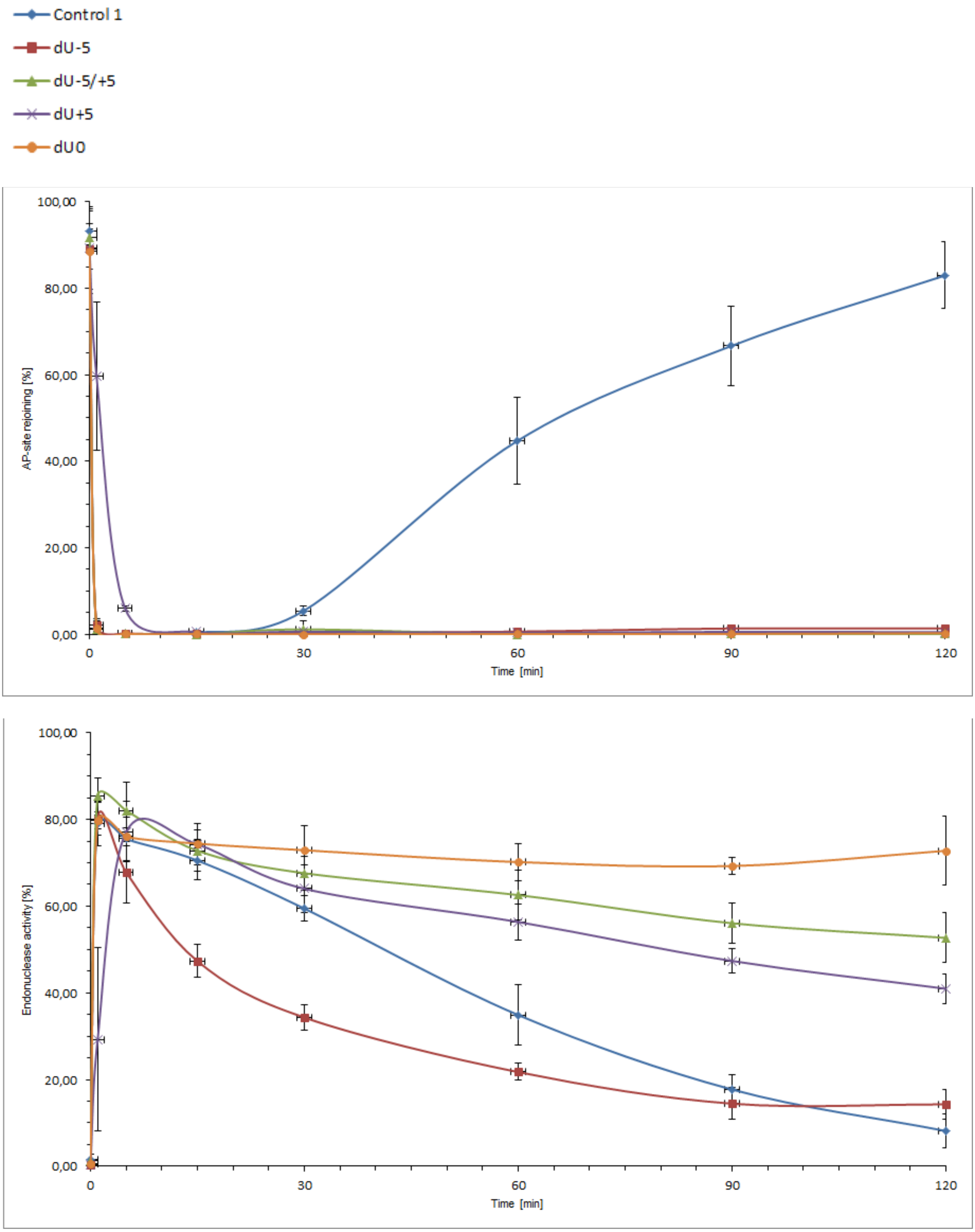

Control 1
dU-5
dU-5/+5
dU+5
dU0
100,00
80,00
60,00
40,00
20,00
0,00
AP-site rejoining [%]
0
30
60
90
120
Time [min]
100,00
80,00
60,00
40,00
20,00
0,00
Endonuclease activity [%]
0
30
60
90
120
Time [min]

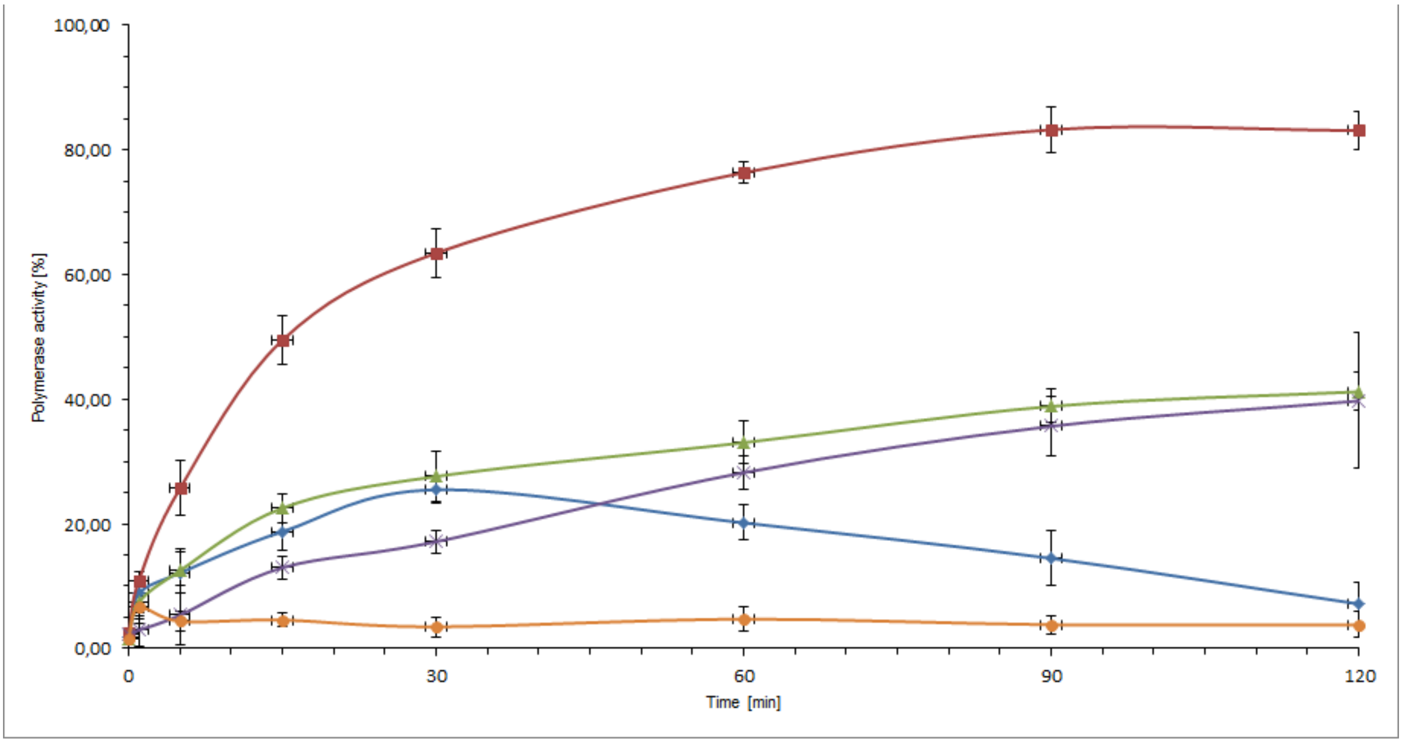

100,00
80,00
60,00
40,00
20,00
0,00
Polymerase activity [%]
0
30
60
90
120
Time [min]

B.

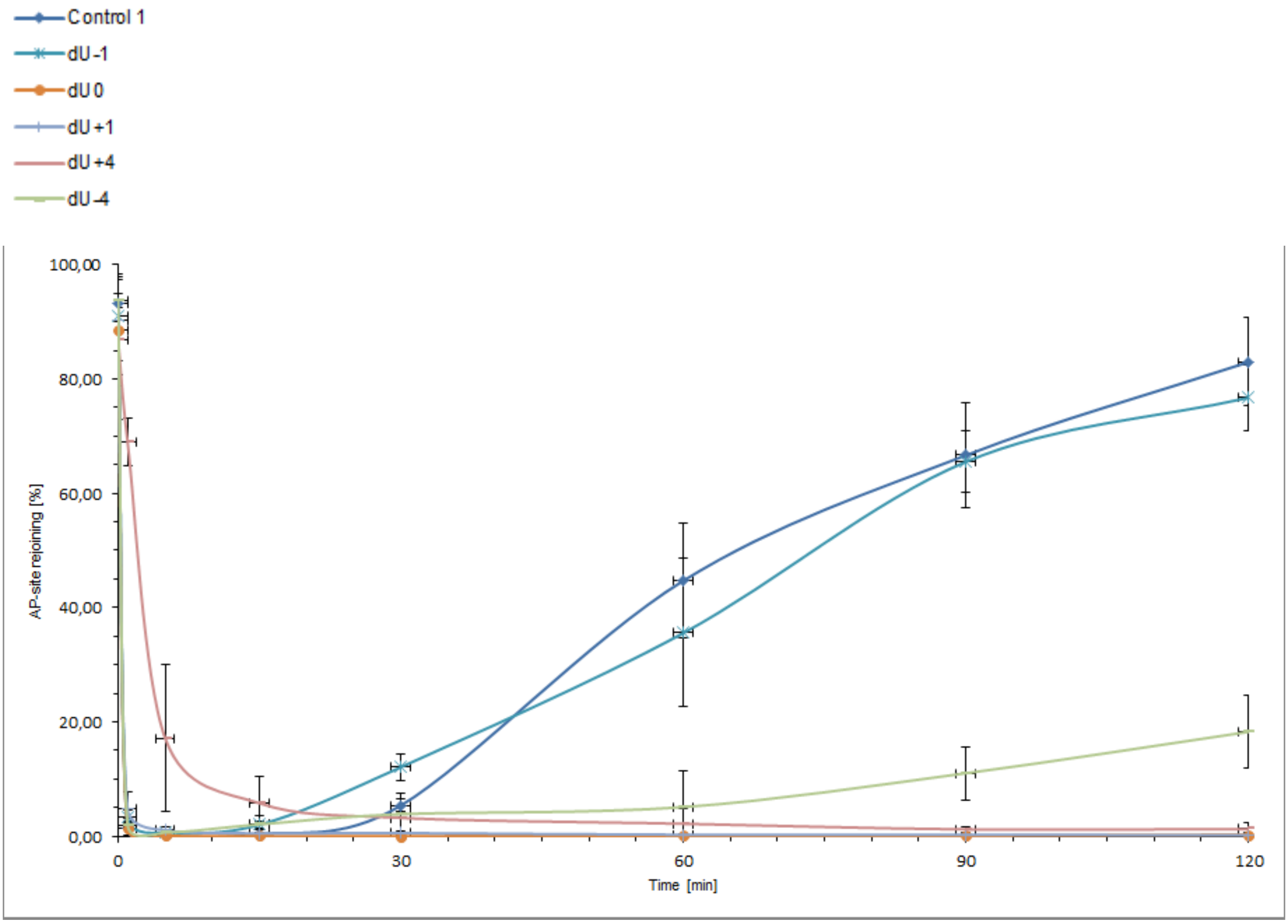

Control 1
dU -1
dU 0
dU +1
dU +4
dU -4
100,00
80,00
60,00
40,00
20,00
0,00
AP-site rejoining [%]
0
30
60
90
120
Time [min]

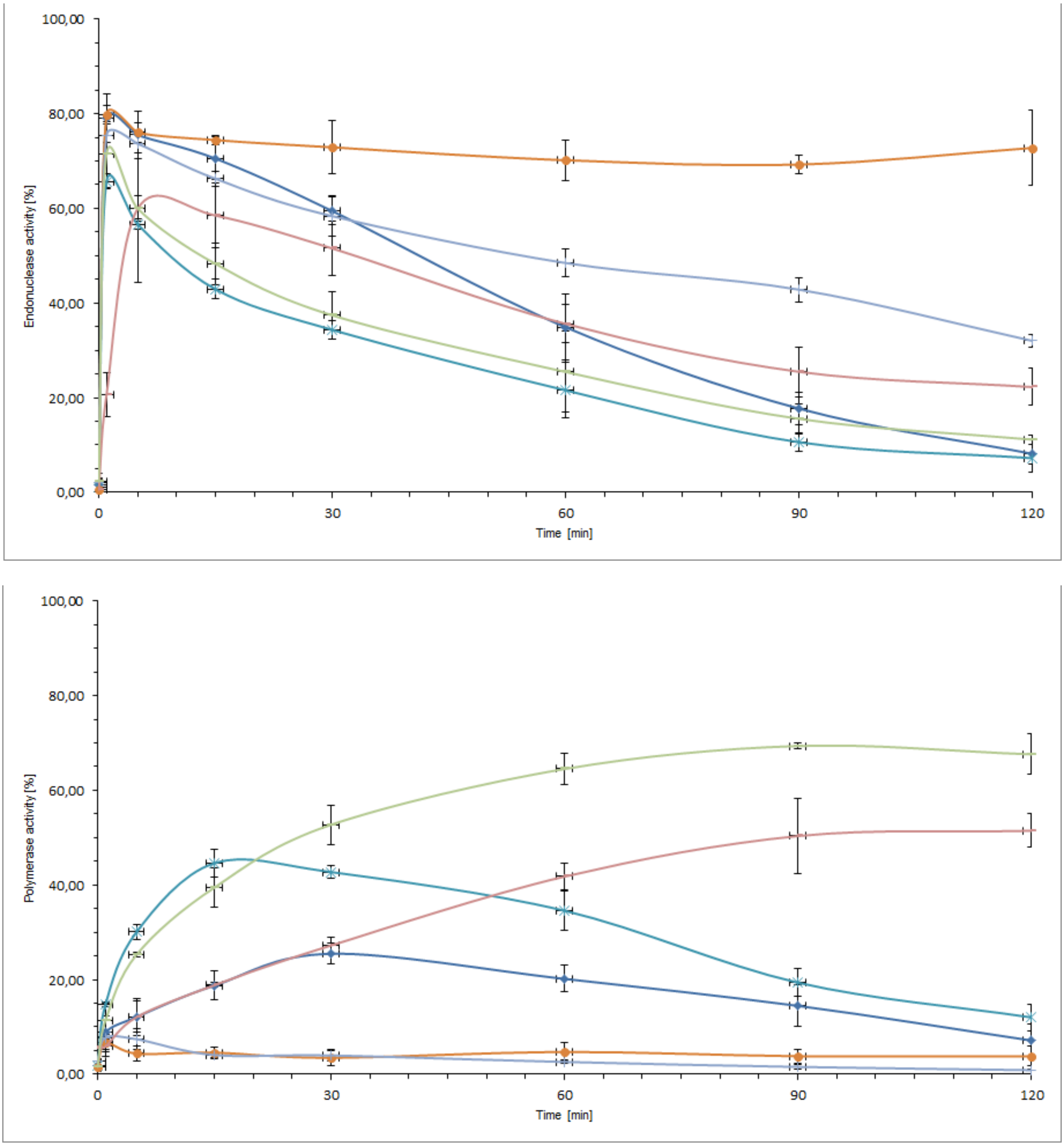

Figure S11. Graphical representation of DNA repair assays' results for RcdA – BJ Nuclear Extract. **(A)** ss-CDL, **(B)** ds-CDL

Table S6. Raw numerical data – RcdA – BJ Nuclear Extract.

RcdA		Time [min]							
		0	1	5	15	30	60	90	120
Strand	Data set	**AP site rejoining [%]**							
dU0	1.	78,56	0,66	0,10	0,05	0,03	0,03	0,04	0,07
	2.	89,23	3,38	0,22	0,05	0,04	0,10	0,05	0,56
	3.	98,10	0,44	0,10	0,00	0,00	0,04	0,09	0,09
	Avg	88,63	1,49	0,14	0,03	0,03	0,06	0,06	0,24
	SD	9,78	1,64	0,07	0,03	0,02	0,04	0,02	0,28
dU-5	1.	84,34	1,29	0,12	0,08	0,14	0,22	0,57	0,95
	2.	88,98	3,76	0,37	0,27	0,32	0,62	1,23	1,41
	3.	93,76	1,23	0,31	0,08	0,41	1,00	2,22	1,66
	Avg	89,03	2,10	0,27	0,15	0,29	0,61	1,34	1,34
	SD	4,71	1,44	0,13	0,11	0,14	0,39	0,83	0,36
dU -5/+5	1.	88,41	0,30	0,06	0,00	3,33	0,02	0,01	0,01
	2.	93,26	2,84	0,10	0,03	0,03	0,00	0,03	0,08
	3.	93,88	0,53	0,04	0,00	0,00	0,00	0,05	0,03
	Avg	91,85	1,22	0,07	0,01	1,12	0,01	0,03	0,04
	SD	2,99	1,41	0,03	0,01	1,92	0,01	0,02	0,04
dU+5	1.	78,73	65,11	5,78	0,91	1,00	0,21	0,17	0,23
	2.	91,69	73,33	5,47	0,41	0,18	0,16	0,48	0,78
	3.	97,29	40,41	6,65	0,28	19,62*	0,65	1,01	0,48
	Avg	89,23	59,62	5,97	0,53	0,59	0,34	0,56	0,50
	SD	9,52	17,13	0,61	0,33	0,58	0,27	0,42	0,27
dU-1	1.	79,04	3,49	0,57	1,88	13,11	21,38	59,68	70,16
	2.	96,29	1,98	0,82	1,53	9,40	38,38	66,52	80,79
	3.	98,06	4,30	0,66	2,58	13,74	46,89	70,34	79,20
	Avg	91,13	3,26	0,68	2,00	12,08	35,55	65,51	76,72
	SD	10,51	1,18	0,13	0,53	2,35	12,99	5,40	5,73
dU-4	1.	94,86	2,19	0,73	1,18	3,43	1,48	8,28	14,50
	2.	93,90	2,07	0,71	1,17	3,63	1,55	8,37	14,58
	3.	92,43	1,59	0,54	3,86	4,48	12,25	16,41	25,69
	Avg	93,73	1,95	0,66	2,07	3,85	5,09	11,02	18,26
	SD	1,23	0,32	0,11	1,55	0,55	6,20	4,67	6,44
dU+1	1.	82,31	8,27	1,69	0,63	0,73	0,28	0,22	0,30
	2.	96,17	2,57	0,73	0,58	21,85*	16,90*	0,33	0,08
	3.	92,40	3,45	0,84	0,31	0,37	0,24	0,13	0,22
	Avg	90,30	4,76	1,09	0,51	0,55	0,26	0,23	0,20
	SD	7,17	3,07	0,53	0,18	0,26	0,03	0,10	0,11
dU+4	1.	70,64	65,04	31,89	10,55	8,26	0,96	1,69	0,73
	2.	94,72	68,42	10,48	1,07	0,76	0,49	0,81	0,57
	3.	95,10	73,44	8,91	5,72	0,60	5,11	1,08	2,49
	Avg	86,82	68,97	17,10	5,78	3,21	2,18	1,19	1,26
	SD	14,02	4,22	12,84	4,74	4,38	2,54	0,45	1,06

* rejected values

RcdA		Time [min]							
		0	1	5	15	30	60	90	120
Strand	Data set	**Endonuclease activity [%]**							
dU0	1.	0,27	80,91	77,01	75,57	79,49	75,05	71,49	81,81
	2.	0,99	80,93	77,51	74,21	69,31	68,70	68,69	67,37
	3.	0,31	77,60	73,64	73,61	69,95	66,85	67,65	69,26
	Avg	0,52	79,82	76,05	74,46	72,92	70,20	69,27	72,81
	SD	0,41	1,92	2,11	1,01	5,70	4,30	1,99	7,85
dU-5	1.	0,16	75,72	61,31	46,14	35,93	22,36	12,24	13,07
	2.	0,04	82,70	75,39	51,58	36,12	23,30	18,62	18,12
	3.	0,80	82,03	66,69	44,24	30,91	19,57	12,53	11,67
	Avg	0,33	80,15	67,80	47,32	34,32	21,75	14,46	14,29
	SD	0,41	3,85	7,10	3,81	2,96	1,94	3,61	3,39
dU -5/+5	1.	0,00	90,30	85,48	75,26	69,97	60,84	56,61	52,01
	2.	0,93	82,44	86,04	75,85	69,75	69,04	60,46	58,76
	3.	2,86	83,41	74,59	67,31	63,16	57,83	51,19	47,34
	Avg	1,26	85,38	82,03	72,81	67,63	62,57	56,09	52,70
	SD	1,46	4,28	6,45	4,77	3,87	5,80	4,66	5,74
dU+5	1.	0,35	16,42	69,42	68,79	65,09	51,84	44,07	38,93
	2.	0,48	17,71	82,95	76,45	69,03	57,39	48,61	44,99
	3.	0,11	53,58	79,09	77,56	58,01	59,88	49,43	38,98
	Avg	0,31	29,24	77,15	74,27	64,04	56,37	47,37	40,97
	SD	0,19	21,10	6,97	4,77	5,58	4,12	2,89	3,48
dU-1	1.	0,48	66,42	56,59	41,03	33,92	27,34	11,74	8,66
	2.	2,09	63,82	55,59	45,08	36,57	21,62	11,63	6,26
	3.	0,66	66,75	57,75	42,70	32,56	15,79	8,48	6,79
	Avg	1,07	65,66	56,64	42,93	34,35	21,59	10,62	7,24
	SD	0,88	1,61	1,08	2,04	2,04	5,77	1,85	1,26
dU-4	1.	1,53	69,47	59,98	45,80	39,83	29,06	15,14	10,83
	2.	2,17	65,67	57,23	45,68	40,79	31,58	18,93	12,28
	3.	3,06	79,19	62,78	53,44	31,65	15,76	12,57	10,36
	Avg	2,25	71,44	60,00	48,31	37,42	25,47	15,55	11,15
	SD	0,77	6,97	2,78	4,45	5,02	8,50	3,20	1,00
dU+1	1.	0,61	71,16	71,38	64,52	62,02	49,85	40,77	31,20
	2.	1,12	78,47	75,33	66,65	53,59	45,01	45,73	33,65
	3.	4,18	76,58	74,33	67,64	59,54	50,60	42,06	31,21
	Avg	1,97	75,41	73,68	66,27	58,38	48,49	42,85	32,02
	SD	1,93	3,79	2,06	1,59	4,33	3,03	2,57	1,41
dU+4	1.	0,33	1,57*	42,71	52,12	45,10	32,95	19,60	18,74
	2.	0,42	23,97	72,90	65,75	55,68	40,33	27,18	26,50
	3.	0,85	17,36	64,27	57,67	53,90	33,59	29,71	21,54
	Avg	0,53	20,67	59,96	58,51	51,56	35,63	25,50	22,26
	SD	0,28	4,67	15,55	6,85	5,66	4,09	5,26	3,93

* rejected values

RcdA		Time [min]							
		0	1	5	15	30	60	90	120
**Strand**	**Data set**	**Polymerase activity [%]**							
dU0	1.	2,20	5,38	2,98	3,62	1,67	2,54	2,18	2,40
	2.	1,53	5,51	3,94	5,66	4,44	6,11	4,22	2,82
	3.	1,11	8,82	6,21	4,46	4,39	5,52	5,08	6,17
	Avg	1,61	6,57	4,38	4,58	3,50	4,72	3,83	3,80
	SD	0,55	1,95	1,66	1,03	1,59	1,91	1,49	2,07
dU-5	1.	2,01	11,51	27,65	47,36	59,61	75,34	86,49	84,99
	2.	2,51	10,83	20,62	47,15	63,18	75,50	79,36	79,50
	3.	3,11	10,42	28,76	53,95	67,56	78,22	83,87	84,82
	Avg	2,54	10,92	25,68	49,49	63,45	76,35	83,24	83,10
	SD	0,55	0,55	4,41	3,87	3,98	1,62	3,61	3,12
dU -5/+5	1.	0,95	3,68	10,43	20,28	23,09	34,48	36,86	39,76
	2.	1,92	10,14	10,46	22,49	28,74	29,12	37,80	39,09
	3.	1,82	8,49	16,62	24,87	31,03	35,54	41,93	44,75
	Avg	1,56	7,44	12,50	22,55	27,62	33,05	38,86	41,20
	SD	0,53	3,36	3,56	2,30	4,08	3,44	2,70	3,09
dU+5	1.	4,49	3,62	9,52	13,44	19,00	30,48	40,51	42,29
	2.	0,03	0,02	0,02	14,54	15,44	25,30	30,84	27,77
	3.	2,61	5,23	6,51	10,94	17,02	28,88	35,64	49,11
	Avg	2,38	2,96	5,35	12,98	17,15	28,22	35,66	39,72
	SD	2,24	2,67	4,85	1,85	1,78	2,65	4,84	10,90
dU-1	1.	3,56	15,15	31,34	48,02	41,63	39,47	22,74	15,27
	2.	0,87	14,35	30,69	42,64	42,19	32,44	17,86	10,05
	3.	0,75	14,60	28,19	43,13	44,38	31,86	17,54	10,79
	Avg	1,73	14,70	30,07	44,60	42,73	34,59	19,38	12,03
	SD	1,59	0,41	1,66	2,98	1,45	4,23	2,91	2,82
dU-4	1.	1,33	15,89	25,55	42,37	50,26	62,02	69,55	69,37
	2.	1,30	9,25	25,60	41,32	50,42	63,19	69,94	70,74
	3.	2,11	8,89	24,67	34,69	57,52	68,33	68,62	62,73
	Avg	1,58	11,34	25,27	39,46	52,74	64,51	69,37	67,61
	SD	0,46	3,94	0,53	4,17	4,15	3,36	0,68	4,28
dU+1	1.	3,03	9,50	9,77	4,31	4,45	2,23	1,15	0,70
	2.	2,53	6,39	5,41	3,28	2,69	2,57	1,56	0,89
	3.	2,70	7,43	7,12	4,64	4,82	3,04	1,97	1,04
	Avg	2,75	7,77	7,43	4,07	3,98	2,61	1,56	0,88
	SD	0,25	1,58	2,20	0,71	1,14	0,41	0,41	0,17
dU+4	1.	8,27	3,64	8,13	19,01	25,64	44,43	58,73	55,51
	2.	4,72	6,11	11,98	19,19	29,04	42,22	49,45	48,92
	3.	3,84	7,82	16,15	18,24	27,02	38,71	42,87	49,96
	Avg	5,61	5,86	12,08	18,81	27,23	41,79	50,35	51,47
	SD	2,34	2,10	4,01	0,51	1,71	2,88	7,97	3,54

* rejected values

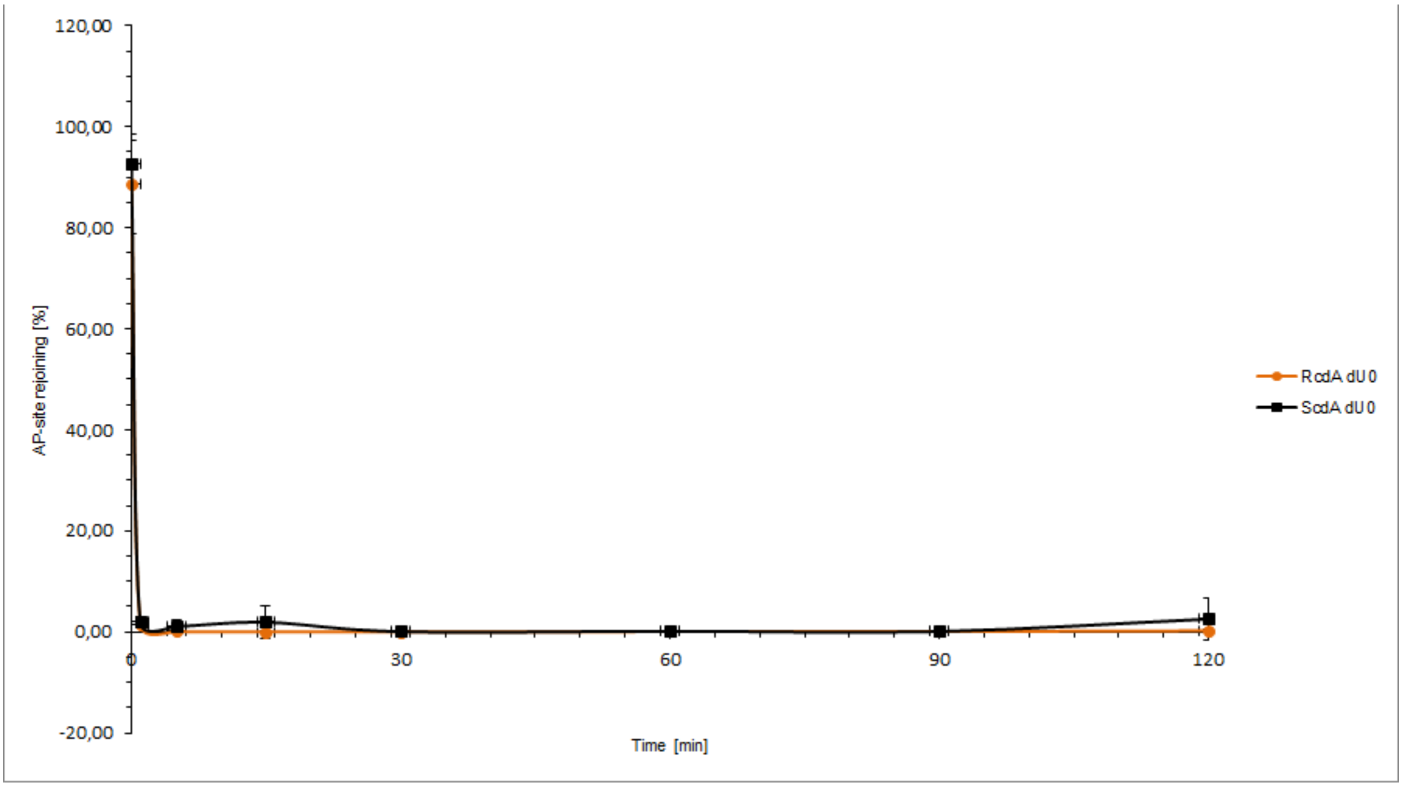
120,00
100,00
80,00
60,00
40,00
20,00
0,00
-20,00
AP-site rejoining [%]
0
30
60
90
120
Time [min]
RcdA dU 0
ScdA dU 0

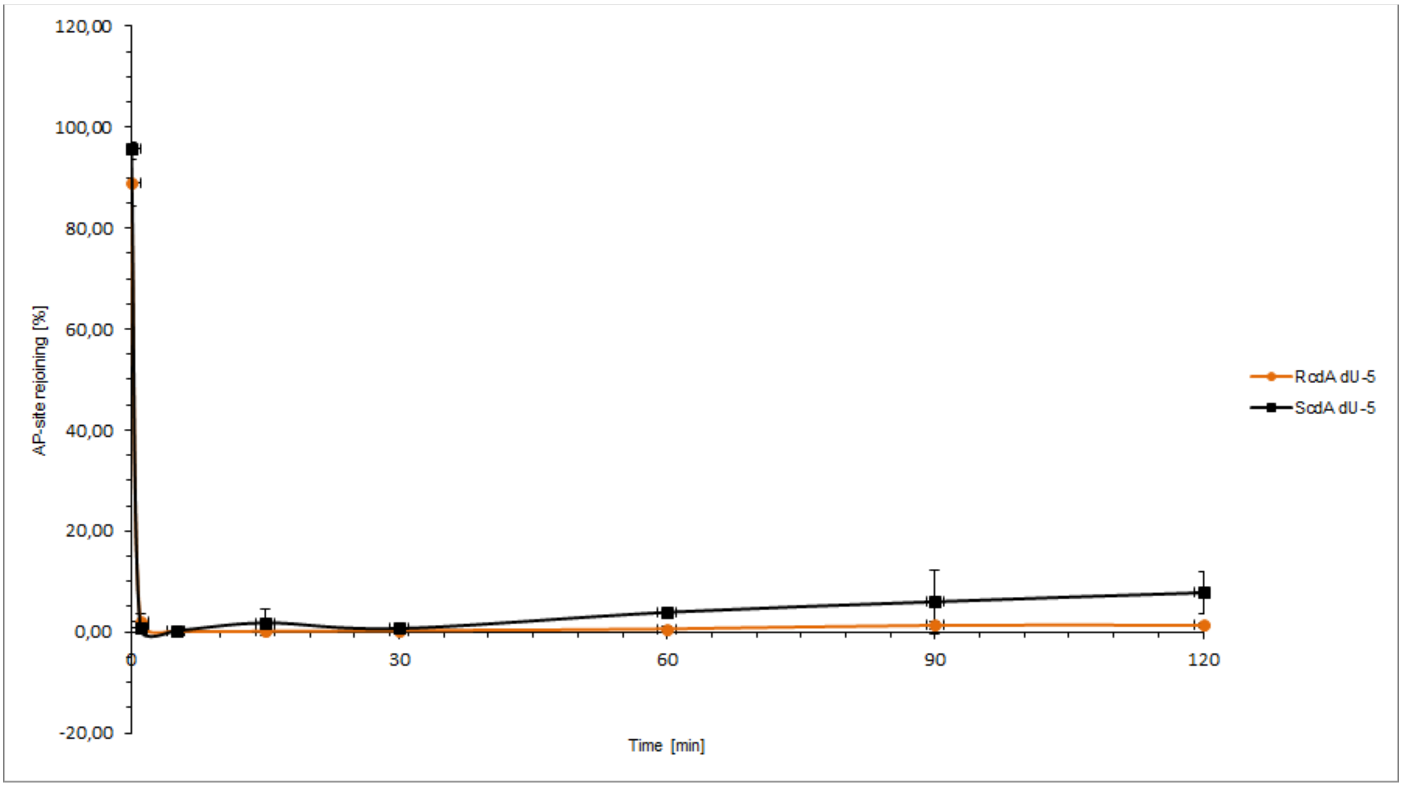
120,00
100,00
80,00
60,00
40,00
20,00
0,00
-20,00
AP-site rejoining [%]
0
30
60
90
120
Time [min]
RcdA dU-5
ScdA dU-5

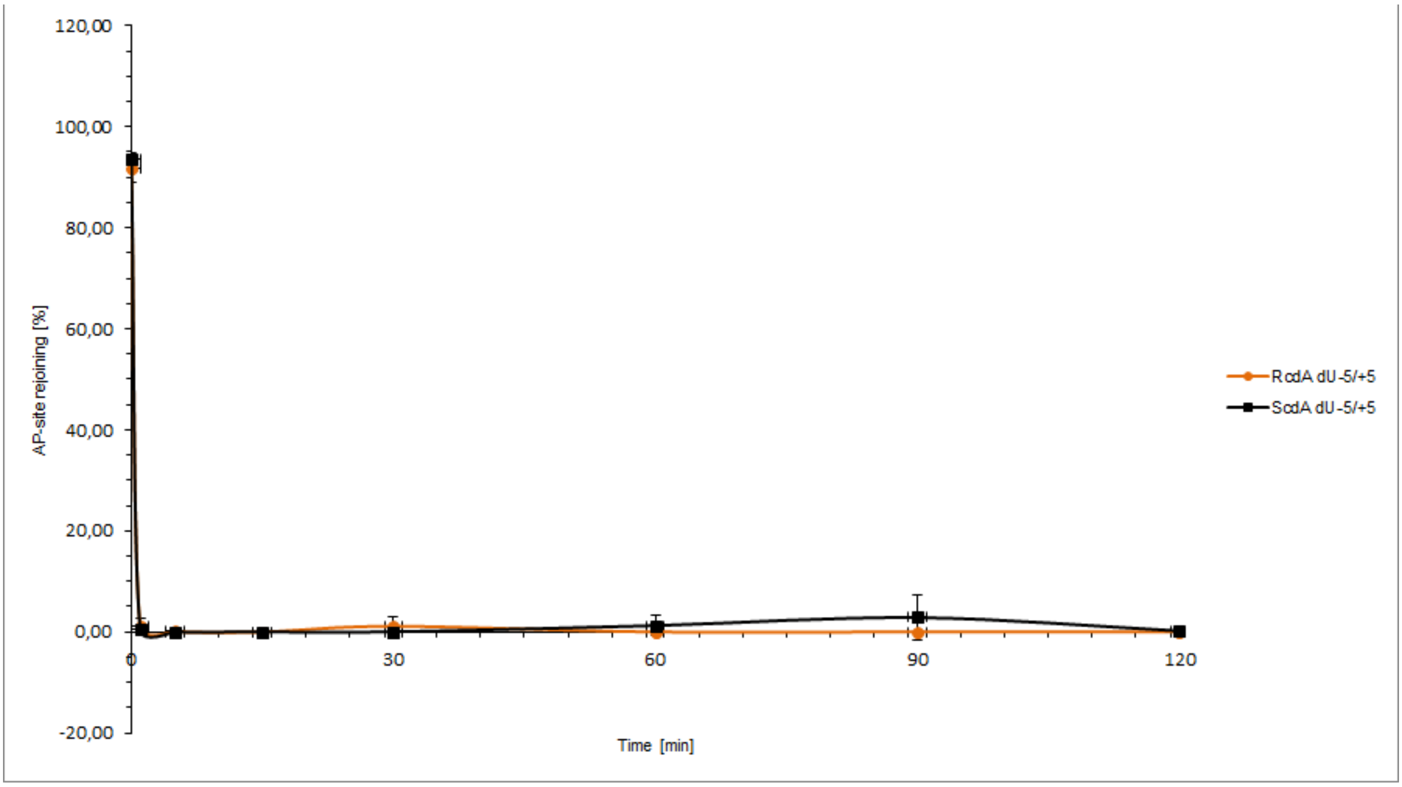
120,00
100,00
80,00
60,00
40,00
20,00
0,00
-20,00
AP-site rejoining [%]
0
30
60
90
120
Time [min]
RcdA dU -5/+5
ScdA dU -5/+5

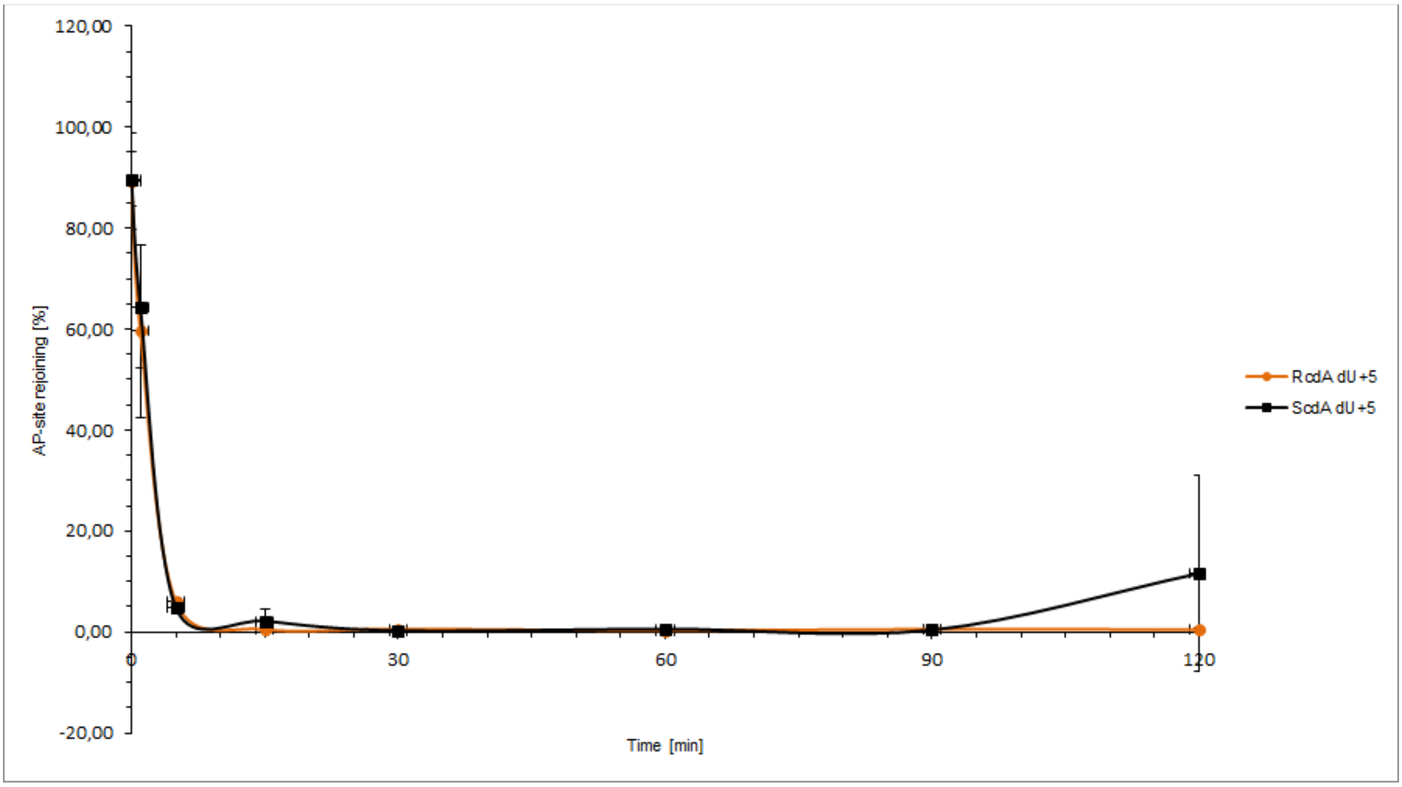
120,00
100,00
80,00
60,00
40,00
20,00
0,00
-20,00
AP-site rejoining [%]
0
30
60
90
120
Time [min]
RcdA dU +5
ScdA dU +5

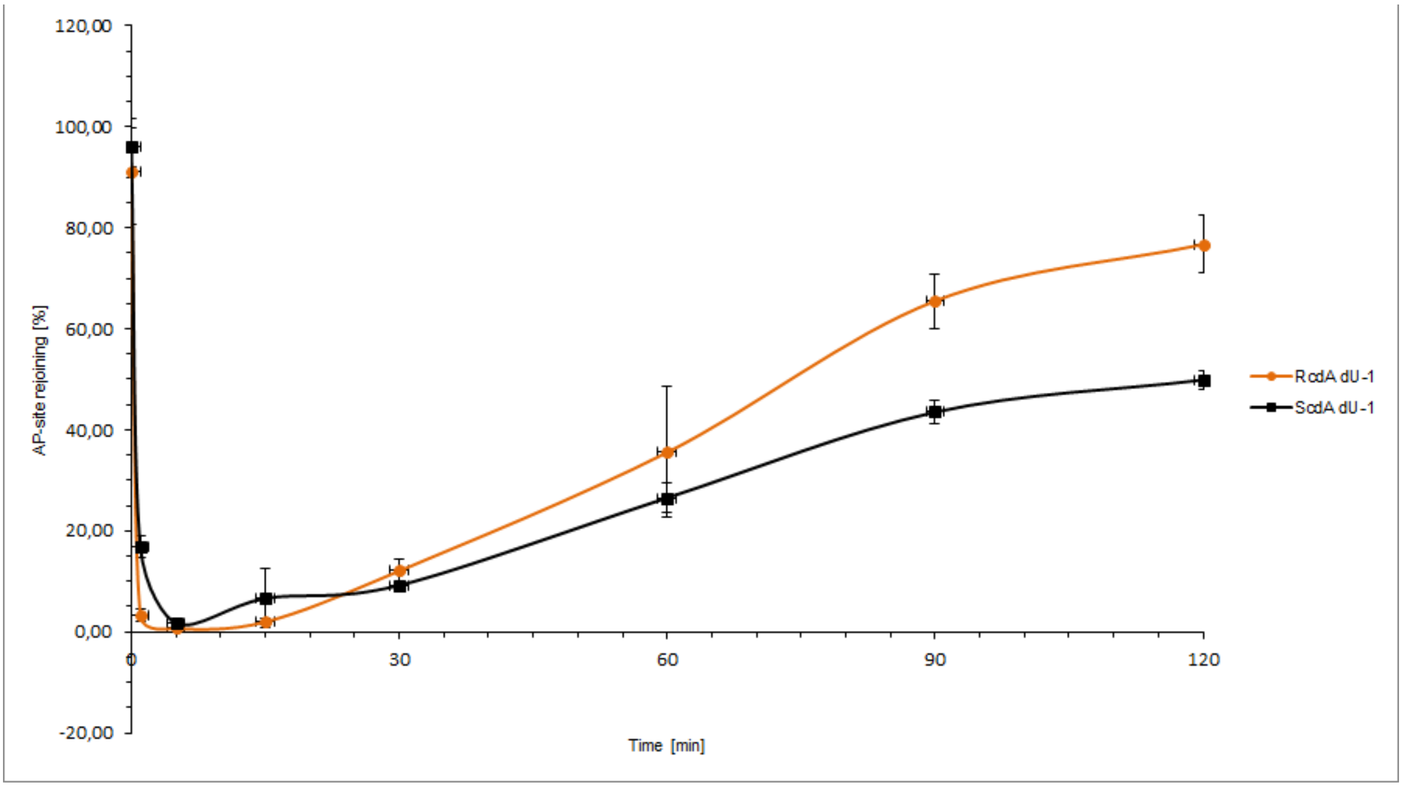

120,00
100,00
80,00
60,00
40,00
20,00
0,00
-20,00
AP-site rejoining [%]
0
30
60
90
120
Time [min]
RcdA dU-1
ScdA dU-1

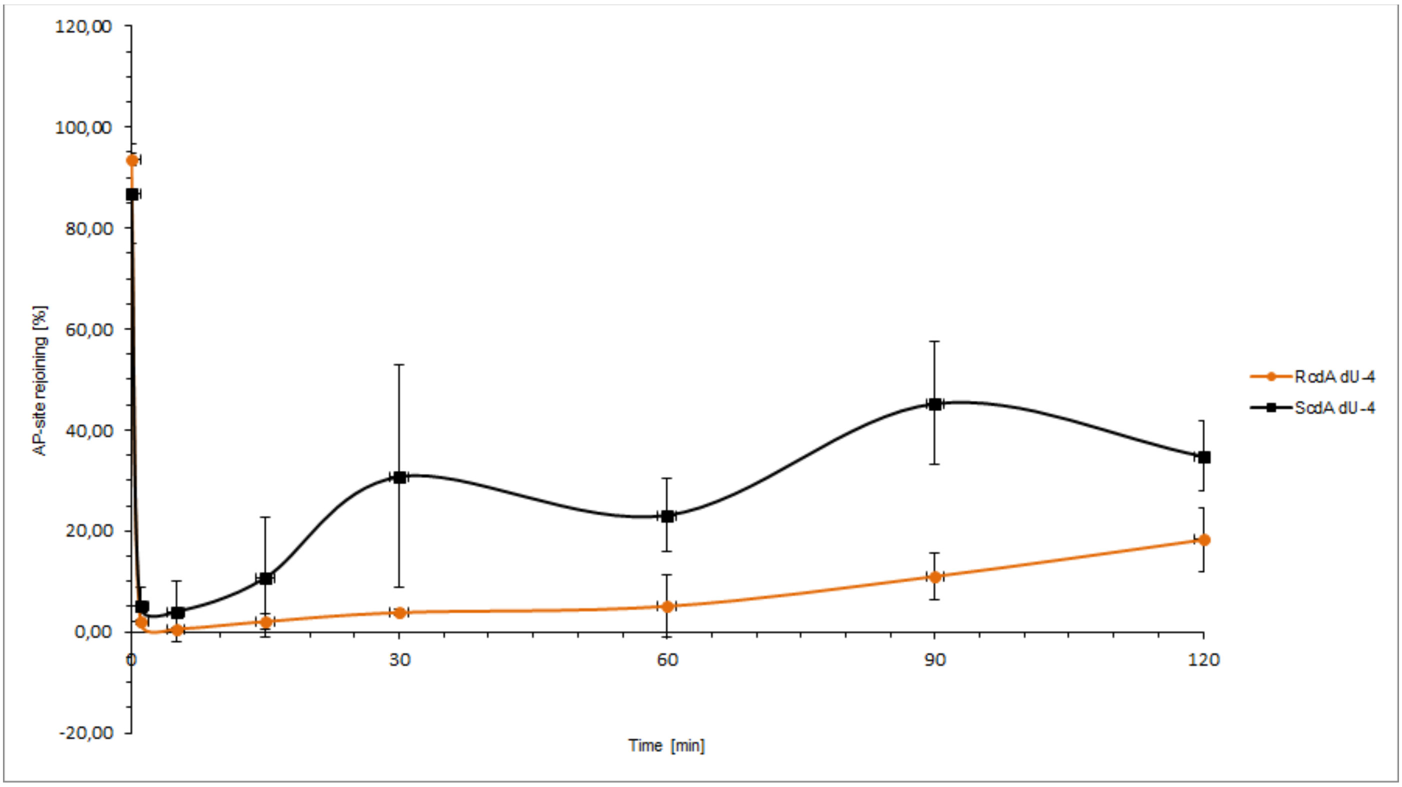

120,00
100,00
80,00
60,00
40,00
20,00
0,00
-20,00
AP-site rejoining [%]
0
30
60
90
120
Time [min]
RcdA dU-4
ScdA dU-4

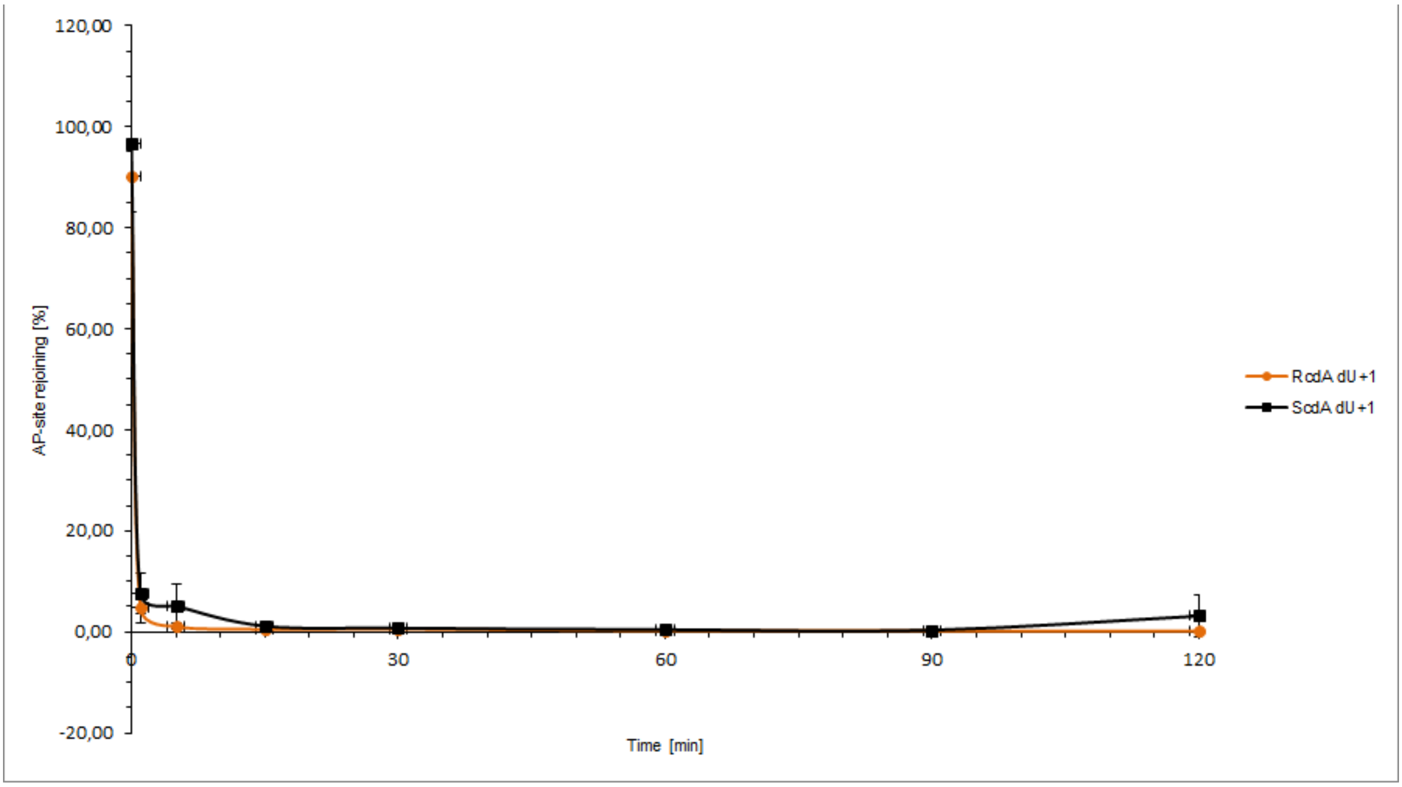

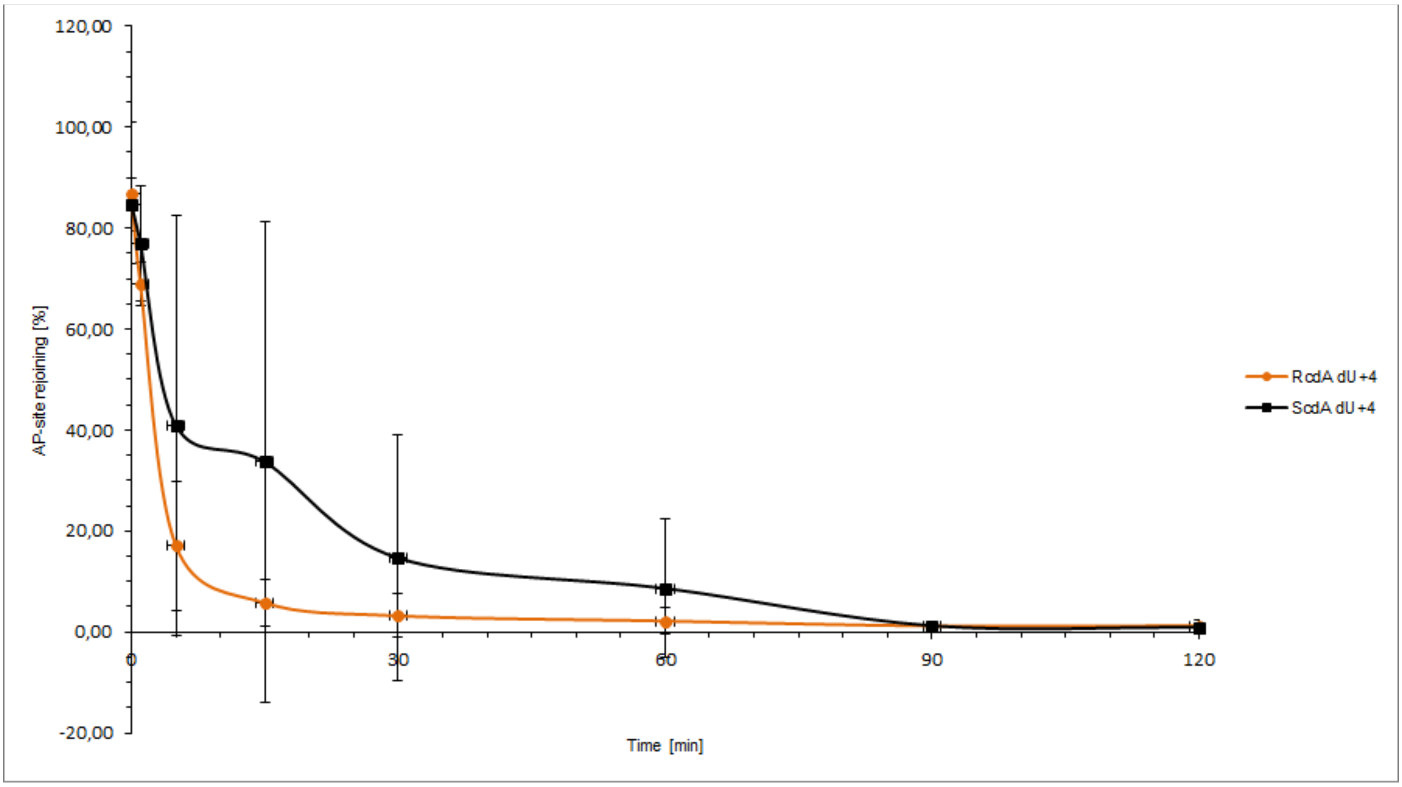

Figure S12. AP site rejoining [%] of ScdA vs. RcdA – comparison of individual strands – BJ Nuclear Extract.

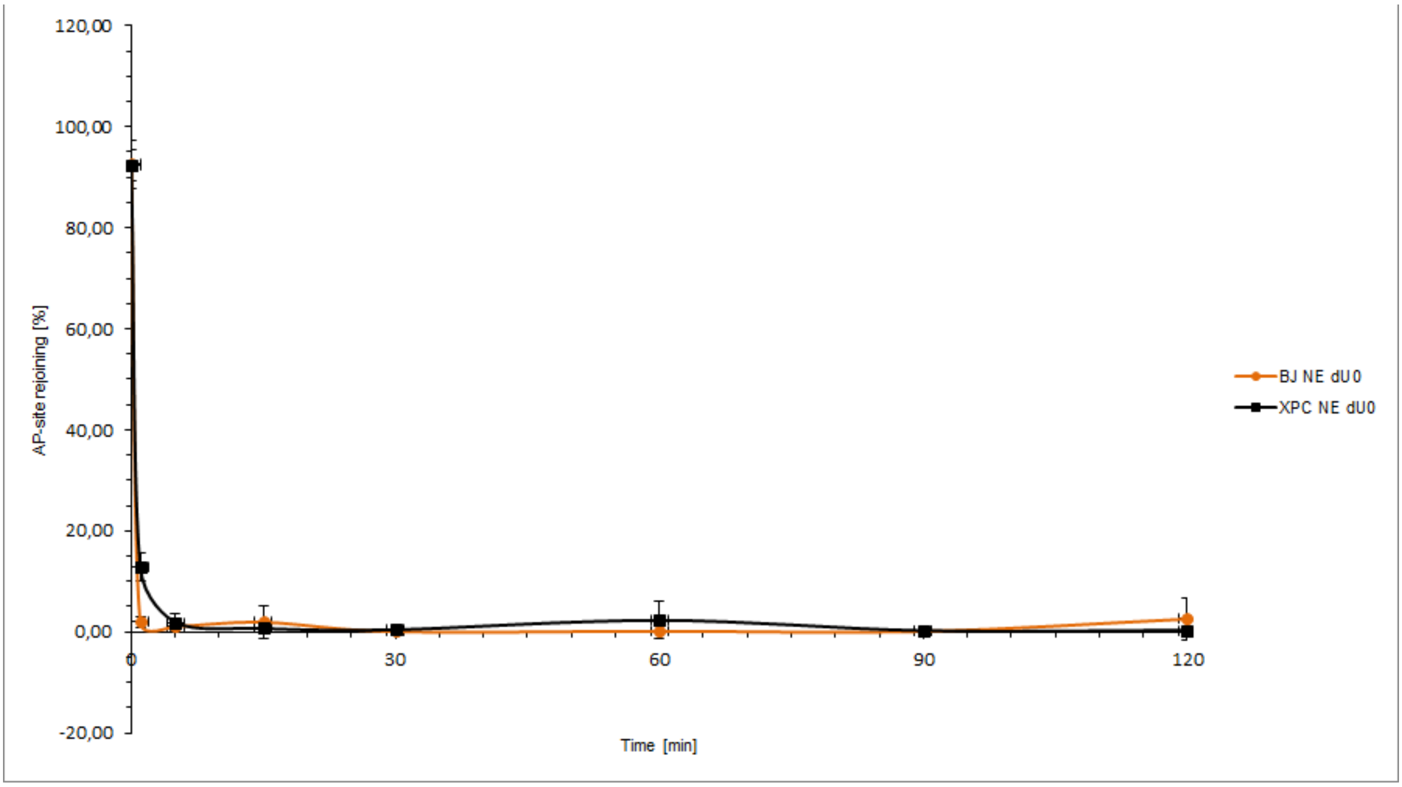
120,00
100,00
80,00
60,00
40,00
20,00
0,00
-20,00
AP-site rejoining [%]
0
30
60
90
120
Time [min]
BJ NE dU0
XPC NE dU0

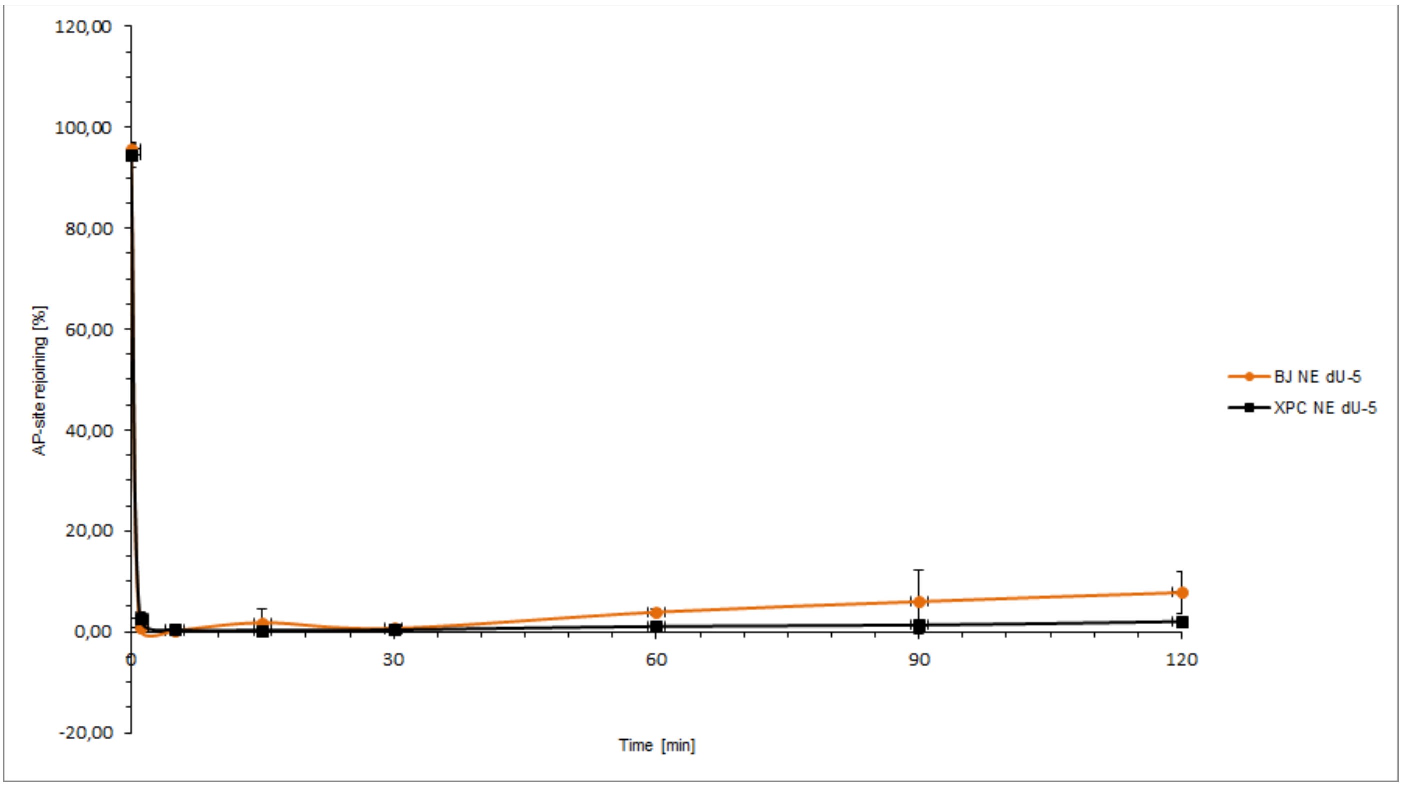
120,00
100,00
80,00
60,00
40,00
20,00
0,00
-20,00
AP-site rejoining [%]
0
30
60
90
120
Time [min]
BJ NE dU-5
XPC NE dU-5

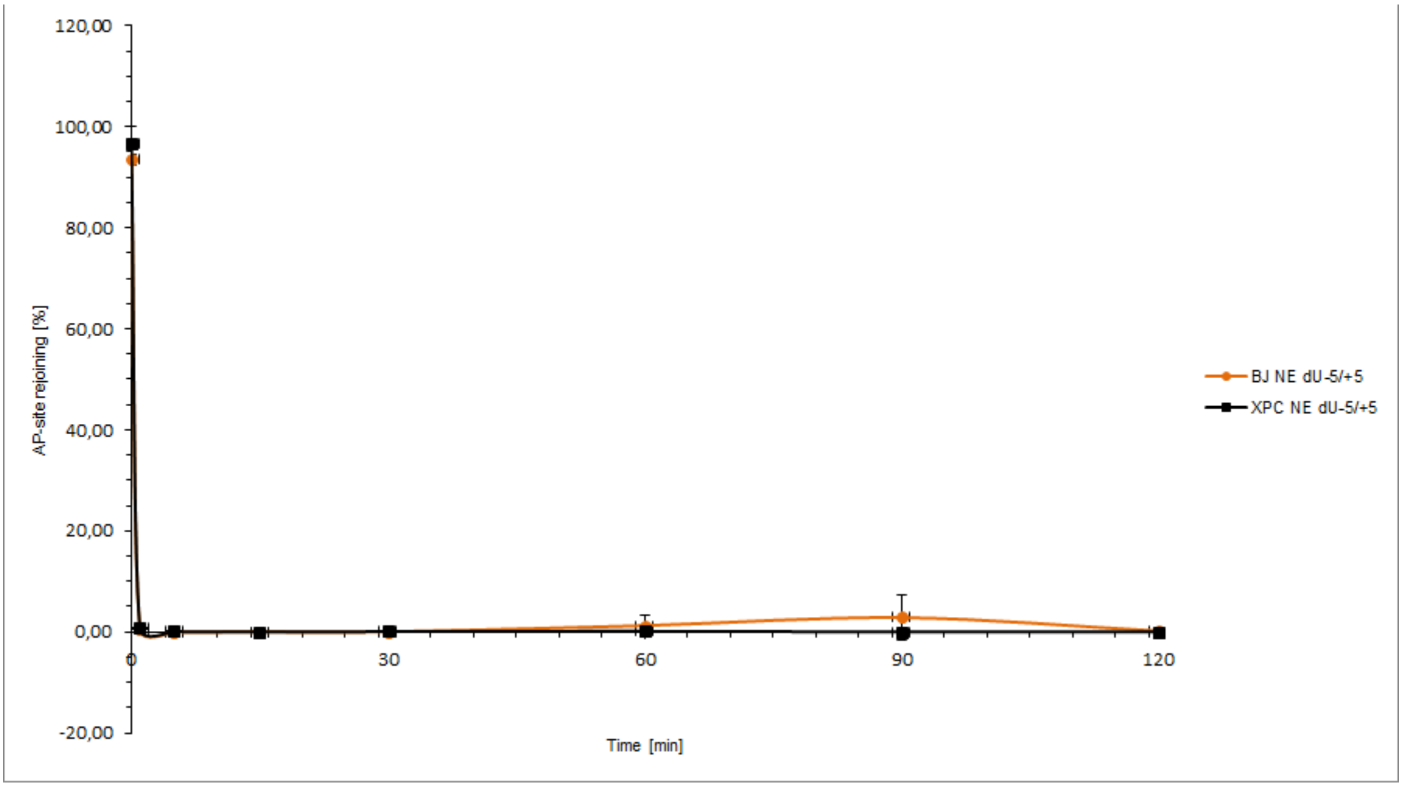

120,00
100,00
80,00
60,00
40,00
20,00
0,00
-20,00
AP-site rejoining [%]
0
30
60
90
120
Time [min]
BJ NE dU-5/+5
XPC NE dU-5/+5

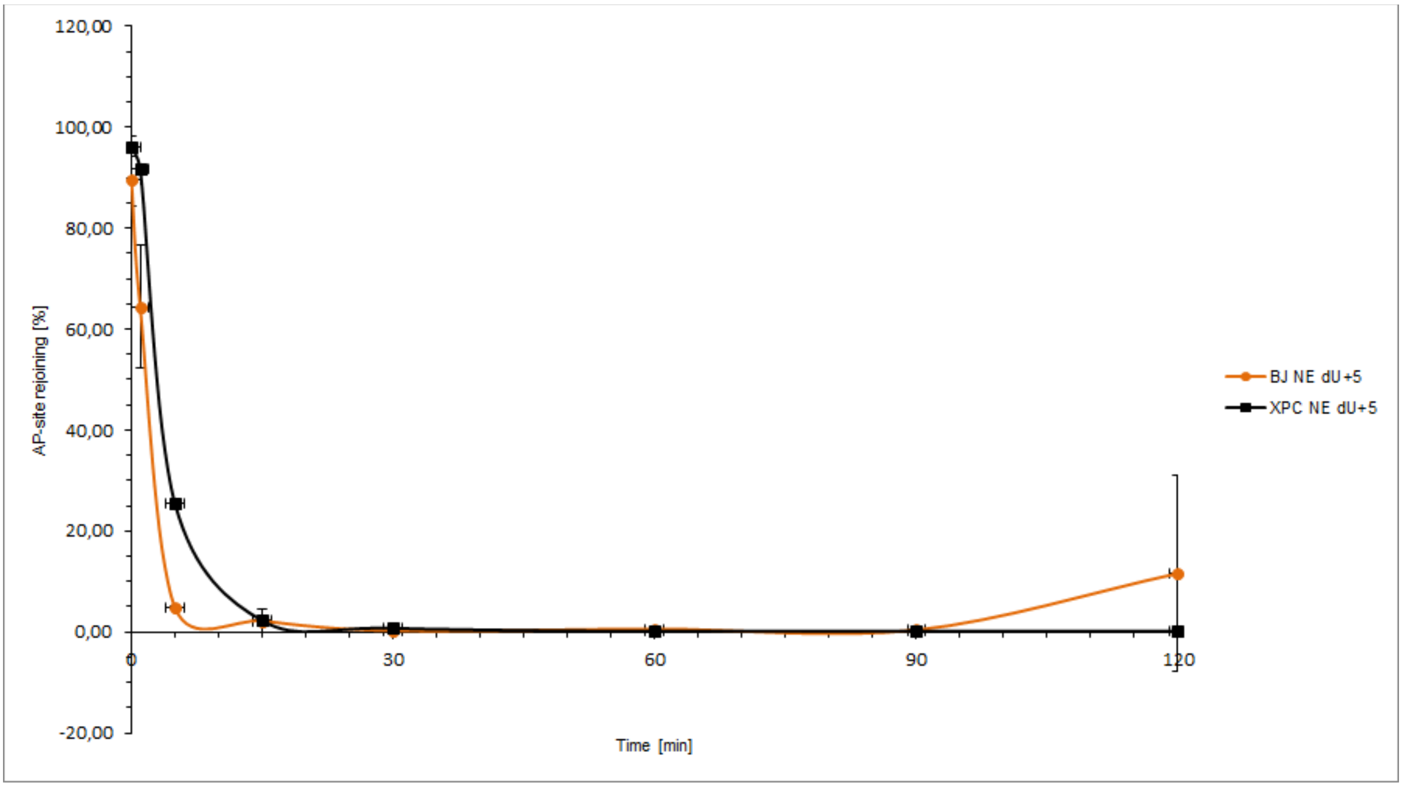

120,00
100,00
80,00
60,00
40,00
20,00
0,00
-20,00
AP-site rejoining [%]
0
30
60
90
120
Time [min]
BJ NE dU+5
XPC NE dU+5

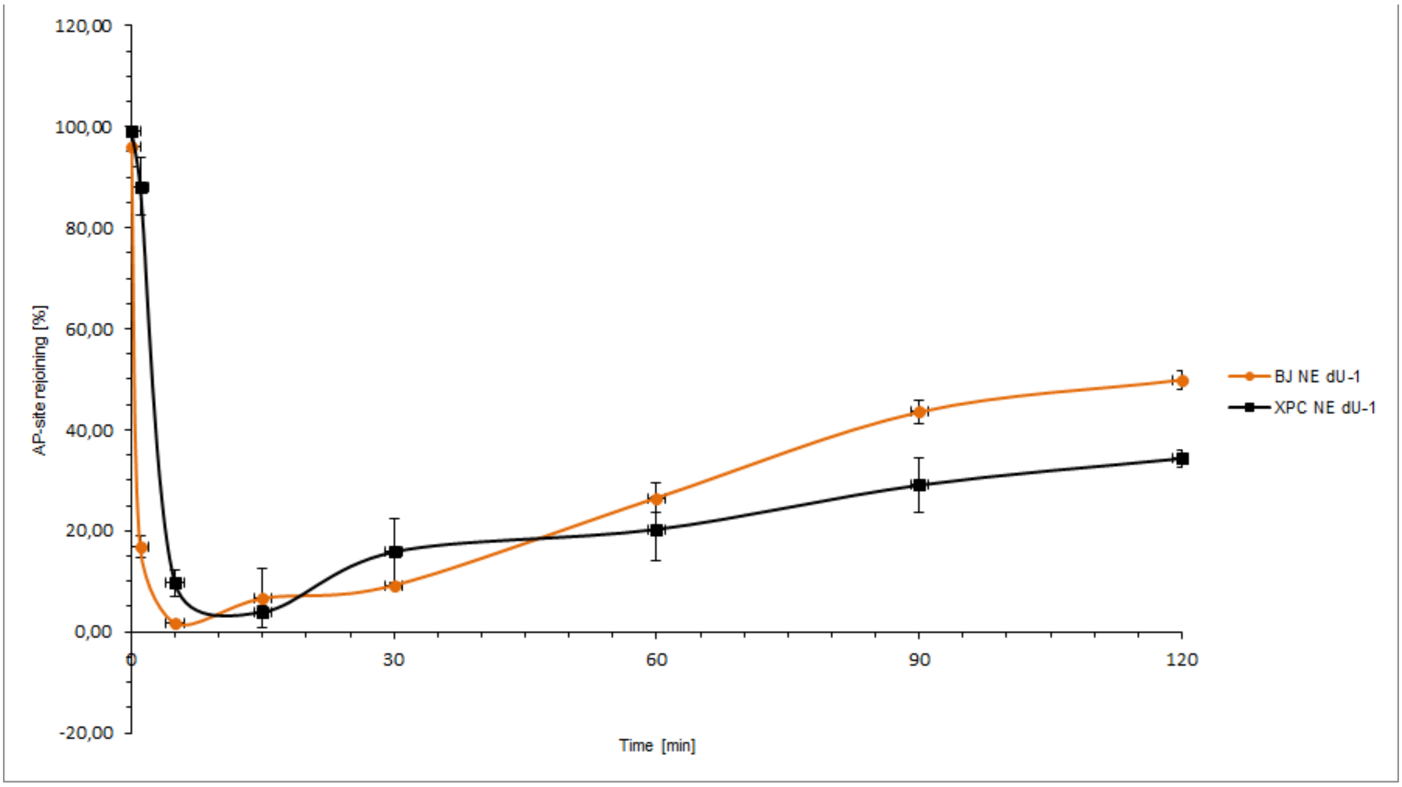

120,00
100,00
80,00
60,00
40,00
20,00
0,00
-20,00
AP-site rejoining [%]
0
30
60
90
120
Time [min]
BJ NE dU-1
XPC NE dU-1

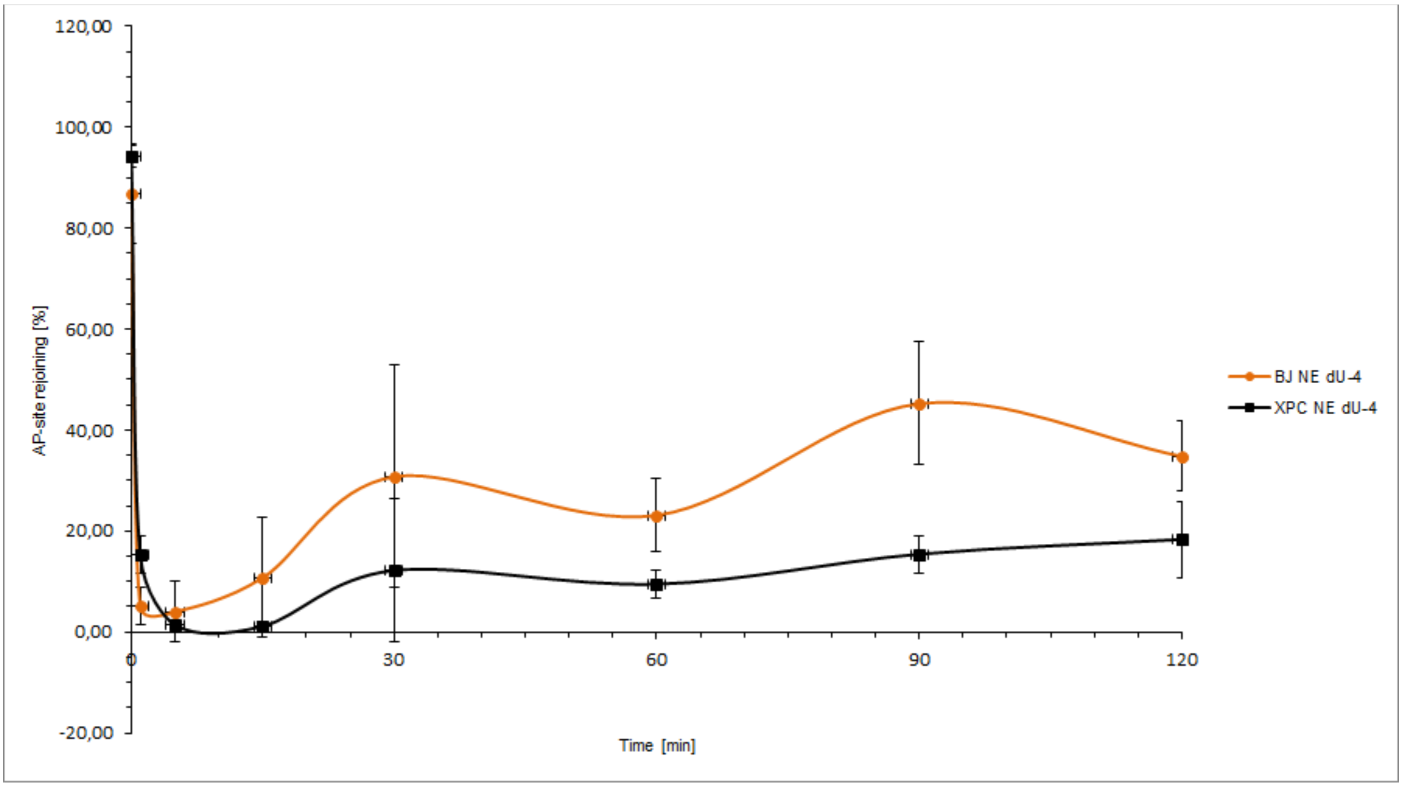

120,00
100,00
80,00
60,00
40,00
20,00
0,00
-20,00
AP-site rejoining [%]
0
30
60
90
120
Time [min]
BJ NE dU-4
XPC NE dU-4

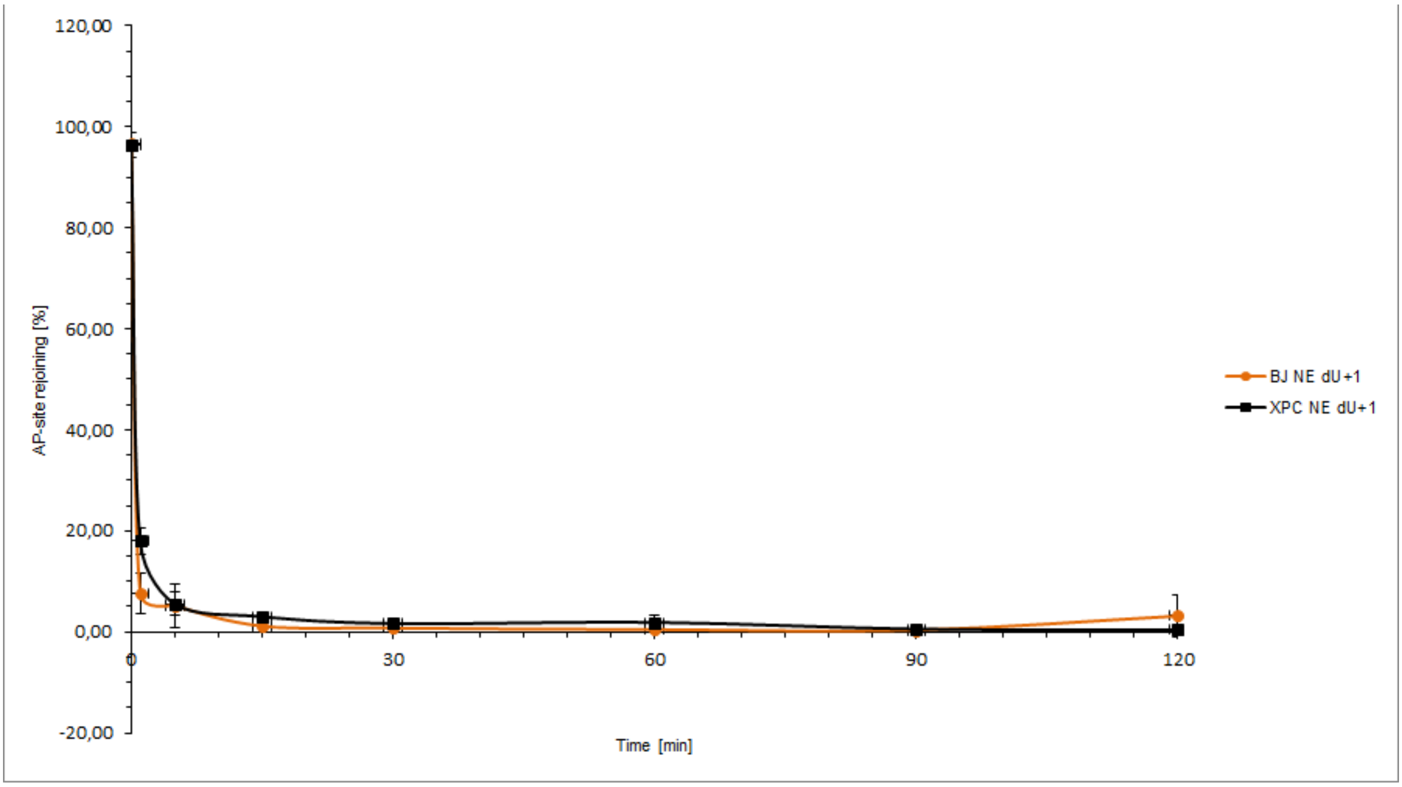

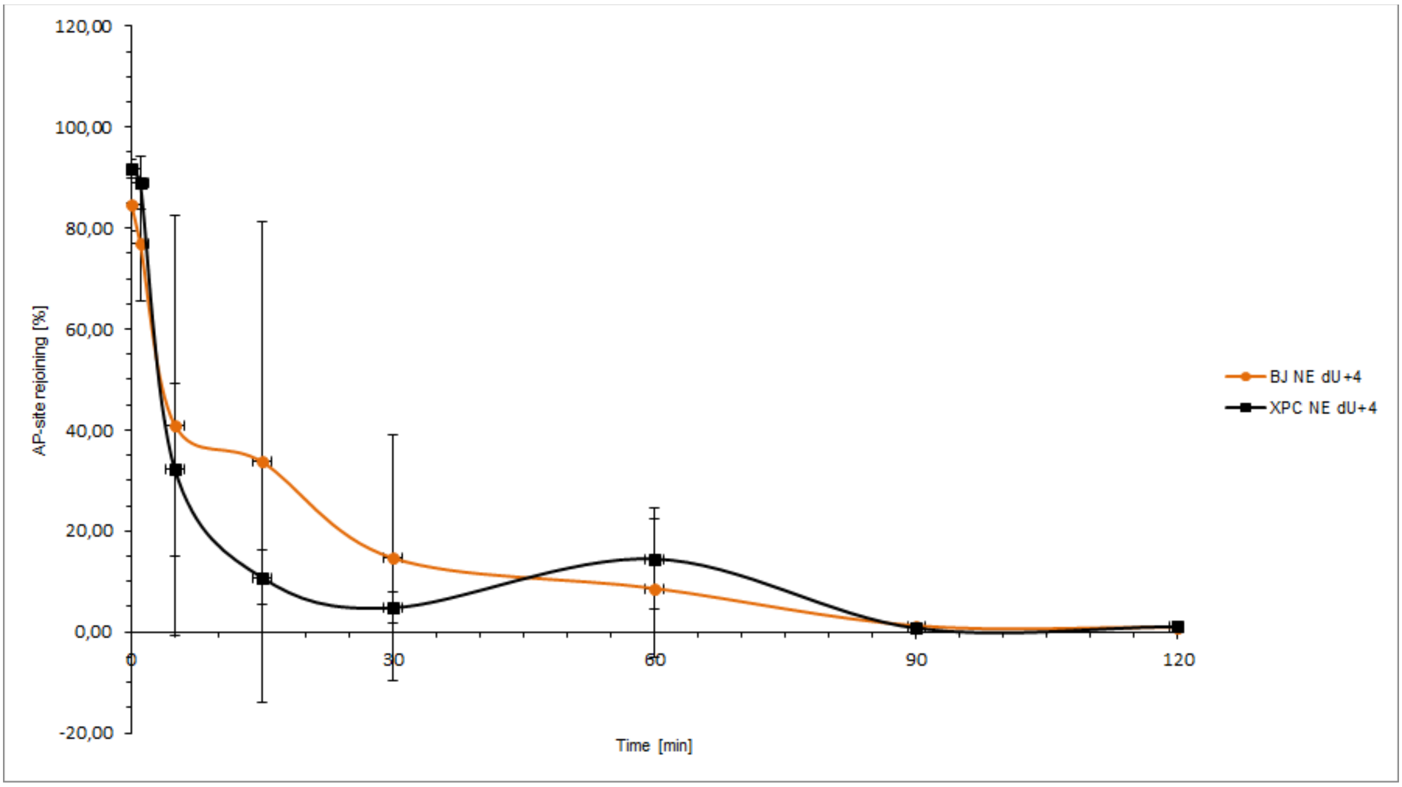

Figure S13. AP site rejoining [%] of BJ vs. XPC – comparison of individual strands – ScdA Nuclear Extract.

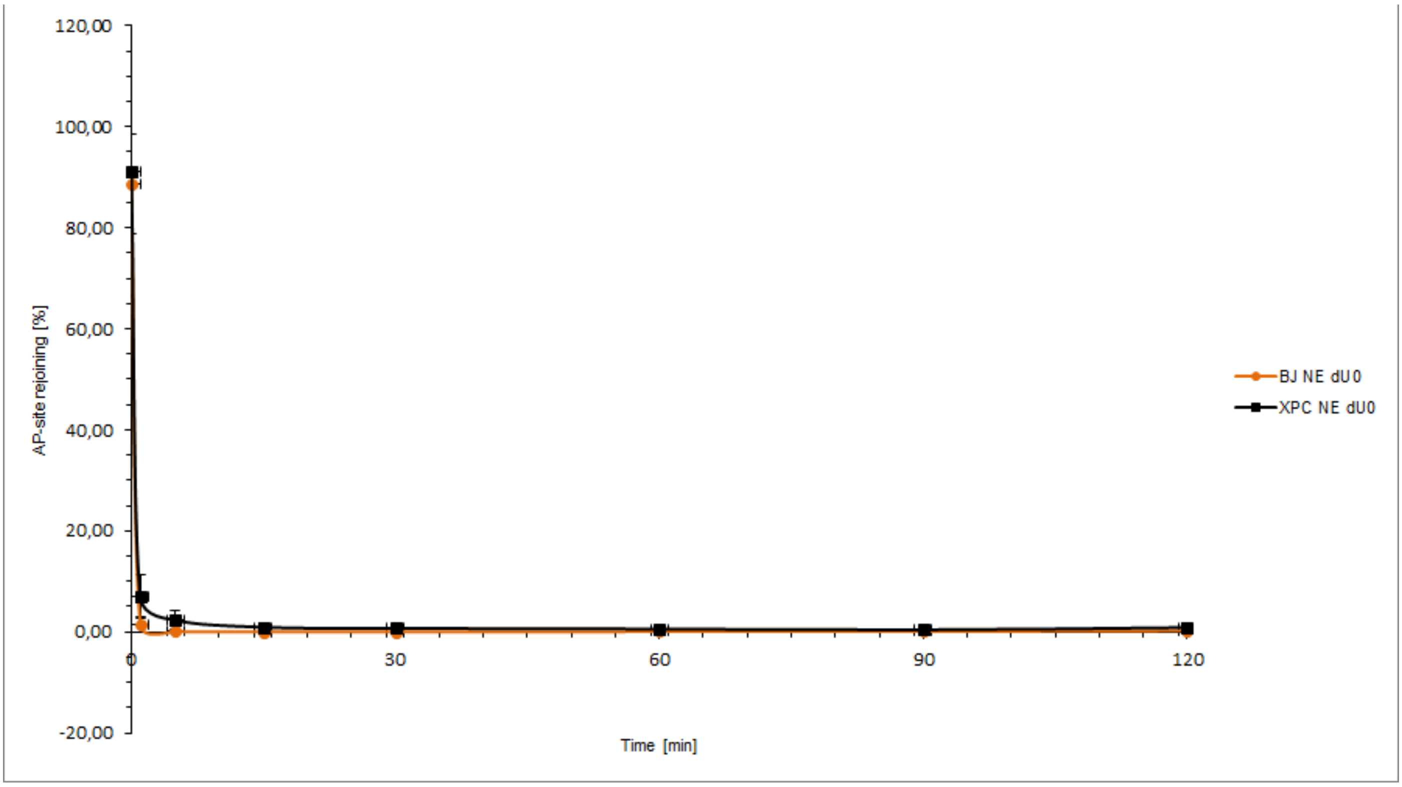

120,00
100,00
80,00
60,00
40,00
20,00
0,00
-20,00
AP-site rejoining [%]
0
30
60
90
120
Time [min]
BJ NE dU0
XPC NE dU0

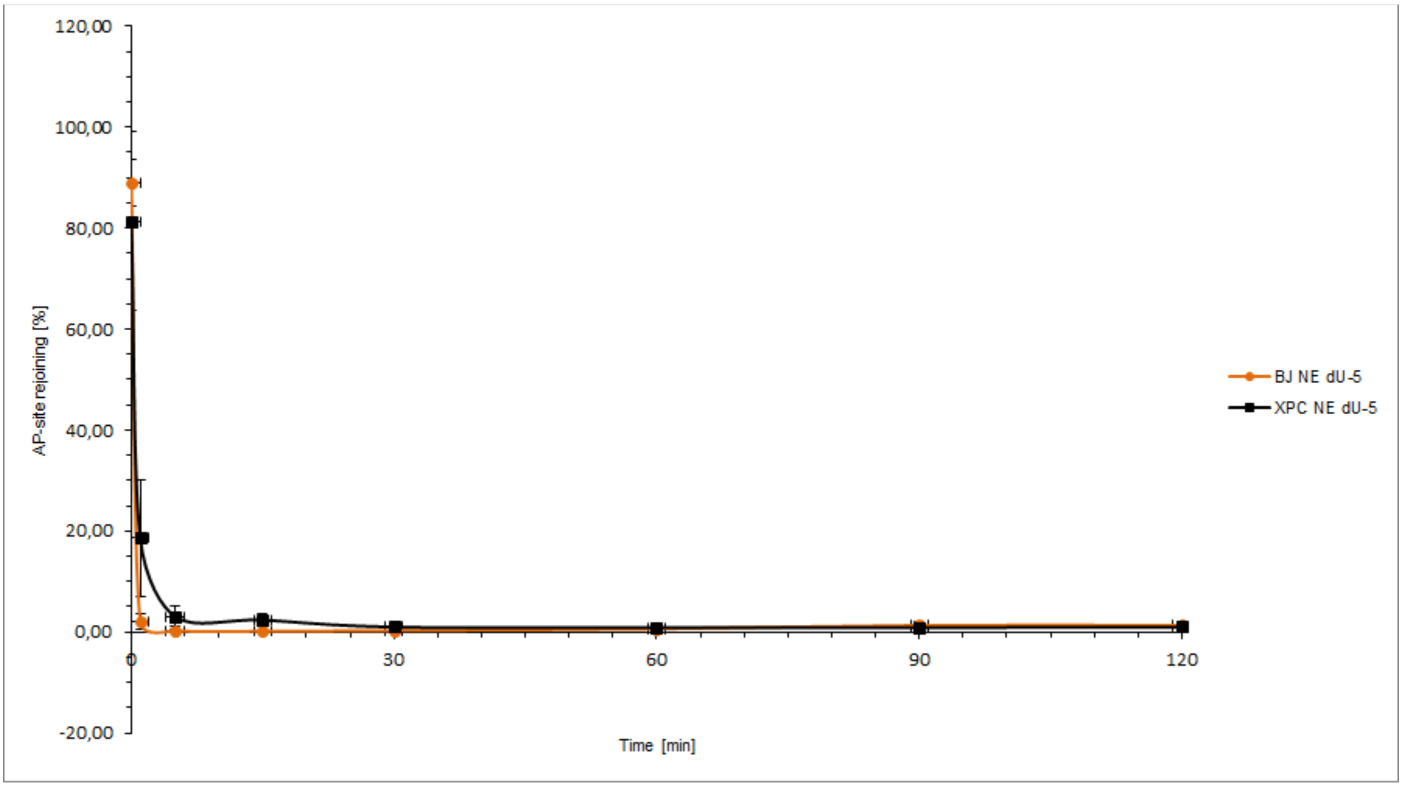

120,00
100,00
80,00
60,00
40,00
20,00
0,00
-20,00
AP-site rejoining [%]
0
30
60
90
120
Time [min]
BJ NE dU-5
XPC NE dU-5

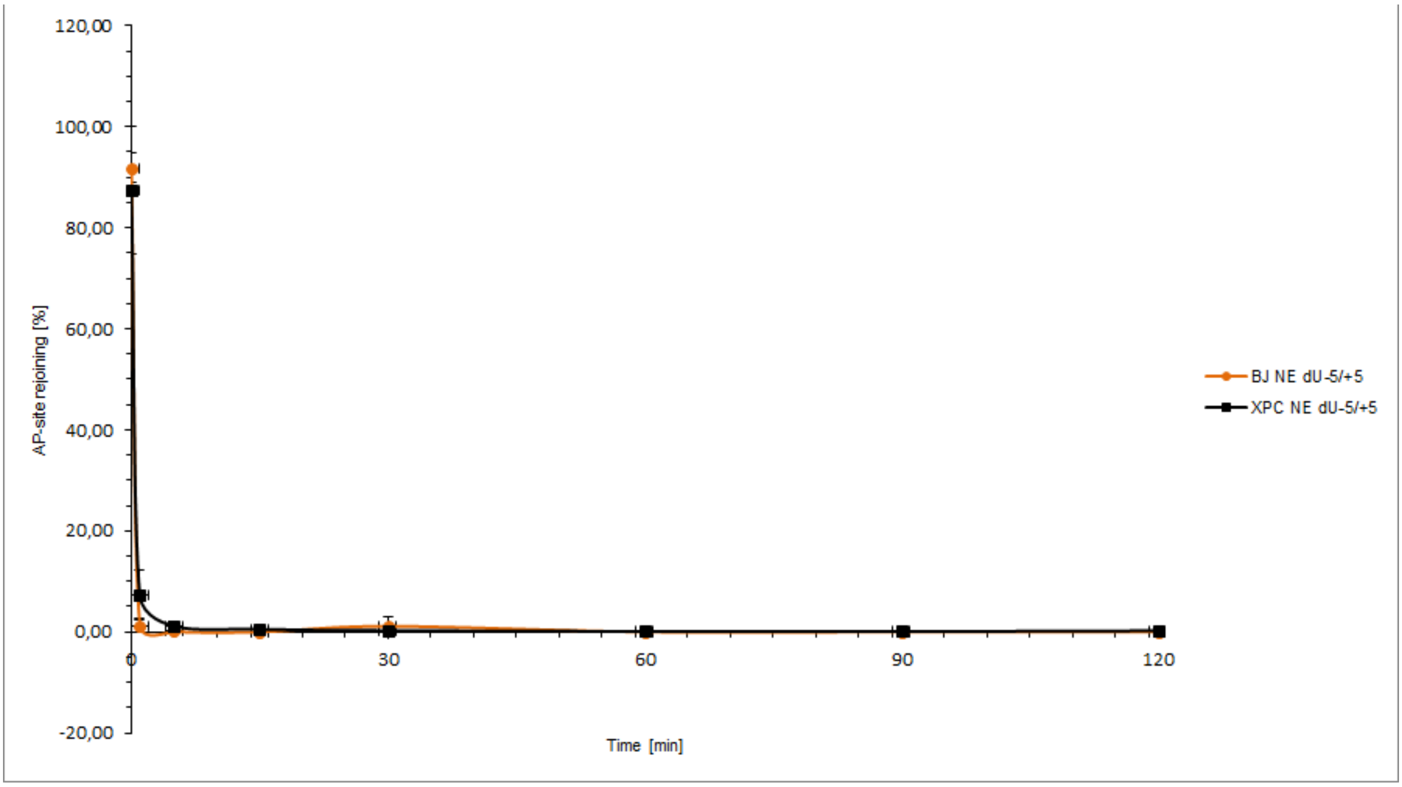

120,00
100,00
80,00
60,00
40,00
20,00
0,00
-20,00
AP-site rejoining [%]
0
30
60
90
120
Time [min]
BJ NE dU-5/+5
XPC NE dU-5/+5

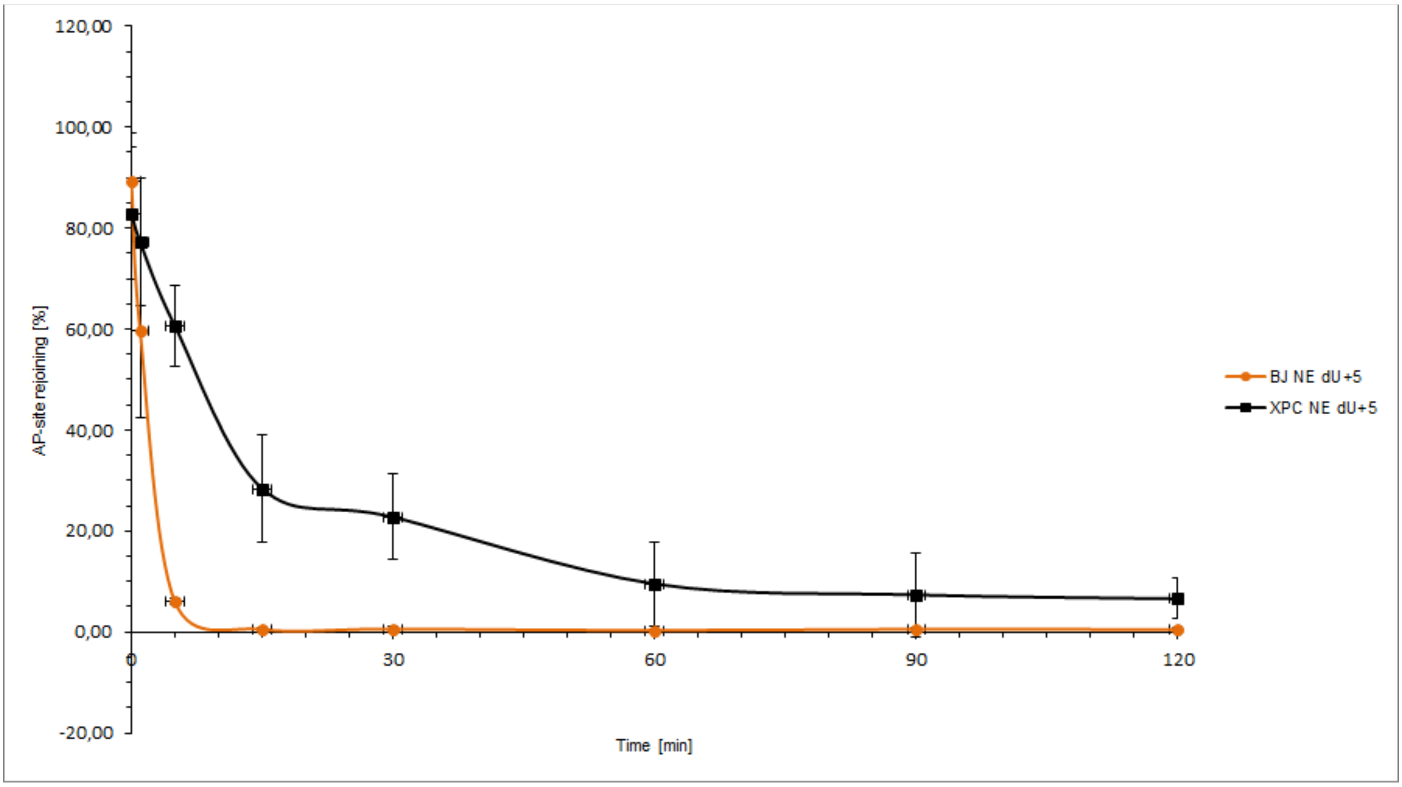

120,00
100,00
80,00
60,00
40,00
20,00
0,00
-20,00
AP-site rejoining [%]
0
30
60
90
120
Time [min]
BJ NE dU+5
XPC NE dU+5

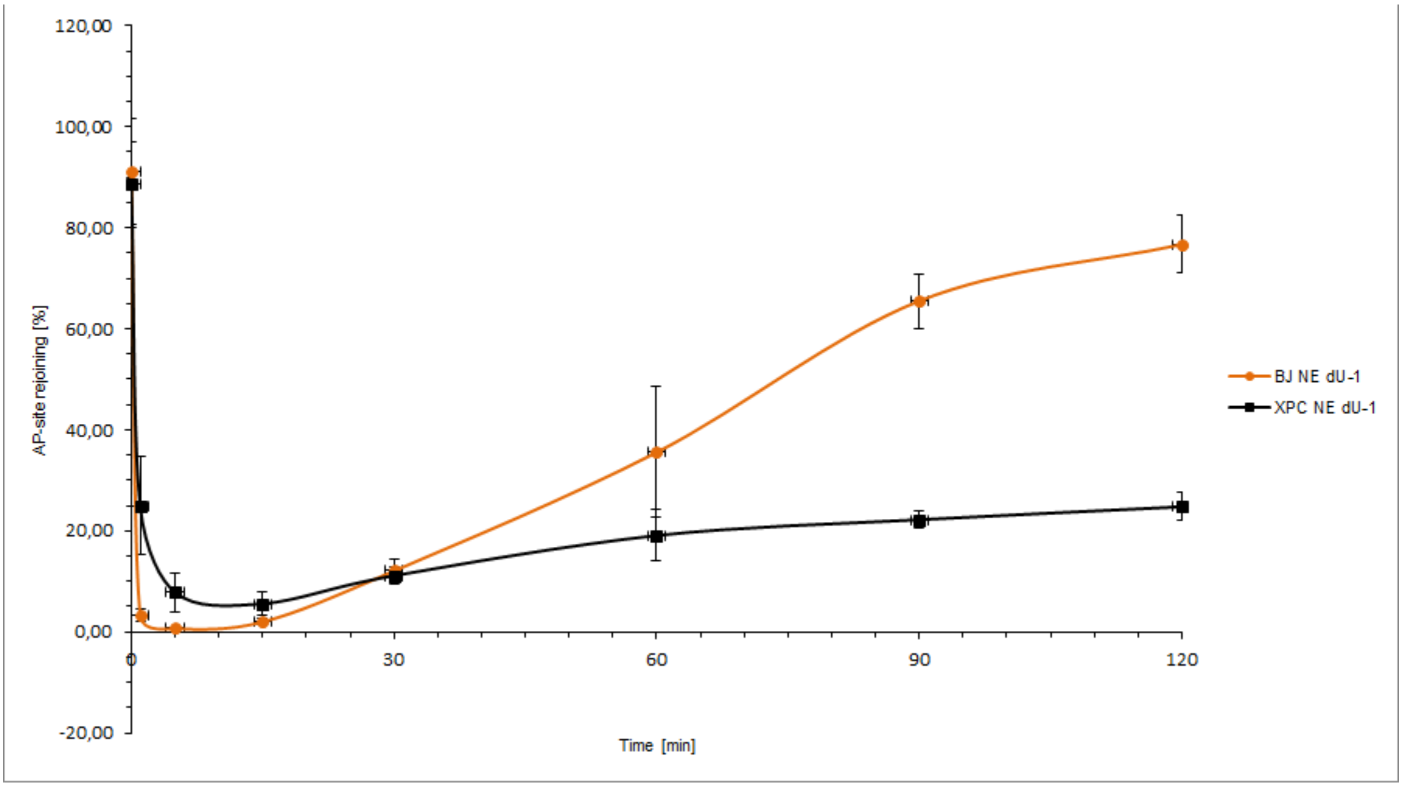
120,00
100,00
80,00
60,00
40,00
20,00
0,00
-20,00
AP-site rejoining [%]
0
30
60
90
120
Time [min]
BJ NE dU-1
XPC NE dU-1

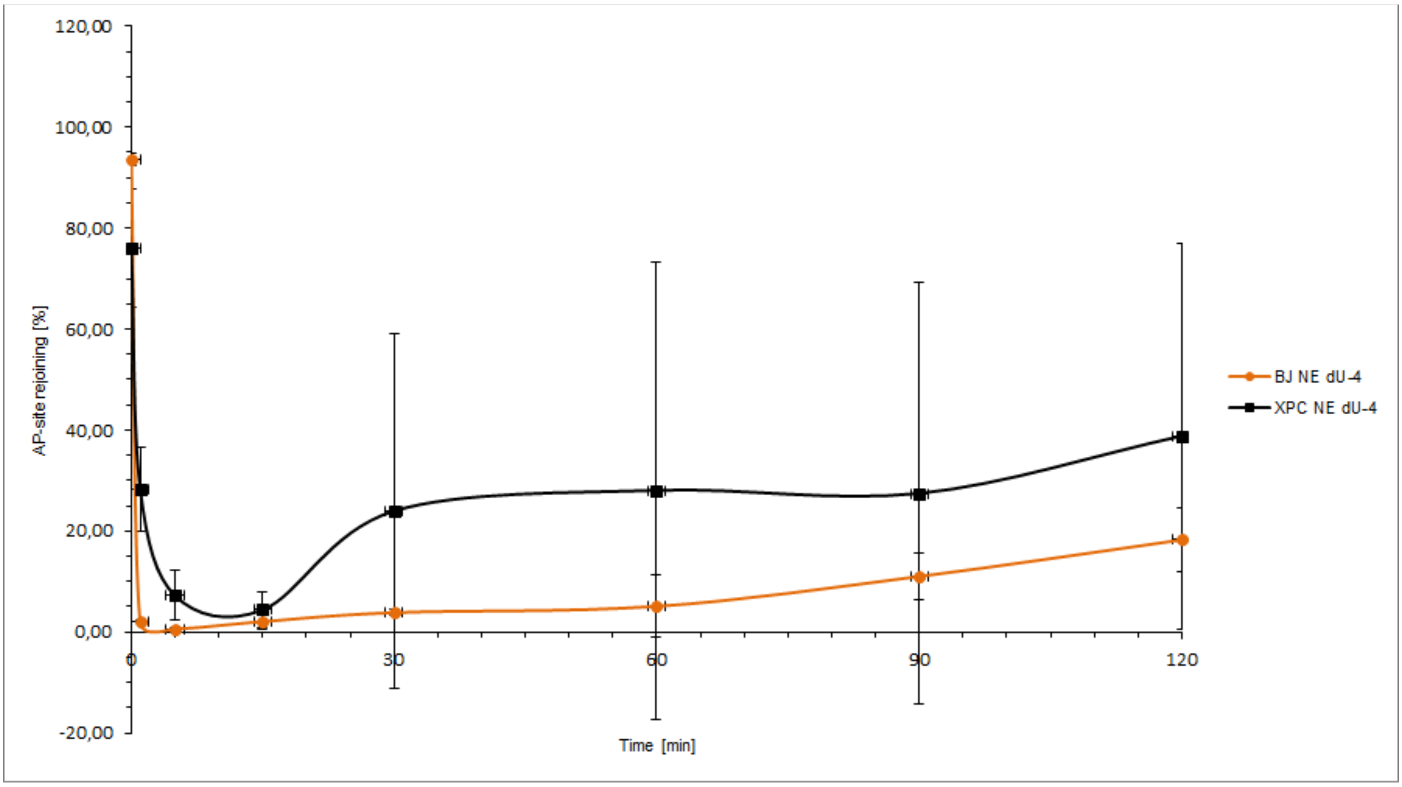
120,00
100,00
80,00
60,00
40,00
20,00
0,00
-20,00
AP-site rejoining [%]
0
30
60
90
120
Time [min]
BJ NE dU-4
XPC NE dU-4

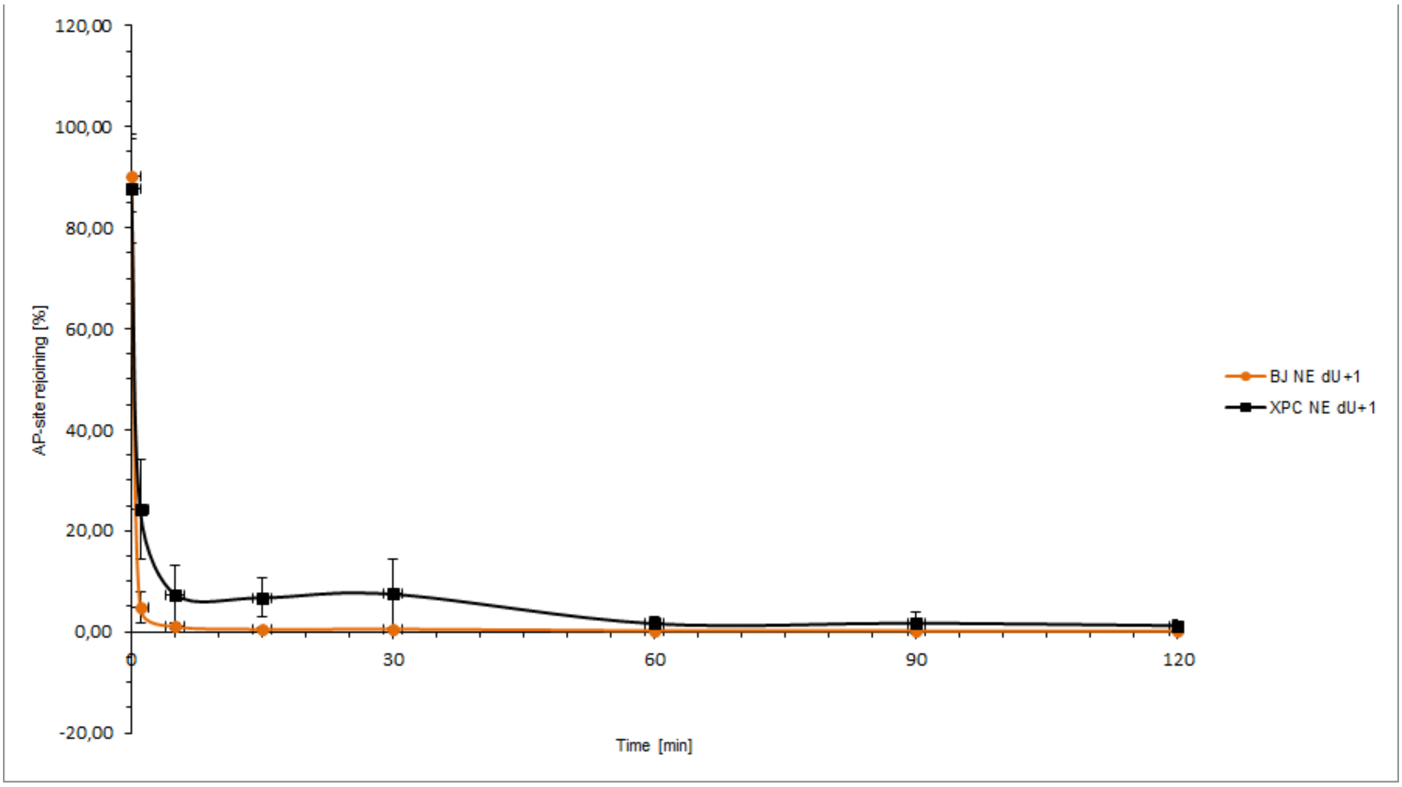

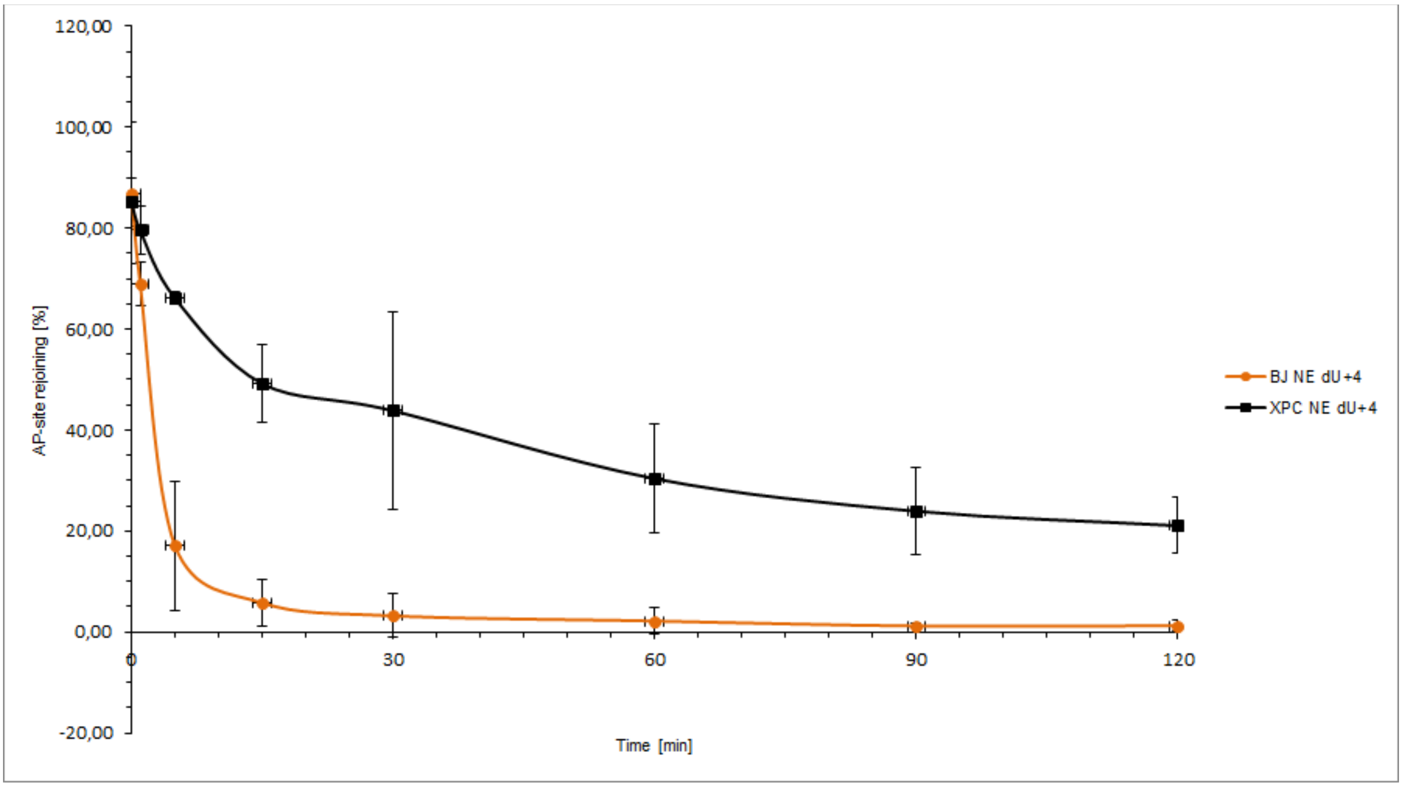

Figure S14. AP site rejoining [%] of BJ vs. XPC – comparison of individual strands – RcdA Nuclear Extract.
